# Investigating the Influence of Steric Hindrance on Selective Anion Transport

**DOI:** 10.3390/molecules24071278

**Published:** 2019-04-02

**Authors:** Laura A. Jowett, Angela Ricci, Xin Wu, Ethan N. W. Howe, Philip A. Gale

**Affiliations:** 1 School of Chemistry (F11), The University of Sydney, Sydney, NSW 2006, Australia; ljow8193@uni.sydney.edu.au (L.A.J.); xin.wu@sydney.edu.au (X.W.); ethan.howe@sydney.edu.au (E.N.W.H.); 2 Chemistry, University of Southampton, Southampton SO17 1BJ, UK; angela.ricci20@gmail.com; 3 Department of Pure and Applied Sciences, Chemistry Section, Universita Degli Studi Di Urbino “Carlo Bo”, via della Stazione 4, 61029 Urbino PU, Italia

**Keywords:** anion transport, thioureas, lipid bilayer

## Abstract

A series of symmetrical and unsymmetrical alkyl tren based tris-thiourea anion transporters were synthesised and their anion binding and transport properties studied. Overall, increasing the steric bulk of the substituents resulted in improved chloride binding and transport abilities. Including a macrocycle in the scaffold enhanced the selectivity of chloride transport in the presence of fatty acids, by reducing the undesired H^+^ flux facilitated by fatty acid flip-flop. This study demonstrates the benefit of including enforced steric hindrance and encapsulation in the design of more selective anion receptors.

## 1. Introduction

Anion transporters that can restore chloride flux through apical epithelial cell membranes have potential to function as ‘channel replacement therapies’ to ameliorate the symptoms of diseases, such as cystic fibrosis (CF) [1,2,3,4,5,6,7]. CF is a channelopathy, where a genetic mutation to the cystic fibrosis transmembrane conductance regulator (CFTR) protein results in a malfunctioning chloride channel [8,9,10]. A reduced level of anion (Cl^−^ and HCO_3_^−^) transport occurs, leading to a lower level of osmosis. As a result, sticky mucous secretions occur which results in inflammation and infections particularly in the lungs [11]. Synthetic chloride transporters have the potential to bypass the faulty channel and restore activity by independently diffusing across the membrane as a chloride complex. Our research group has recently focused on designing transporters that are selective for chloride over proton transport [12], as dissipation of pH gradients in cells has been shown to trigger apoptosis [13,14,15], and may disrupt the autophagic process [16,17]. Therefore, the development of selective chloride transporters that do not facilitate H^+^/OH^−^ transport is an attractive goal in the development of putative channel replacement therapies.

Recently, we discovered that anionophores can act as functional mimics of uncoupling proteins, and facilitate H^+^ transport via facilitated fatty acid transport [18]. Receptors can complex the negatively charged fatty acid carboxylate head group and mediate its passage through the lipid bilayer membrane. Decomplexation occurs, the free fatty acid protonates and diffuses back across the membrane, thus resulting in proton transport. The rate determining step in the transport cycle is diffusion of the free receptor, while fatty acid flip-flop provides an alternate pathway through the membrane for anion transporters, which results in an increased rate of H^+^/Cl^−^ symport. The increase in H^+^ transport via this mechanism is undesirable in the case of chloride selective receptors for the treatment of CF. 

**Figure 1:**
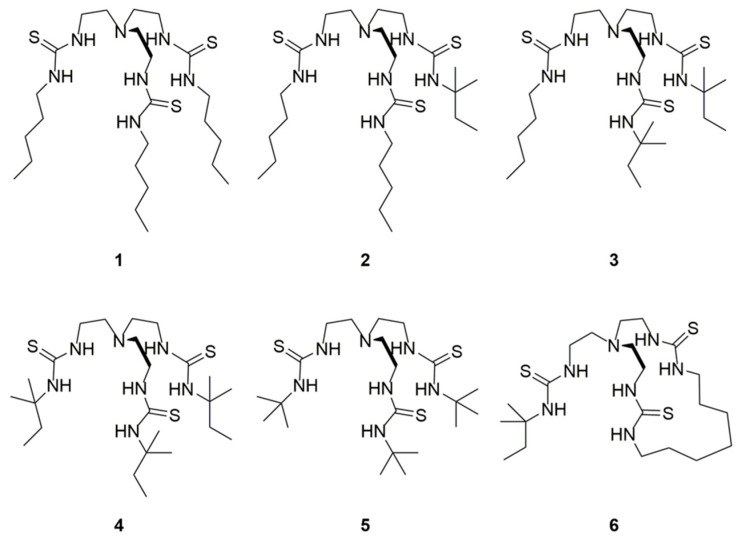
Structure of receptors **1**–**6**.

The challenge is to design receptors capable of chloride uniport that do not facilitate either the transport of H^+^/OH^−^ or fatty acid flip-flop. We have shown with a series of strapped calixpyrroles that increasing the degree of encapsulation of a receptor results in a high degree of chloride selectivity, which is not compromised in the presence of fatty acids [19]. To further investigate this phenomenon, as well as the effects of steric hindrance and preorganisation of anionophores on Cl^−^ > H^+^/OH^−^ selective transport, a series of receptors, shown in Figure 1, based on the tren scaffold were developed. Previously, Wu et. al. [12] reported the transport selectivity of *n*-pentyl, **1**, and *t*-pentyl, **4**, substituted tren compounds, and this inspired the study of two additional constitutional isomers, **2** and **3**, with varying ratios of *n*-pentyl and *t*-pentyl substituents. Receptor **5**, with *t*-butyl groups, was created to explore whether there is an optimum size for substituents. A macrocycle was incorporated into the tren scaffold for receptor **6**, and the macrocycle may encapsulate the binding site to a greater extent [20]. Furthermore, preorganisation of the thiourea anion binding groups could result in increased anion binding affinities, which could potentially lead to improved anion transport abilities.

In this paper, we report the synthesis of symmetrical, **5**, and unsymmetrical tren based thiourea anionophores **2**, **3**, and **6**. The anion binding properties of the whole series of receptors were studied with a range of biologically relevant anions. Elucidation of the transport mechanism was achieved using cationophore coupled assays, and a full examination of the chloride transport activity as well as investigations into the Cl^−^ > H^+^/OH^−^ transport selectivity were performed. 

## 2. Results and Discussion

### 2.1. Synthesis

**Figure 2:**
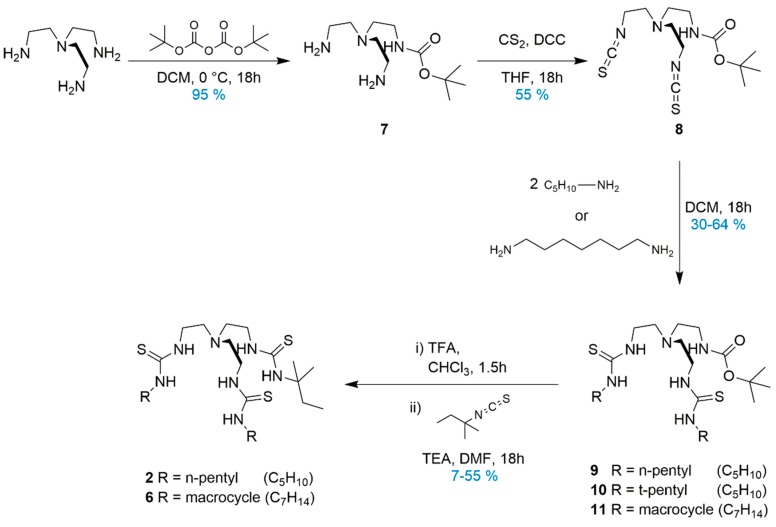
Scheme of the synthetic procedure for unsymmetrical and macrocyclic tren-based thiourea receptors.

The synthesis of **1** and **4** was previously reported [12] and using the same method, receptor **5** was synthesised in an 82% yield. Compounds **2**, **3**, and **6** were synthesised via a multi-step procedure, which is shown in Figure 2. In all cases, mono-Boc protection of tris-(2-aminoethyl)amine was performed to give **7**, followed by transformation of the two free amines to isothiocyanate groups in **8**. Intermediate **8** was reacted with either *n*-pentyl amine, *t*-pentyl amine, or di-aminoheptane to obtain the Boc-protected product of receptors **2**, **3**, and **6**, intermediates **9**–**11**, respectively. From here, Boc-deprotection and subsequent coupling with the appropriate alkyl thiourea gave receptors **2** and **6** in 55% and 7% yields. However, these conditions were repeatedly unsuccessful in the case of receptor **3**, therefore, an alternate route was employed. Tris-(2-aminoethyl)amine was reacted directly with *t*-pentyl isothiocyanate in a 1:2 molar ratio to favour formation of the di-substituted product. After separation of the desired di-*t*-pentyl mono-amine tren by column chromatography, coupling with the *n*-pentyl thiourea gave receptor **3** in an 83% yield. Full synthetic details and characterisation can be found in the Supplementary Material. 

### 2.2. Anion Binding Studies

The ability of receptors **1**–**6** to bind biologically relevant anions in solution was investigated using ^1^H NMR titrations in DMSO-*d*_6_/0.5% H_2_O, with the anion added as either the tetrabutylammonium (TBA) or tetraethylammonium (TEA) salt. Where possible, the change in the chemical shift of more than one resonance was followed, allowing global fitting of the titration data to a 1:1 binding model using Bindfit [21]; summarised in Table 1. 

The chloride binding affinity increases for the more sterically hindered receptors through the series. The strongest binding to chloride was observed for the macrocyclic receptor **6**, presumably due to a preorganisation effect. As expected, the overall binding affinity to bicarbonate was stronger than chloride with the macrocycle, **6**, again exhibiting the highest binding affinity. However, for receptors **1**–**5**, the opposite trend was evident, and the increased steric bulk of receptors resulted in a decrease in binding affinity to the larger bicarbonate anion through the series. Peak broadening occurred during titrations with dihydrogen phosphate and therefore association constants could not be determined for receptors **1**–**3** and **6**. Very high binding affinity was observed between receptors **4** and **5** with dihydrogen phosphate.

### 2.3. Anion Transport Studies

#### 2.3.1. Transport Mechanism

Complementary cationophore coupled assays have been used to determine the transport mechanism of several classes of receptors [22,23,24]. The assay uses naturally occurring cationophores, valinomycin and monensin, that facilitate potassium transport by different mechanisms. Valinomycin is an electrogenic transporter and this process involves the net flow of charge across a membrane [25]. Selective potassium transport facilitated by valinomycin can dissipate the membrane potential produced by chloride uniport facilitated by electrogenic anion receptors, leading to an overall KCl efflux. In contrast, monensin functions as an electroneutral transporter and during the transport process, the charge is balanced by back transport of a species with the same charge [26]. Deprotonation of a carboxylic acid group occurs upon metal complexation, allowing K^+^/H^+^ exchange, which couples to the H^+^/Cl^−^ symport (or functionally equivalent Cl^−^/OH^−^ antiport) facilitated by an electroneutral receptor, resulting in overall KCl efflux. 

**Figure 3:**
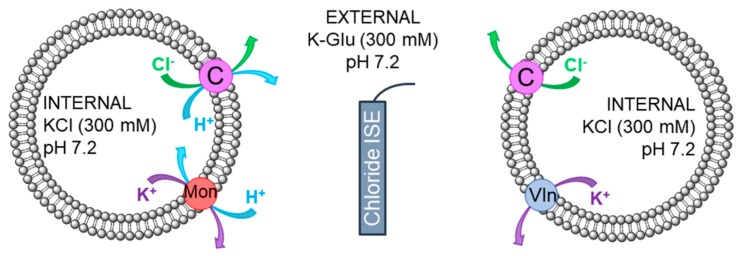
Overview of the complementary cationophore coupled assay. POPC vesicles (grey) contain an internal KCl (300 mM) solution and are suspended in an external K-Glu (300 mM) solution both buffered to pH 7.2 with HEPES. KCl efflux monitored by a chloride ISE (navy) induced by valinomycin (Vln, blue) facilitated electrogenic K^+^ transport coupled to compound (C, purple) mediated Cl^−^ uniport (right), and monensin (Mon, red) facilitated electroneutral K^+^/H^+^ exchange coupled to compound (C, purple) mediated Cl^−^/H^+^ symport (left).

To set up the assay detailed in Figure 3, large unilamellar vesicles (LUVs, 200 nm) composed of 1-palmitoyl-2-oleoyl-*sn*-glycero-3-phosphocholine (POPC, 200 nm diameter) were prepared containing potassium chloride (KCl, 300 mM) internal solution, and suspended in an external solution of potassium gluconate (K-Glu, 300 mM), both buffered to pH 7.2 with 4-(2-hydroxyethyl)piperazine-1-ethanesulfonic acid (HEPES) buffer (5 mm). As DMSO solutions, cationophores were added at t = −30 s followed by receptors at t = 0 s, and the resulting chloride efflux was monitored using a chloride ion selective electrode (ISE). At t = 300 s, the vesicles were lysed with detergent to give a measurement corresponding to a 100% chloride efflux, allowing calibration of the results. DMSO was tested as a control and no chloride efflux was detected, therefore the integrity of the vesicles is not affected by the amount of DMSO used. 

**Figure 4:**
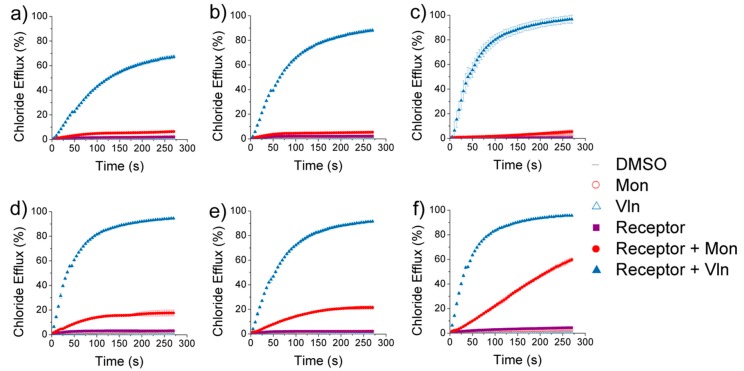
Results from the cationophore coupled assay. Chloride efflux facilitated by receptor (filled purple), receptor coupled to valinomycin (0.1 mol%, filled blue), and receptor coupled to monensin (0.1 mol%, filled red). The cationophore was added at t = −30 s, the receptor was added at t = 0 s, and detergent was added at t = 300 s. DMSO (grey), valinomycin (empty blue), and monensin (empty red) were tested as controls and each data point is the average of two repeats with the error bars showing the standard deviation. (**a**) Receptor **1** (0.1 mol%). (**b**) Receptor **2** (0.1 mol%). (**c**) Receptor **3** (0.1 mol%). (**d**) Receptor **4** (0.1 mol%). (**e**) Receptor **5** (0.1 mol%). (**f**) Receptor **6** (0.5 mol%).

Receptors **1**–**6** were tested in the cationophore coupled assay, and the results are shown in Figure 4. In all cases, ~100% chloride efflux was achieved in the presence of valinomycin. Receptors **1**–**5** displayed a small degree (<20%) of chloride efflux when coupling to monensin. Due to inactivity at the initial concentration (0.1 mol%, results in Supplementary Material), receptor **6** was tested at a higher concentration (0.5 mol%). An increase in electroneutral H^+^/Cl^−^ symport to ~60% was observed for receptor **6** in the presence of monensin. The results indicated that receptors **1**–**6** function predominantly as electrogenic chloride transporters, and macrocyclic receptor **6** is capable of some electroneutral chloride transport at higher receptor concentrations.

#### 2.3.2. Chloride Transport Selectivity

Quantification of the transport ability and Cl^−^ > H^+^/OH^−^ selectivity of receptors **1**–**6** was achieved using a modified NMDG-Cl assay [12,27]. Three previously optimized conditions [18] were employed. Firstly, (a) in bovine serum albumin (BSA) (1 mol%) treated vesicles (BSA was used to sequester any fatty acids present in the vesicles giving a fatty acid free lipid bilayer). Secondly, (b) in the presence of gramicidin (Gra, 0.1 mol%) added to facilitate electrogenic H^+^ efflux, enabling the study of receptor mediated Cl^−^ uniport. Finally, (c) in the presence of the naturally occurring fatty acid, oleic acid (OA, 2 mol%), which is found in cell membranes and can artificially enhance the overall rate of H^+^/Cl^−^ symport. Anion transporters can accelerate the fatty acid flip-flop mechanism by taking on the role of a flippase for fatty acid anions. Receptors bind to the anionic carboxylate head group and mask the charge, allowing rapid passage of the head group through the lipid bilayer. The carboxylate group can then protonate and diffuse back across the membrane and subsequently deprotonate, effectively resulting in H^+^ transport. Dose response studies and subsequent Hill analysis [28] for the three conditions detailed allowed the calculation of EC_50_ values to give the effective concentration of transporter required to achieve 50% chloride efflux from the vesicles. The results are shown in Table 2.

**Figure 5:**
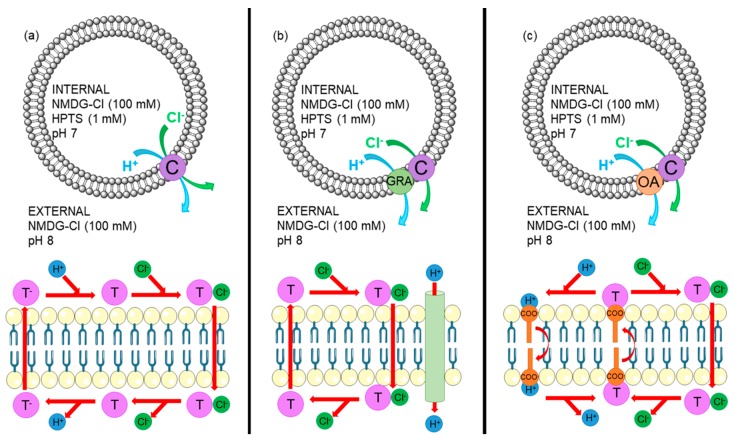
Top: Overview of the NMDG-Cl assay. POPC vesicles (grey) contain an internal NMDG-Cl (100 mM) and HPTS (1 mM) solution and are suspended in an external NMDG-Cl (100 mM) solution both buffered to pH 7.2 with HEPES (10 mM). HCl efflux, monitored by pH sensitive HPTS, induced by (**a**) compound (C, purple) mediated Cl^−^/H^+^ symport, (**b**) gramicidin (Gra, green) facilitated electrogenic H^+^ efflux coupled to compound (C, purple) mediated Cl^−^ uniport, and (**b**) oleic acid (OA, orange) flip-flop facilitated H^+^ transport coupled to compound (C, purple) mediated Cl^−^/H^+^ symport. Bottom: (**a**) Compound mediated Cl^−^/H^+^ symport via receptor deprotonation. The neutral receptor (T, purple) binds an anion (Cl^−^, green). The negatively charged complex moves across the membrane, and the anion dissociates. The receptor is deprotonated (H^+^, blue), and the negatively charged receptor moves across the membrane. The receptor is protonated to complete the cycle. (**b**) Compound mediated Cl^−^ uniport. The neutral receptor (T, purple) binds an anion (Cl^−^, green). The negatively charged complex moves across the membrane, and the anion dissociates. Gramicidin (Gra, green) facilitates electrogenic H^+^ efflux to balance the charge gradient build up. The neutral receptor diffuses back across the membrane to complete the cycle. (**c**) Compound mediated Cl^−^/H^+^ symport coupled to fatty acid flip-flop pathway. The neutral receptor (T, purple) binds an anion (Cl^−^, green) and transports it through the membrane as the negatively charged complex. The anion dissociates on the exterior of the membrane. The neutral receptor binds to a negatively charged deprotonated fatty acid (COO^−^, orange) and moves across the membrane as the negatively charged complex. Fatty acids can translocate across the membrane when protonated (H^+^, blue) to complete the cycle.

POPC LUVs (200 nm) were loaded with *N*-methyl-*D*-glucamine chloride (NMDG-Cl, 100 mM) and 8-hydroxypyrene-1, 3, 6-trisulfonic acid (HPTS, a pH sensitive dye, 1 mM), and suspended in an external solution of NMDG-Cl. All solutions were buffered to pH 7 with HEPES (10 mM). Receptors were added as a DMSO solution at t = −15 s, and transport was initiated by the addition of an NMDG (0.5 M) base pulse at t = 0 s, which increased the external pH to 8. The fluorescence ratio of 8-hydroxypyrene-1,3,6-trisulfonic acid trisodium salt (HPTS) (basic form, λ_ex_ = 460 nm, λ_em_ = 510 nm, divided by the acidic form, λ_ex_ = 403 nm, λ_em_ = 510 nm) was monitored as the pH gradient across the membrane was dissipated by either: (a) Receptor mediated H^+^/Cl^−^ symport (or OH^−^/Cl^−^ exchange), (b) a combination of receptor mediated Cl^−^ uniport coupled to H^+^ efflux facilitated by Gra, and receptor mediated H^+^/Cl^−^ symport, or (c) a combination of receptor mediated Cl^−^ symport coupled to H^+^ efflux facilitated by fatty acid flip-flop, and receptor mediated H^+^/Cl^−^ symport. The assay conditions and transport pathways are shown in Figure 5.

Receptors **1**–**6** display poor H^+^/Cl^−^ symport abilities in BSA treated vesicles in the NMDG-Cl assay (Table 2). The low activity was expected, as the cationophore coupled assay showed these compounds predominantly function as electrogenic chloride transporters rather than electroneutral H^+^/Cl^−^ co-transporters. However, mirroring the trend in the chloride binding affinities, the EC_50_ values for receptors **1**–**4** decreased, indicating better transport abilities, as the encapsulation increases from additional *t*-pentyl substitution. Receptor **4** is the best transporter of this series with the lowest EC_50_ of 0.164 mol% in the BSA treated vesicles. *t*-Butyl substitution in receptor **5** results in the second worst transport ability in this series, suggesting that the more lipophilic pentyl substitution is favoured over butyl substitution. Although it displays the strongest chloride binding affinity, macrocyclic receptor **6** has the highest EC_50_ of the series. The poorer chloride transport is potentially due to the more rigid structure of the macrocycle, reducing the deliverability, and hindering passage of the free receptor as well as the receptor:chloride complex across the lipid bilayer. 

**Figure 6:**
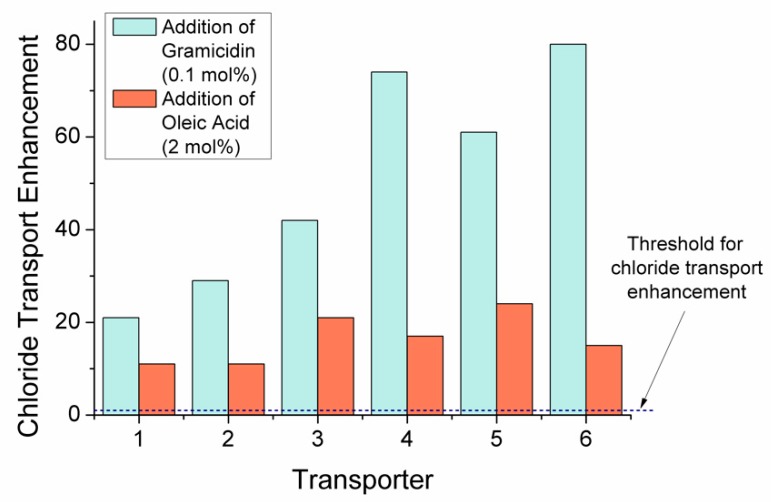
Chloride transport enhancement results. In the presence of Gra, a factor of chloride transport enhancement, F_Gra_, (green) is calculated by dividing the EC_50 (BSA)_ by the EC_50 (Gra)_. Factor of enhancement in the overall rate of Cl^−^/H^+^ cotransport in the presence of OA, F_OA_ (orange), is calculated by dividing the EC_50_ by the EC_50 (OA)_. Values over the threshold (>1) indicate an increase in the rate of chloride transport in the conditions described.

Including the proton channel, Gra, in the NMDG-Cl assay accelerates H^+^ efflux across the membrane. Therefore, the rate-limiting H^+^ transport step for electrogenic receptors **1**–**6** is removed, and the rate of chloride uniport is increased. A decrease in EC_50_ values by over one order of magnitude in all cases indicated an increase in chloride transport. A factor of enhancement in chloride transport (F_Gra_) was then quantified, by dividing (a) EC_50 (BSA)_ by (b) EC_50 (Gra)_. Values > 1 demonstrate an enhancement of the chloride transport, and the results are shown in Table 2. A bar chart is also included for clarity in Figure 6. Calculated F_Gra_ values showed impressive enhancement in chloride transport for all receptors. The most encapsulating receptors, **4**–**6**, displayed the highest F_Gra_ values, and macrocyclic receptor **6** showed the largest enhancement in chloride transport of the series. The same trend was repeated for receptors **1**–**4**. Increasing encapsulation from additional *t*-pentyl substituents resulted in higher F_Gra_ values, corresponding to increased chloride uniport.

The addition of OA to the NMDG-Cl assay allowed an evaluation of the receptors’ ability to contribute to the naturally occurring fatty acid transmembrane proton shuttling pathway, by assisting the flip-flop of the negatively charged fatty acid head group. The pathway is depicted in Figure 4. Dividing (a) EC_50 (BSA)_ by (c) EC_50 (OA)_ allowed a factor of enhancement (F_OA_) in the overall rate of H^+^/Cl^−^ transport in the presence of OA to be calculated. The results shown in Table 2 and Figure 6, give F_OA_ values > 1 for all receptors, indicating that the overall rate of H^+^/Cl^−^ symport is increased due to receptor coupled fatty acid flip-flop. Enhancement factors are greater in the presence of Gra compared to OA. Therefore, receptors **1**–**6** preferentially facilitate chloride uniport compared to H^+^/Cl^−^ symport, reinforcing the electrogenic transport mechanism shown by receptors **1**–**6** in the cationophore coupled assay. 

In the presence of OA, chloride uniport is often diminished, because receptors can display some affinity towards the fatty acid headgroup [18]. As free fatty acids are abundant in cell membranes, reducing affinity for fatty acid headgroups is an important consideration in the development of anionophores for the potential treatment of CF. Therefore, an assessment of the ability of receptors **1**–**6** to retain chloride selective transport in the presence of OA was calculated. Assuming the chloride uniport achieved in the presence of Gra (i.e., EC_50 (Gra)_) corresponds to the chloride selective transport by receptors **1**–**6**, a factor of the chloride transport selectivity retention in the presence of OA (F_OA/Gra_) can be calculated. Dividing (c) EC_50 (OA)_ by (b) EC_50 (Gra)_ gives F_OA/Gra_ values, which can be found in Table 2. Values > 1 indicate a retention in chloride selective transport in the presence of OA. F_OA/Gra_ values of ~2 were obtained for receptors **1**–**3** and **5**, indicating a slight amount of chloride selective transport retention. Macrocyclic receptor **6** retained the highest degree of chloride selective transport in the presence of OA, F_OA/Gra_ = 5.4, with the most encapsulating receptor **4** also retaining chloride selective transport, F_OA/Gra_ = 4.3. The results demonstrate that it is advantageous to include enforced steric hindrance and encapsulation in the design of anion receptors to reduce affinity towards the fatty acid headgroup and retain chloride selective transport.

## 3. Conclusions

We have shown that increasing the steric hindrance of simple tren based receptors results in an increase in binding affinity towards chloride. However, the reduced structural freedom of these receptors results in a decrease in binding affinity towards the bicarbonate anion. From the cationophore coupled assay, it was evident that this series of tren-based tris-thiourea receptors function predominantly as electrogenic chloride transporters. The H^+^/Cl^−^ symport activity of this series was poor as to be expected, but chloride uniport in the presence of the proton channel, Gra, showed high activity. Enhancement of chloride transport in the presence of Gra was impressive over the whole series, however, all receptors were able to facilitate the undesirable fatty acid flip-flop proton transport pathway at different degrees. This unwanted effect was less apparent for the more sterically hindered receptors, **4** and **6**, demonstrating the benefit of encapsulating receptors. Overall, this study shows that an elaborated receptor design is crucial to minimise H^+^/OH^−^ transport and to obtain chloride selective transporters.

## Figures and Tables

**Table 1:** Stability constants (*K*_a_) for the complexation of receptors **1**–**6** with chloride, bicarbonate, and dihydrogen phosphate (as TBA salts for chloride and dihydrogen phosphate and TEA for bicarbonate) in DMSO-*d_6_*/0.5% H_2_O at 298 K.

Receptor	*K*_a_ (M^−1^) for 1:1 Receptor:Anion Complex		
	*K*_a_ Cl^− a^	*K*_a_ HCO_3_^− b^	*K*_a_ H_2_PO_4_^− b^
1	346	5010 ^c^	n.d. ^d^
2	770 ^e^	5560 ^f^	n.d. ^g^
3	1080 ^e^	2460 ^f^	n.d. ^g^
4	1530	1500	7460
5	1110	2670	2600
6	2170 ^b, e^	4930 ^h^	n.d. ^g^

All errors < 15%. ^a^ Host concentration 5 mM. ^b^ Host concentration 1 mM. ^c^ Fitted to 7.5 equiv. as peaks broadened and merged at 10 equiv. ^d^ No association constant determined, peaks broaden up to 1 eq due to strong binding, then peaks sharpen between 1–4 equiv. from formation of the 1:1 complex, above 4 equiv. peaks broaden signifying the presence of the 1:2 complex. ^e^ Two resonances followed and fitted. ^f^ Three resonances followed and fitted. ^g^ No association constant determined, peaks broaden up to 1 equiv., then peaks sharpen between above 1 equiv. ^h^ One resonance followed and fitted.

**Table 2:** EC_50_ values shown for the NMDG-Cl assay with receptors **1**–**6** in BSA treated vesicles, in the presence of gramicidin (Gra) and in the presence of oleic acid (OA).

Receptor	EC_50_ (mol%) ^a^			F_Gra_^e^	F_OA_^f^	F_OA/Gra_^g^
	(a) BSA ^b^	(b) Gra ^c^	(c) OA ^d^			
**1**	0.236	0.0113	0.0214	21	11	1.9
**2**	0.218	0.00748	0.0195	29	11	2.6
**3**	0.175	0.00420	0.00834	42	21	1.9
**4**	0.164	0.00223	0.00970	74	17	4.3
**5**	0.295	0.00483	0.0121	61	24	2.5
**6**	1.900	0.0238	0.126	80	15	5.4

Values obtained are concentration dependent and can change with different assay conditions, e.g., EC_50_ values of previously reported receptors **1** and **4** are lower due to the higher volume of DMSO solutions used [12,18]. ^a^ Values reported in transporter to lipid molar ratio (mol %). ^b^ Measures Cl^−^/H^+^ symport (Cl^−^/OH^−^ antiport) after vesicle treatment with BSA (1 mol%). ^c^ Measures total Cl^−^/H^+^ symport (Cl^−^/OH^−^ antiport), rate limiting H^+^ transport is facilitated by Gra (0.1 mol%). ^d^ Measures Cl^−^/H^+^ symport (Cl^−^/OH^−^ antiport), with H^+^ transport facilitated by the fatty acid flip-flop of OA (2 mol%). ^e^ Factor of enhancement in the Cl^−^ uniport in the presence of Gra, F_Gra_ is calculated by dividing the EC_50 (BSA)_ by the EC_50 (Gra)_. F_Gra_ > 1 indicates Cl^−^ transport enhancement. ^f^ Factor of enhancement in the overall rate of Cl^−^/H^+^ cotransport in the presence of OA, F_OA_ is calculated by dividing the EC_50_ by the EC_50 (OA)_. F_OA_ > 1 indicates the receptor can assist the flip-flop of OA^−^, increasing pH dissipation. ^g^ Factor of chloride transport selectivity retention in the presence of oleic acid, F_Gra/OA_ is calculated by dividing the EC_50 (OA)_ by the EC_50 (Gra)_. F_Gra/OA_ > 1 indicates Cl^−^ selectivity retention.

## Supplementary Materials

Synthetic procedures and compound characterizations, methodology and results for anion binding experiments, and methodology and results of anion transport experiments are available online at https://www.mdpi.com/1420-3049/24/7/1278/s1. Figures S1–S104.

## References

[1] Gale P.A., Davis J.T., Quesada R. (2017). Anion transport and supramolecular medicinal chemistry. Chem. Soc. Rev..

[2] Jentzsch A.V., Emery D., Mareda J., Nayak S.K., Metrangolo P., Resnati G., Sakai N., Matile S. (2012). Transmembrane anion transport mediated by halogen-bond donors. Nat. Commun..

[3] Li H., Valkenier H., Judd L.W., Brotherhood P.R., Hussain S., Cooper J.A., Jurček O., Sparkes H.A., Sheppard D.N., Davis A.P. (2016). Efficient, non-toxic anion transport by synthetic carriers in cells and epithelia. Nat. Chem..

[4] Hernando E., Capurro V., Cossu C., Fiore M., García-Valverde M., Soto-Cerrato V., Pérez-Tomás R., Moran O., Zegarra-Moran O., Quesada R. (2018). Small molecule anionophores promote transmembrane anion permeation matching CFTR activity. Sci. Rep..

[5] Valkenier H., Akrawi O., Jurček P., Sleziaková K., Lízal T., Bartik K., Šindelář V. (2019). Fluorinated Bambusurils as Highly Effective and Selective Transmembrane Cl^−^/HCO_3_^−^ Antiporters. Chem.

[6] Lee L.M., Tsemperouli M., Poblador-Bahamonde A.I., Benz S., Sakai N., Sugihara K., Matile S. (2019). Anion Transport with Pnictogen Bonds in Direct Comparison with Chalcogen and Halogen Bonds. J. Am. Chem. Soc..

[7] Fiore M., Cossu C., Capurro V., Picco C., Ludovico A., Mielczarek M., Carreira-Barral I., Caci E., Baroni D., Quesada R. (2019). Small molecule-facilitated anion transporters in cells for a novel cystic fibrosis therapeutic approach. Br. J. Pharmacol..

[8] Ashcroft F.M. (2000). Ion Channels and Disease.

[9] Vankeerberghen A., Cuppens H., Cassiman J.-J. (2002). The cystic fibrosis transmembrane conductance regulator: An intriguing protein with pleiotropic functions. J. Cyst. Fibros..

[10] Vergani P., Lockless S.W., Nairn A.C., Gadsby D.C. (2005). CFTR channel opening by ATP-driven tight dimerization of its nucleotide-binding domains. Nature.

[11] Gadsby D.C., Vergani P., Csanády L. (2006). The ABC protein turned chloride channel whose failure causes cystic fibrosis. Nature.

[12] Wu X., Judd L.W., Howe E.N.W., Withecombe A.M., Soto-Cerrato V., Li H., Busschaert N., Valkenier H., Pérez-Tomás R., Sheppard D.N. (2016). Nonprotonophoric Electrogenic Cl^−^ Transport Mediated by Valinomycin-like Carriers. Chem.

[13] Soto-Cerrato V., Llagostera E., Montaner B., Scheffer G.L., Pérez-Tomás R. (2004). Mitochondria-mediated apoptosis operating irrespective of multidrug resistance in breast cancer cells by the anticancer agent prodigiosin. Biochem. Pharmacol..

[14] Tsukimoto M., Harada H., Ikari A., Takagi K. (2005). Involvement of Chloride in Apoptotic Cell Death Induced by Activation of ATP-sensitive P2X7 Purinoceptor. J. Biol. Chem..

[15] Ko S.K., Kim S.K., Share A., Lynch V.M., Park J., Namkung W., Van Rossom W., Busschaert N., Gale P.A., Sessler J.L. (2014). Synthetic ion transporters can induce apoptosis by facilitating chloride anion transport into cells. Nat. Chem..

[16] Hosogi S., Kusuzaki K., Inui T., Wang X., Marunaka Y. (2014). Cytosolic chloride ion is a key factor in lysosomal acidification and function of autophagy in human gastric cancer cell. J. Cell. Mol. Med..

[17] Busschaert N., Park S.-H., Baek K.-H., Choi Y.P., Park J., Howe E.N.W., Hiscock J.R., Karagiannidis L.E., Marques I., Félix V. (2017). A synthetic ion transporter that disrupts autophagy and induces apoptosis by perturbing cellular chloride concentrations. Nat. Chem..

[18] Wu X., Gale P.A. (2016). Small-Molecule Uncoupling Protein Mimics: Synthetic Anion Receptors as Fatty Acid-Activated Proton Transporters. J. Am. Chem. Soc..

[19] Clarke H.J., Howe E.N.W., Wu X., Sommer F., Yano M., Light M.E., Kubik S., Gale P.A. (2016). Transmembrane Fluoride Transport: Direct Measurement and Selectivity Studies. J. Am. Chem. Soc..

[20] Hancock R.D., Martell A.E. (1988). The Chelate, Cryptate and Macrocyclic Effects. Comments Inorg. Chem..

[21] Supramolecular.org—Online Tools for Supramolecular Chemistry Research and Analysis. http://supramolecular.org.

[22] Howe E.N.W., Busschaert N., Wu X., Berry S.N., Ho J., Light M.E., Czech D.D., Klein H.A., Kitchen J.A., Gale P.A. (2016). pH-Regulated Nonelectrogenic Anion Transport by Phenylthiosemicarbazones. J. Am. Chem. Soc..

[23] Jowett L.A., Howe E.N.W., Soto-Cerrato V., Van Rossom W., Pérez-Tomás R., Gale P.A. (2017). Indole-based perenosins as highly potent HCl transporters and potential anti-cancer agents. Sci. Rep..

[24] Jowett L.A., Howe E.N.W., Wu X., Busschaert N., Gale P.A. (2018). New Insights into the Anion Transport Selectivity and Mechanism of Tren-based Tris-(thio)ureas. Chem. Eur. J..

[25] Pressman B.C. (1976). Biological Applications of Ionophores. Annu. Rev. Biochem..

[26] Mollenhauer H.H., James Morré D., Rowe L.D. (1990). Alteration of intracellular traffic by monensin; mechanism, specificity and relationship to toxicity. Biochim. Biophys. Acta, Rev. Biomembr..

[27] Dawson R.E., Hennig A., Weimann D.P., Emery D., Ravikumar V., Montenegro J., Takeuchi T., Gabutti S., Mayor M., Mareda J. (2010). Experimental evidence for the functional relevance of anion–π interactions. Nat. Chem..

[28] Hill A.V. (1913). The Combinations of Haemoglobin with Oxygen and with Carbon Monoxide. I. Biochem. J..

