# Supplementary material for: Investigating the Influence of Steric Hindrance on Selective Anion Transport
Source: Molecules. 2019 Apr 2;24(7):1278. doi: 10.3390/molecules24071278 (PMC6480120; doi:10.3390/molecules24071278)
Supplement: Supplementary file 1 [file molecules-24-01278-s001.pdf]

1 Appendix

1.1 Alkyl Tren Series

1.1.1 Characterisation Data

^{1}H NMR and ^{13}C NMR shown for each compound. Low resolution mass spectrometry (LR-MS) shown for all compounds except 2-isothiocyanato-2-methylbutane where an IR is shown. High resolution mass spectrometry (HR-MS) shown for novel compounds.

1,1',1''-(Nitrilotris(ethane-2,1-diyl))tris(3-(tert-butyl)thiourea)

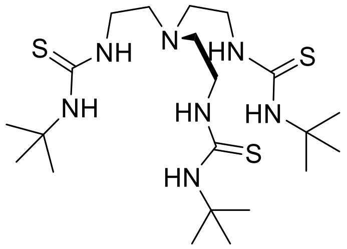

A solution of tris-(2-aminoethyl)amine (0.402 g, 2.7 mmol) in DCM (15 mL) was added with *tert*-butyl isothiocyanate (1.01 g, 8.8 mmol), the mixture was stirred under N_2 atmosphere at room temperature for 16 hr. The resulting clear pale yellow solution was concentrated to give a yellow residue, and was subsequently washed with DCM:hexane (1:5 v/v). The solvent was decanted, and the remaining yellow oily residue was recrystallised with DCM/Et_2O to afford off-white crystalline solids (1.104 g, 82%).

^{1}H NMR (400 MHz, DMSO-d_6) δ ppm 7.12 (s, 1 H) 7.05 (t, *J*=5.0 Hz, 1H) 3.47-3.42 (m, 2 H) 2.59 (t, *J*=6.7 Hz, 2 H) 1.40 (s, 9 H); **^{13}C NMR** (101 MHz, $CDCl_3$-*d*) δ ppm 181.15, 24.45, 52.09, 40.99, 28.94

LR-MS (ESI^+) *m/z* 492.37 $[M + H]^+$; **HR-MS** (ESI^+) calcd for $C_{21}H_{46}N_7S_3$ $[M+H]^+$: 492.2971, found *m/z* 492.2974

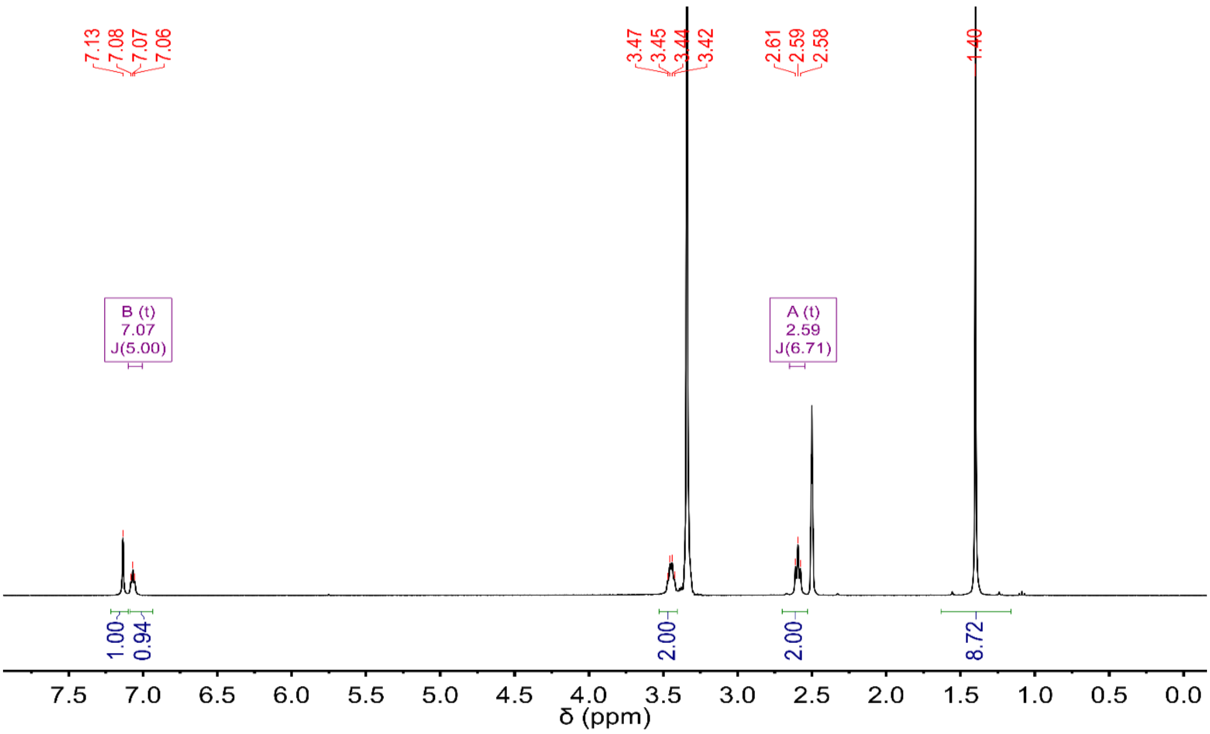

Figure 1. ^{1}H NMR (400 MHz) spectrum of 1,1',1''-(Nitrilotris(ethane-2,1-diyl))tris(3-(tert-butyl)thiourea) in $(CD_3)_2SO$ at 298 K.

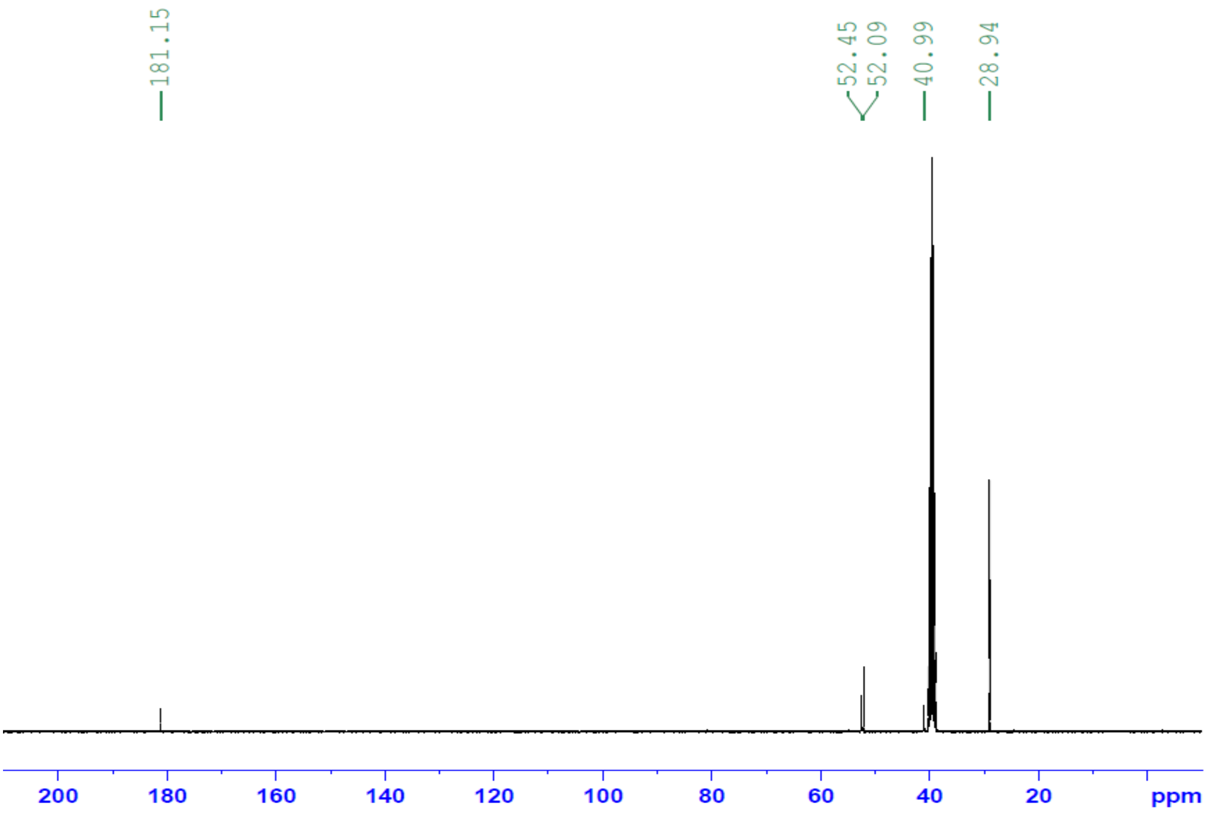

Figure 2. ^{13}C NMR (101 MHz) spectrum of 1,1',1''-(Nitrilotris(ethane-2,1-diyl))tris(3-(tert-butyl)thiourea) in $(CD_3)_2SO$ at 298 K.

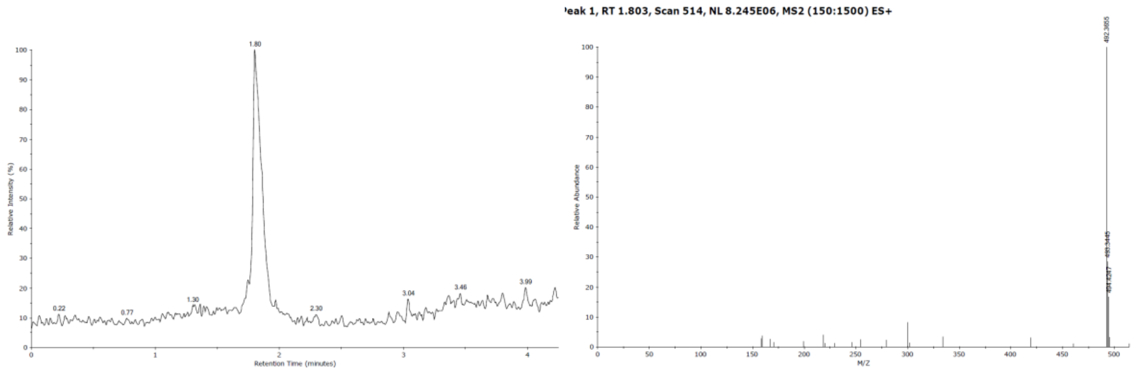

Figure 3. LR-MS (ESI$^+$) of 1,1',1''-(Nitrilotris(ethane-2,1-diyl))tris(3-(tert-butyl)thiourea).

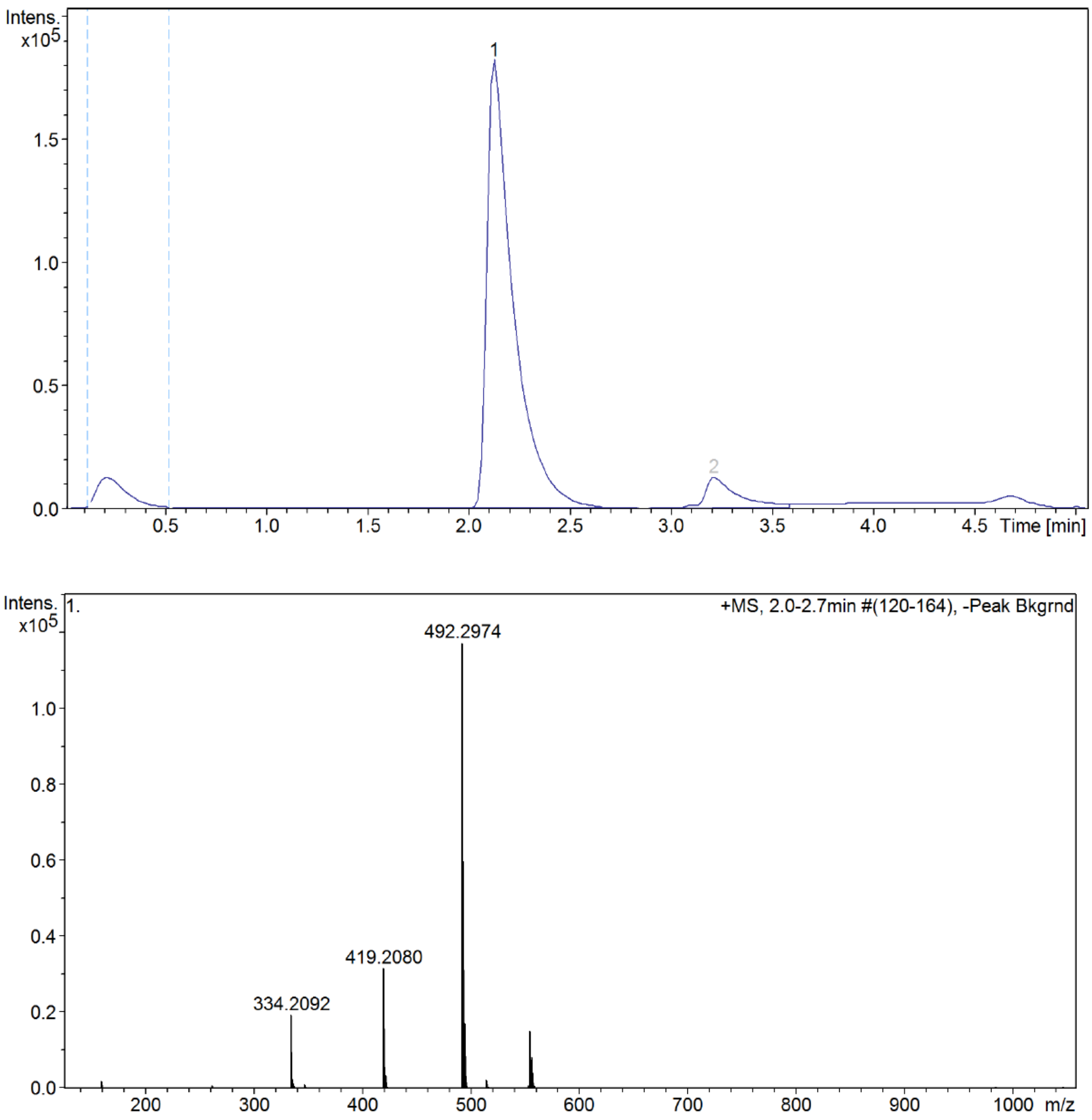

Figure 4. HR-MS (ESI$^+$) of 1,1',1''-(Nitrilotris(ethane-2,1-diyl))tris(3-(tert-butyl)thiourea).

2-Isothiocyanato-2-methylbutane

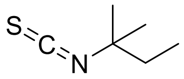

Thiophosgene (1.5 mL, 20 mmol) was dissolved in EtOAc (80 mL) and cooled to 0 ˚C. 2-Methyl-2-butanamine (7 mL, 60 mmol) was added dropwise over 30 mins keeping the solution stirring and on ice. K_2CO_3 (8.29 g, 60 mmol) was added to the ice-cold solution and then it was warmed to room temperature. The reaction was stirred at room temperature for 18 hr. After 18 hr water (100 mL) was added, the organic phase was washed with $NaHCO_3$ solution (4 x 200 mL), dried over $MgSO_4$ and solvent removed to leave an orange liquid. Purification by column chromatography (silica, DCM) gave an orange liquid (4.722 g, 60%).

^{1}H NMR (400 MHz, $CDCl_3$-*d*) δ ppm 1.65 (q, *J*=7.4 Hz, 2 H) 1.38 (s, 6 H) 1.01 (t, *J*=7.4 Hz, 3 H); **^{13}C NMR** (101 MHz, $CDCl_3$-*d*) δ ppm 162.48, 61.75, 36.25, 38.55, 9.02

IR ν_{max}/cm^{-1} 2976 (m, CH alkane), 2078 (s, N=C=S), 1460 (m, CH alkane)

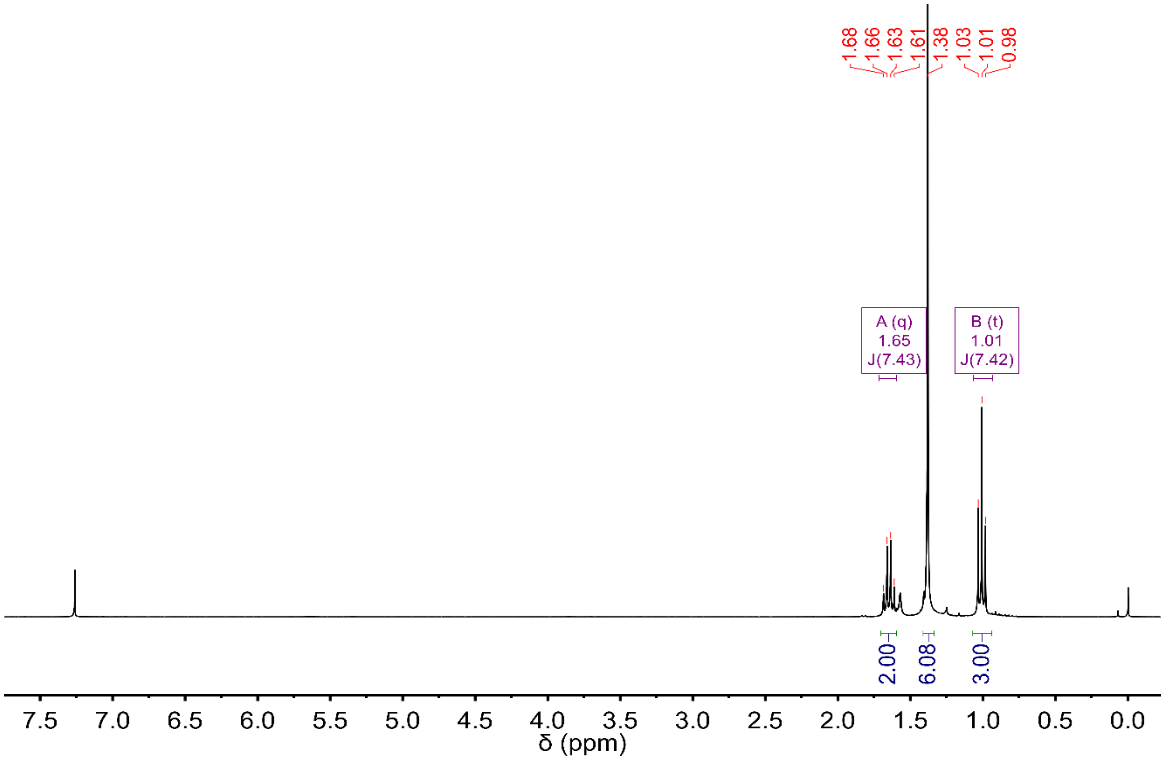

Figure 5. ^{1}H NMR (400 MHz) spectrum of 2-isothiocyanato-2-methylbutane in $CDCl_3$ at 298 K.

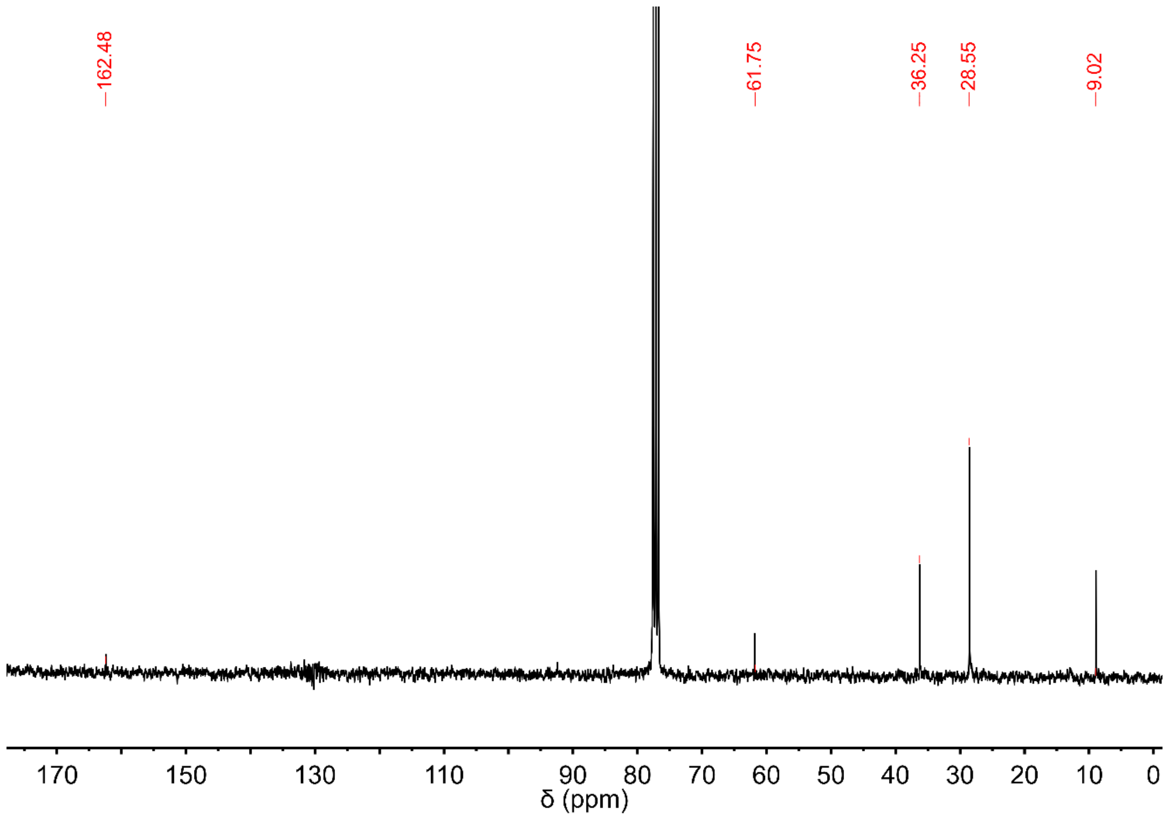

Figure 6. ^{13}C NMR (101 MHz) spectrum of 2-isothiocyanato-2-methylbutane in $CDCl_3$ at 298 K.

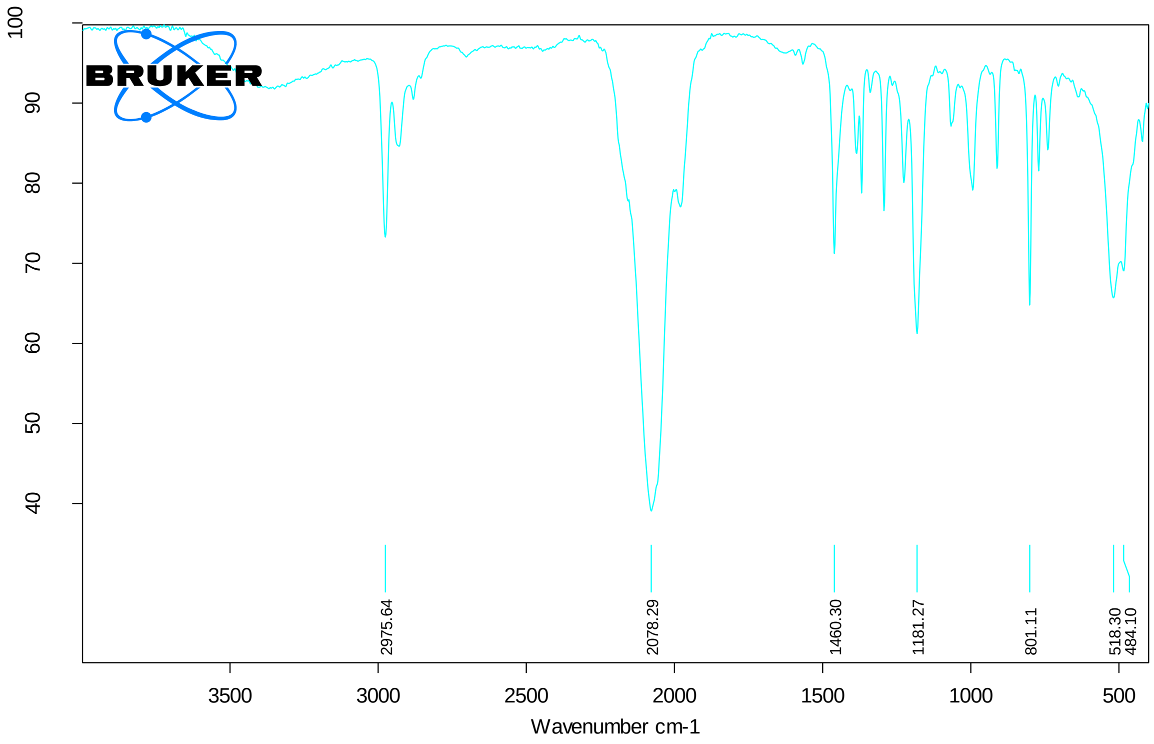

Figure 7. IR spectrum of 2-isothiocyanato-2-methylbutane.

1,1',1''-(Nitrilotris(ethane-2,1-diyl))tris(3-(tert-pentyl)thiourea)

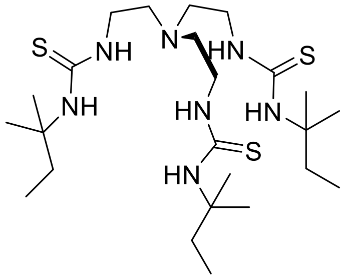

Tris-(2-aminoethyl)amine (0.15 mL, 1.03 mmol) was dissolved in anhydrous DCM (15 mL). 2-Isothiocyanato-2-methylbutane (0.40 g, 3.10 mmol) was dissolved in DCM (5 mL) and added dropwise to the initial solution. The mixture was stirred for 18 hr. After 18 hr solvent was removed and the crude product was purified by column chromatography (silica, DCM → 3% MeOH) to give a white crystalline solid (0.11 g, 20 %).

^{1}H NMR (500 MHz, DMSO d_6) δ ppm 7.10 (br s, 3 H) 6.95 (br s, 3 H) 3.44 (br s, 6 H) 2.60 (t, *J*=6.8 Hz, 6 H) 1.85 (q, *J*=7.3 Hz, 6 H) 1.33 (s, 18 H) 0.76 (t, *J*=7.4 Hz, 9 H); **^{13}C NMR** (101 MHz, DMSO d_6) δ ppm 181.28, 54.74, 52.52, 41.05, 32.02, 26.82, 8.28;

LR-MS (ESI$^+$) *m/z* 534.42 $[M + H]^+$, 556.39 $[M + Na]^+$

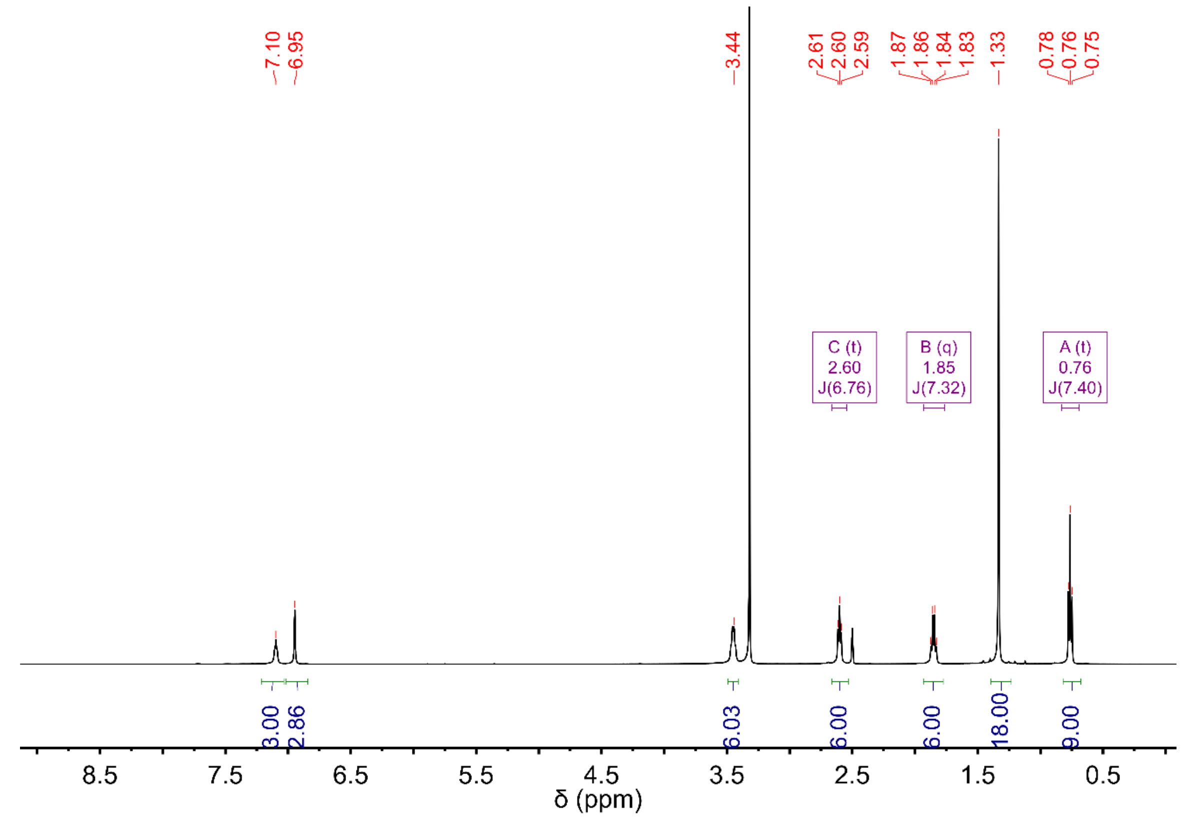

Figure 8. ^{1}H NMR (400 MHz) spectrum of 1,1',1"-(nitrilotris(ethane-2,1-diyl))tris(3-(tert-pentyl)thiourea) in $(CD_3)_2SO$ at 298 K.

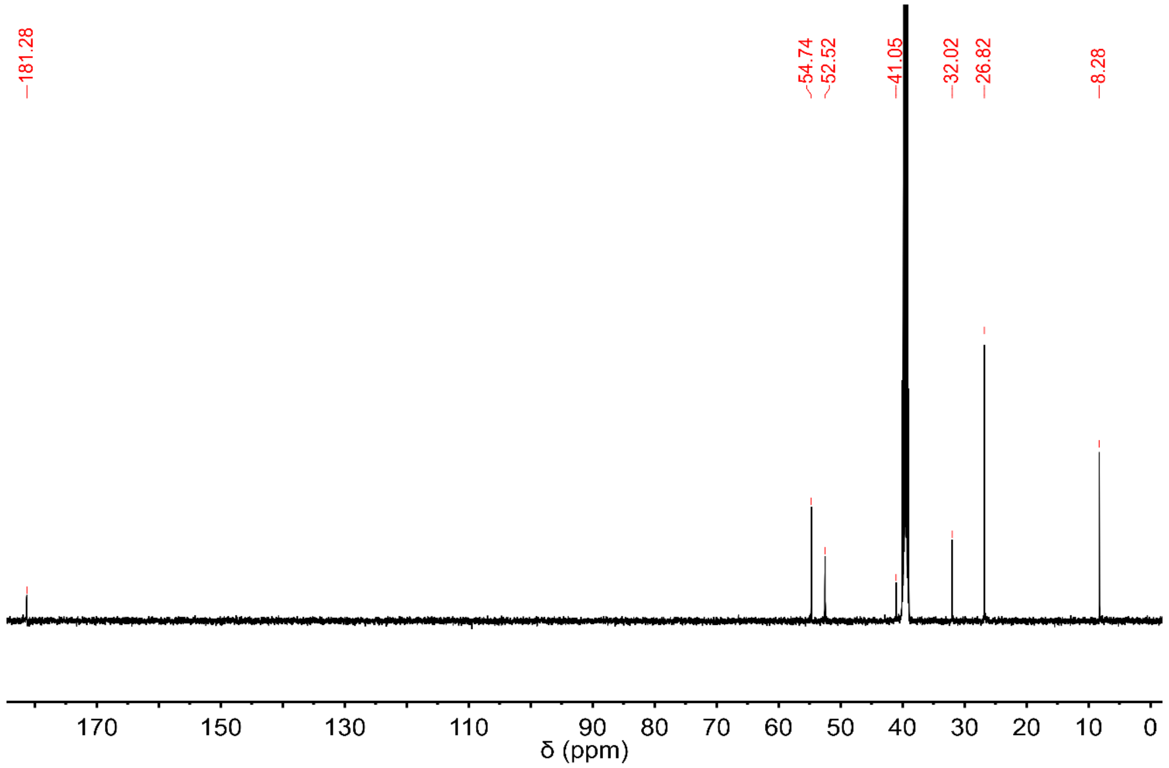

Figure 9. ^{13}C NMR (101 MHz) spectrum of 1,1',1"-(nitrilotris(ethane-2,1-diyl))tris(3-(tert-pentyl)thiourea) in $(CD_3)_2SO$ at 298 K.

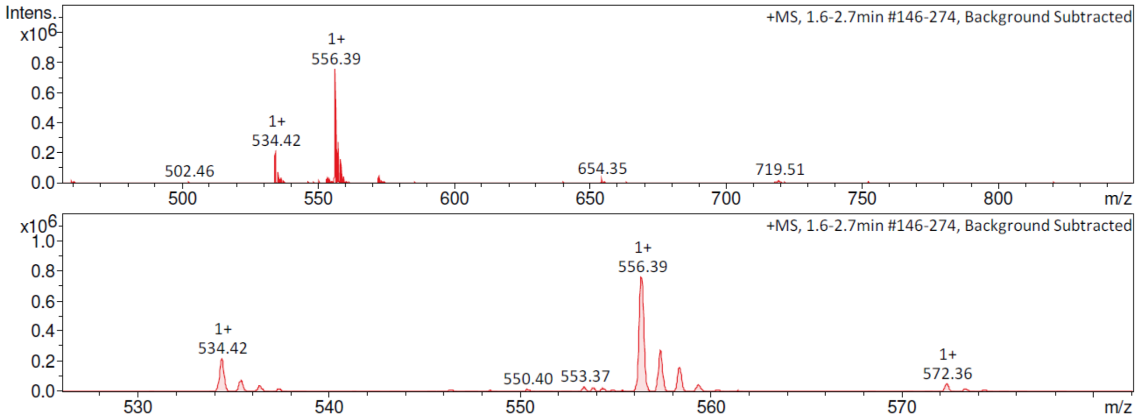

Figure 10. LR-MS (ESI$^+$) of 1,1',1''-(nitrilotris(ethane-2,1-diyl))tris(3-(tert-pentyl)thiourea).

1,1',1''-(Nitrilotris(ethane-2,1-diyl))tris(3-pentylthiourea)

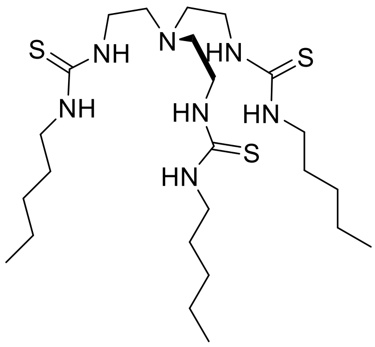

Tris-(2-aminoethyl)amine (0.74 mL, 5.0 mmol) was dissolved in DCM (50 mL) and 1-pentylisothiocyanate (2.1 mL, 15 mmol) was added dropwise. The solution was stirred at room temperature for 18 hr. Solvent was removed to leave a yellow oil which was immersed in hexane for 24 hr. After 24 hr a solid precipitate formed, this was filtered and washed with hexane to give a white powder (2.669 g, 100%).

^{1}H NMR (400 MHz, DMSO-d_6) δ ppm 7.46 (br s, 3 H) 7.16 (br s, 3 H) 3.44 (br s, 6 H) 3.32 (br s, 6 H) 2.61 (m, 6 H) 1.49-1.42 (m, 6 H) 1.30-1.22 (m, 12 H) 0.86 (t, *J*=6.9 Hz, 9 H); **^{13}C NMR** (101 MHz, DMSO-d_6) δ ppm 182.07, 52.62, 43.50, 41.54, 28.64, 28.45, 21.92, 13.92

LR-MS (ESI$^+$) *m/z* 534.38 $[M + H]^+$, 556.36 $[M + Na]^+$

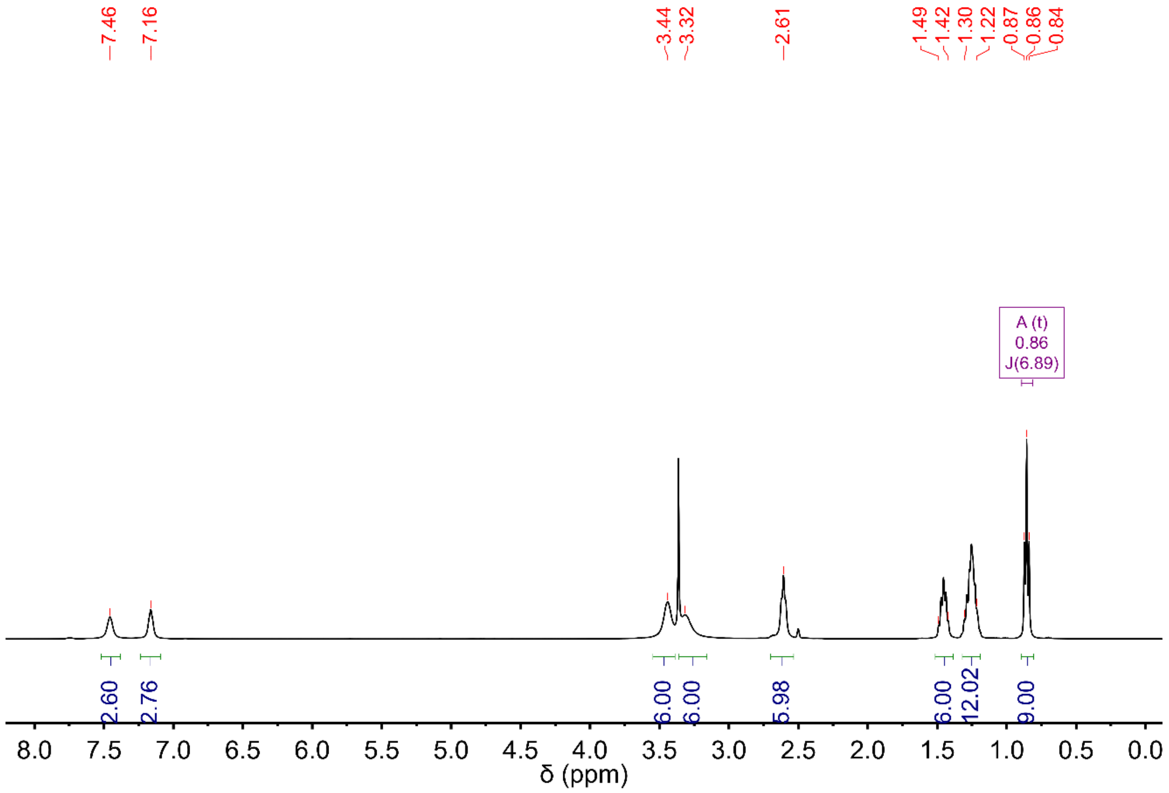

Figure 11. ^{1}H NMR (400 MHz) spectrum of 1,1',1''-(nitrilotris(ethane-2,1-diyl))tris(3-pentylthiourea) in $(CD_3)_2SO$ at 298 K.

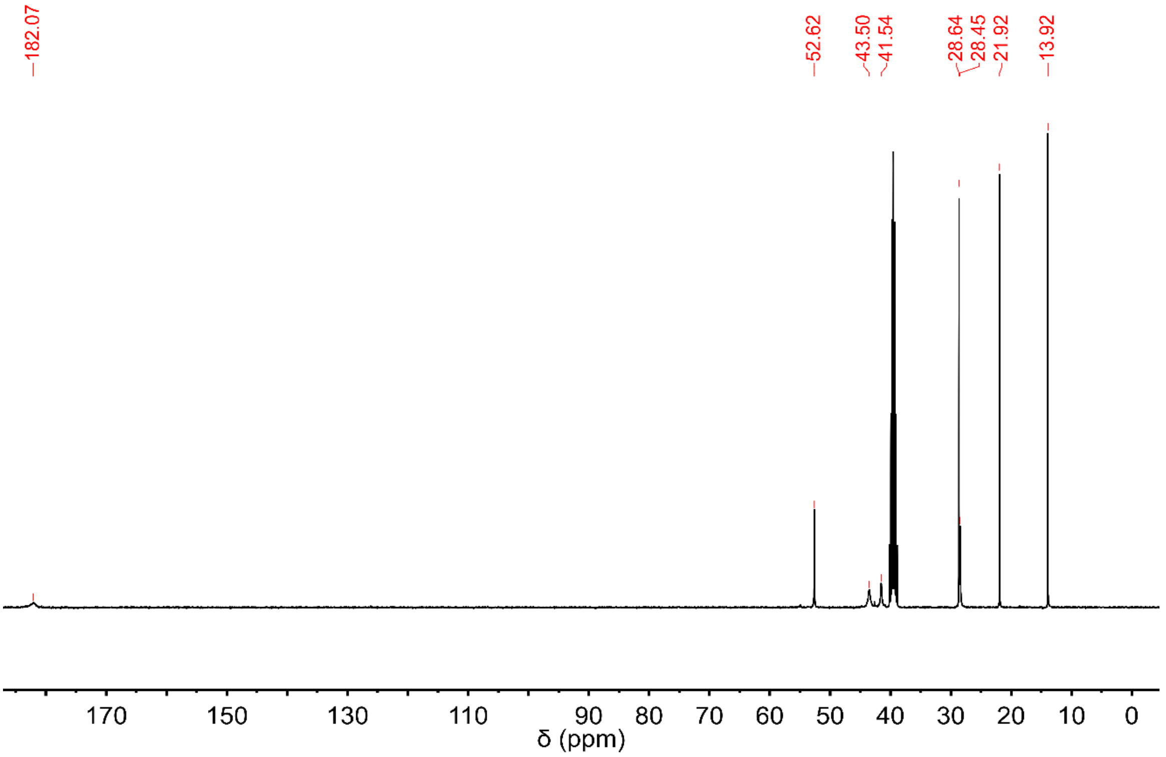

Figure 12. ^{13}C NMR (101 MHz) spectrum of 1,1',1''-(nitrilotris(ethane-2,1-diyl))tris(3-pentylthiourea) in $(CD_3)_2SO$ at 298 K.

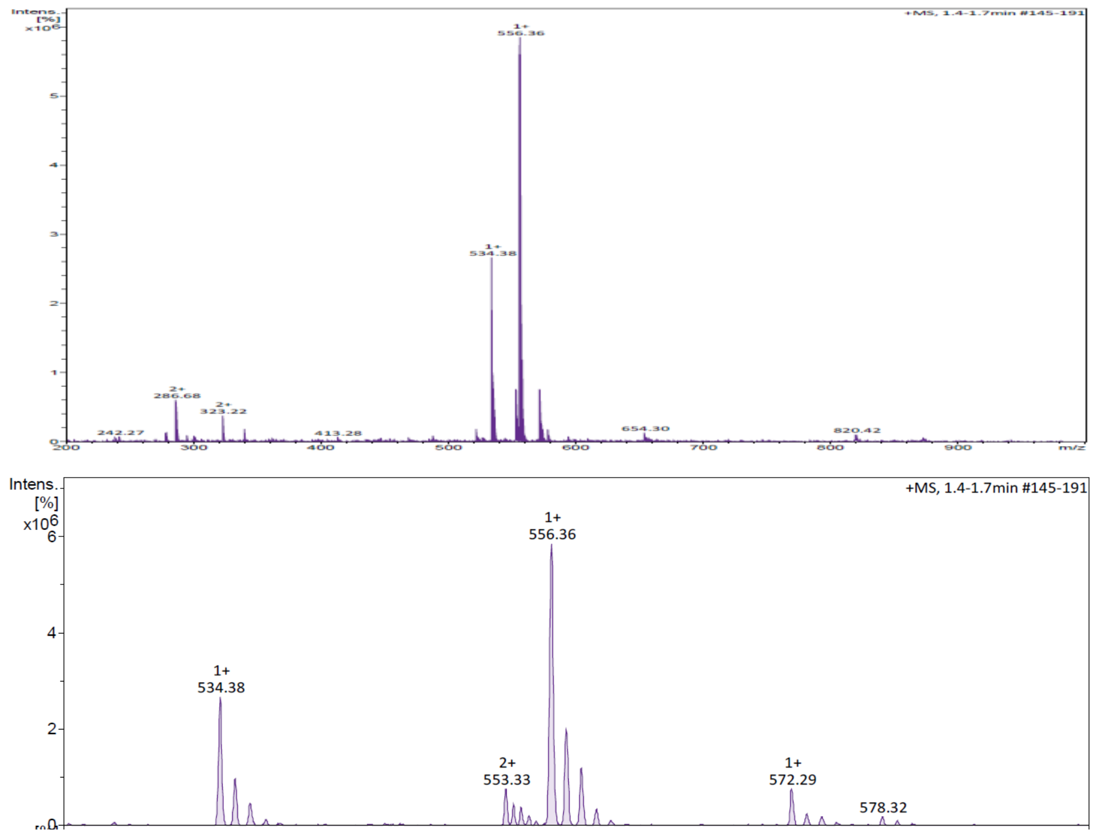

Figure 13. LR-MS (ESI$^+$) of 1,1',1''-(nitrilotris(ethane-2,1-diyl))tris(3-pentylthiourea).

tert-Butyl (2-(bis(2-aminoethyl)amino)ethyl)carbamate

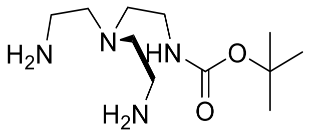

Tris-(2-aminoethyl)amine (0.5 mL, 3.34 mmol) was dissolved in anhydrous DCM (60 mL) and cooled to 0 °C. Di-tert-butyl dicarbonate (0.07 mL, 3.34 mmol) was dissolved in DCM (10 mL) and this solution was added dropwise to the ice cold solution over 2 hr. The solution was warmed to room temperature and stirred for 18 hr. Solvent was removed to leave a white residue which was dissolved in water. This was washed with $CHCl_3$ (5 x 30 mL), the organic phases were combined, dried over $MgSO_4$ and solvent removed to leave a clear oil (0.13 g, 95 %).

^{1}H NMR (400 MHz, $CDCl_3$-*d*) δ ppm 5.32 (br s, 1 H) 3.18-3.16 (m, 2 H) 2.74 (t, *J*=6.0 4 H) 2.53-2.43 (m, 6 H) 1.68 (br s, 4 H) 1.43 (s, 9 H); **^{13}C NMR** (101 MHz, $CDCL_3$-*d*) δ ppm 156.33, 79.19, 57.36, 54.16, 39.82, 38.94, 28.56;

LR-MS (ESI^+) *m/z* 247.22 $[M + H]^+$

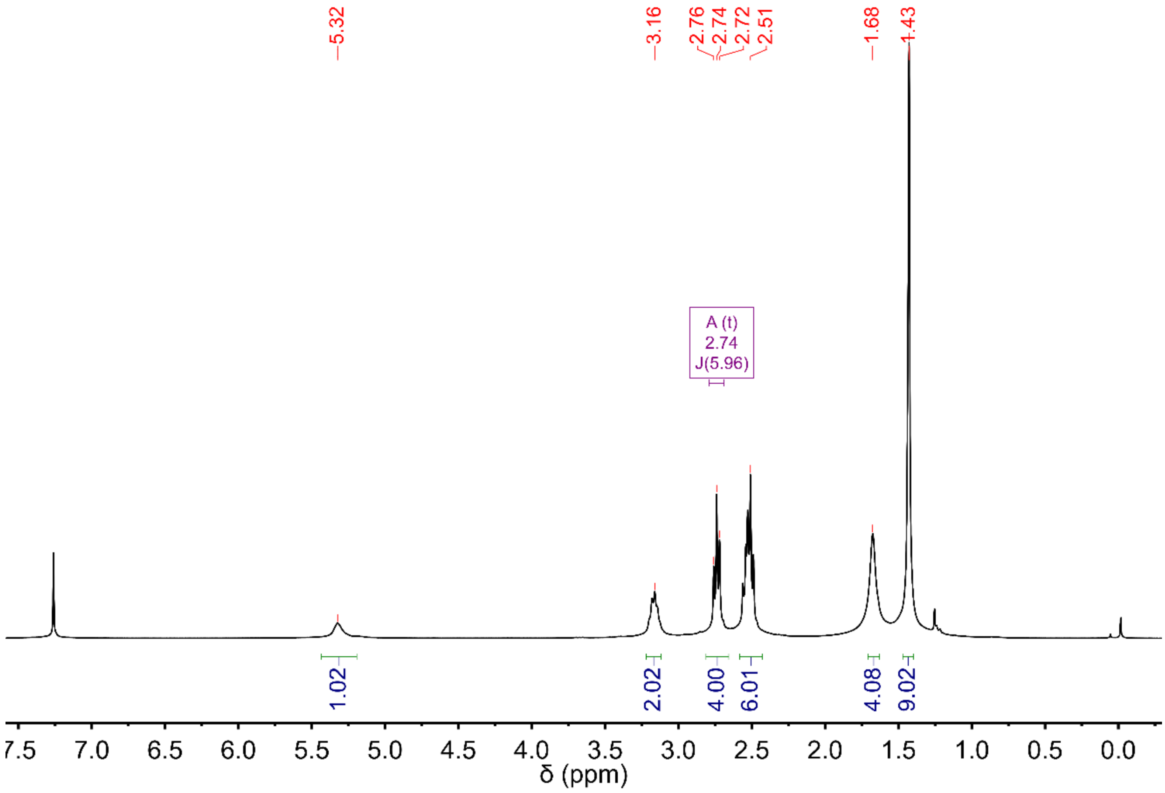

Figure 14. ^{1}H NMR (400 MHz) spectrum of *tert*-butyl (2-(bis(2-aminoethyl)amino)ethyl)carbamate in $CDCl_3$ at 298 K.

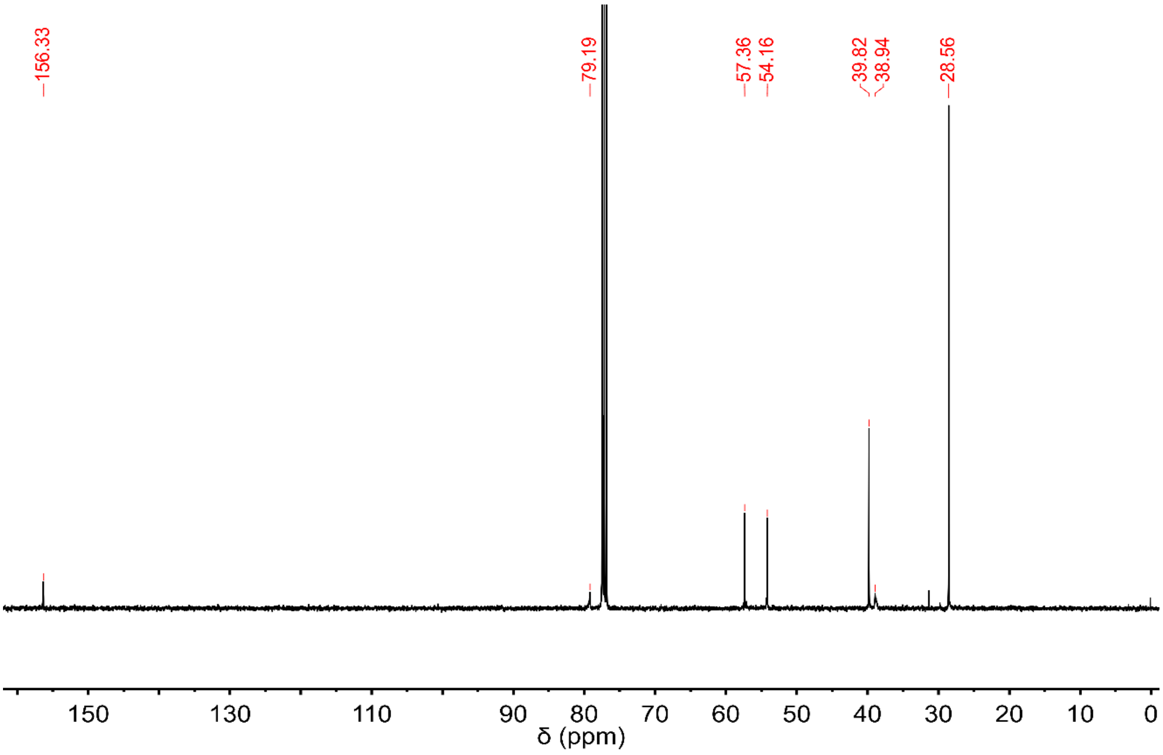

Figure 15. ^{13}C NMR (101 MHz) spectrum of *tert*-butyl (2-(bis(2-aminoethyl)amino)ethyl)carbamate in $CDCl_3$ at 298 K.

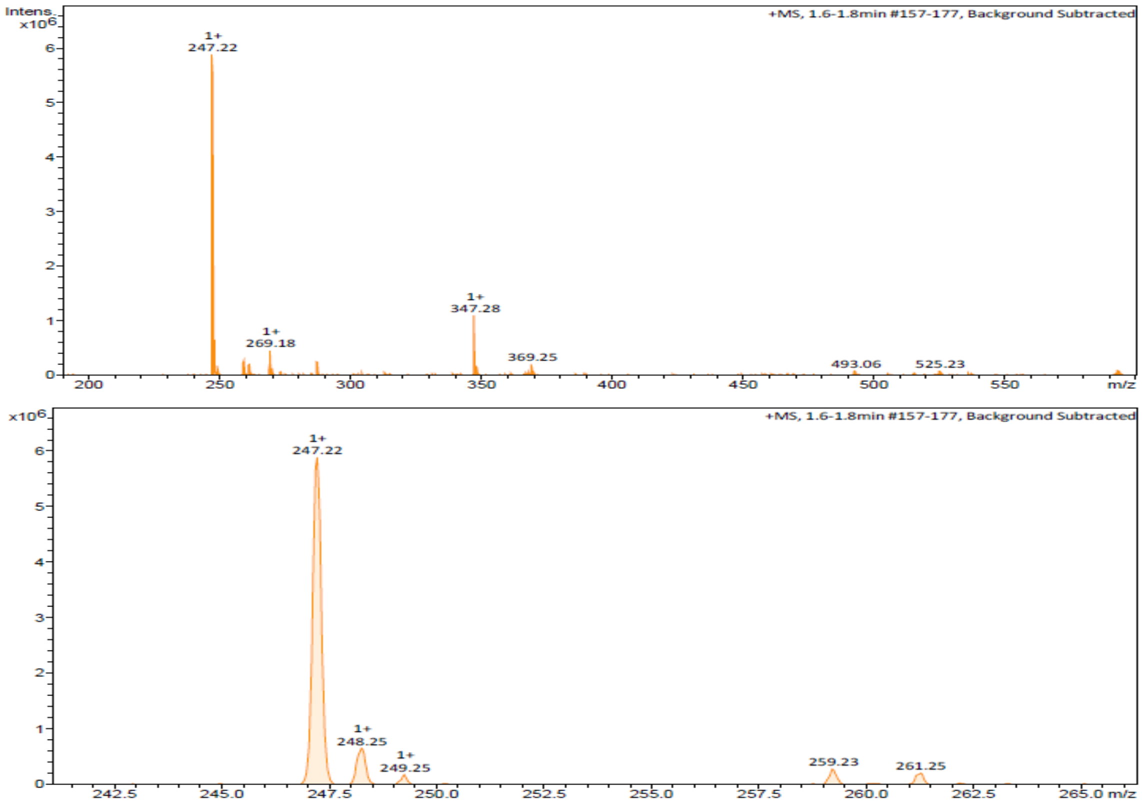

Figure 16. LR-MS (ESI$^+$) of *tert*-butyl (2-(bis(2-aminoethyl)amino)ethyl)carbamate.

tert-Butyl (2-(bis(2-isothiocyanatoethyl)amino)ethyl)carbamate

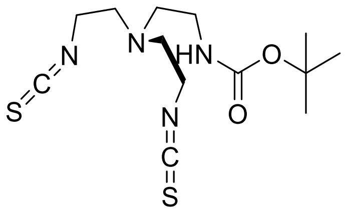

tert-Butyl (2-(bis(2-aminoethyl)amino)ethyl)carbamate (1.90 g, 7.71 mmol) was dissolved in anhydrous THF (50 mL). Carbon disulphide (9.3 mL, 154 mmol) was added to the solution and the colour changed to yellow. N,N'-dicyclohexylcarbodiimide (3.18 g, 15.4 mmol) was dissolved in anhydrous THF (10 mL) and this was added to the yellow solution dropwise over 30 mins. The resulting solution was left to stir for 18 hr at room temperature. Solvent was removed to leave a yellow residue and the DCC thiourea by-product was recrystallised using DCM three times. The yellow oil was then further purified by column chromatography (silica, DCM → 2% MeOH) to give a yellow oil (1.40 g, 55 %). Remaining DCC by-product does not affect the next reaction.

^{1}H NMR (400 MHz, $CDCl_3$-*d*) δ ppm 5.06 (br s, 1 H) 3.55 (t, *J*=6.1 Hz, 4 H) 3.21-3.15 (m, 2 H) 2.87 (t, *J*=6.1 Hz, 4 H) 2.70 (t, *J*=6.0 Hz, 2 H) 1.45 (s, 9 H); **^{13}C NMR** (101 MHz, $CDCl_3$-*d*) δ ppm 156.26, 133.14, 79.52, 54.49, 53.98, 44.12, 38.89, 28.53;

LR-MS (ESI^+) *m/z* 331.13 $[M + H]^+$, 353.12 $[M + Na]^+$

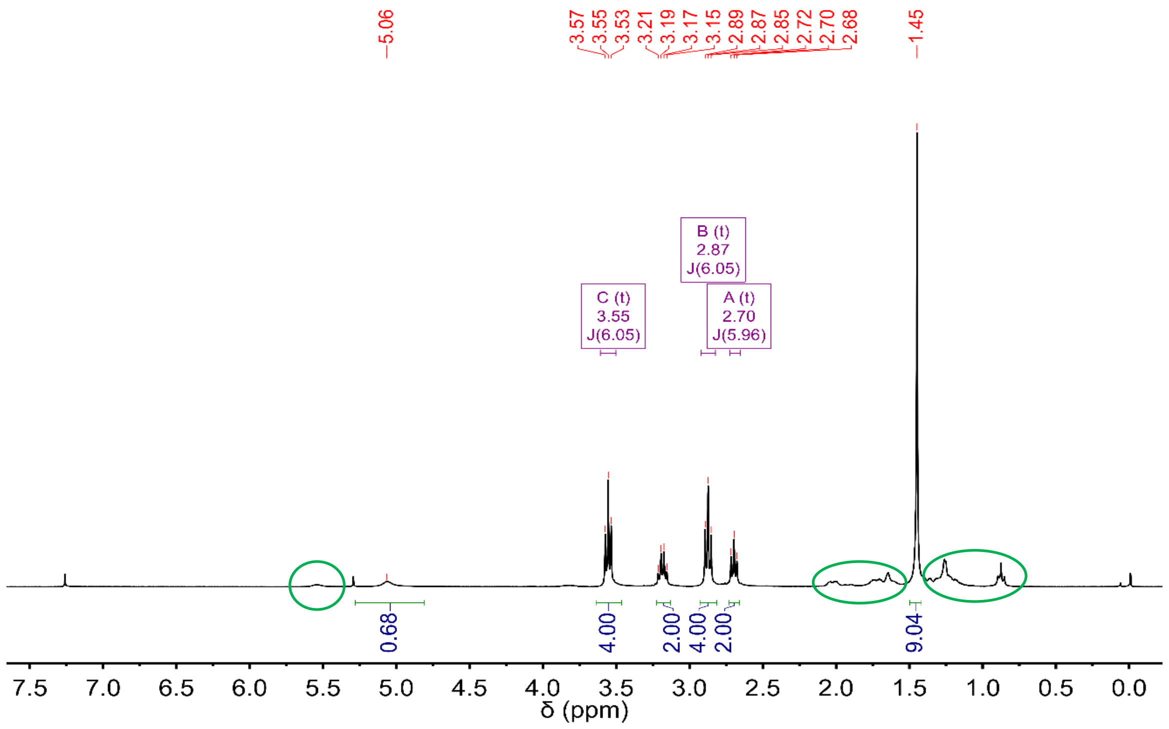

Figure 17. ^{1}H NMR (400 MHz) spectrum of *tert*-butyl (2-(bis(2-isothiocyanatoethyl)amino)ethyl)carbamate in $CDCl_3$ at 298 K. There is evidence of the DCC thiourea by product from the peaks present between 1-2 ppm and 5.5 ppm highlighted in green.

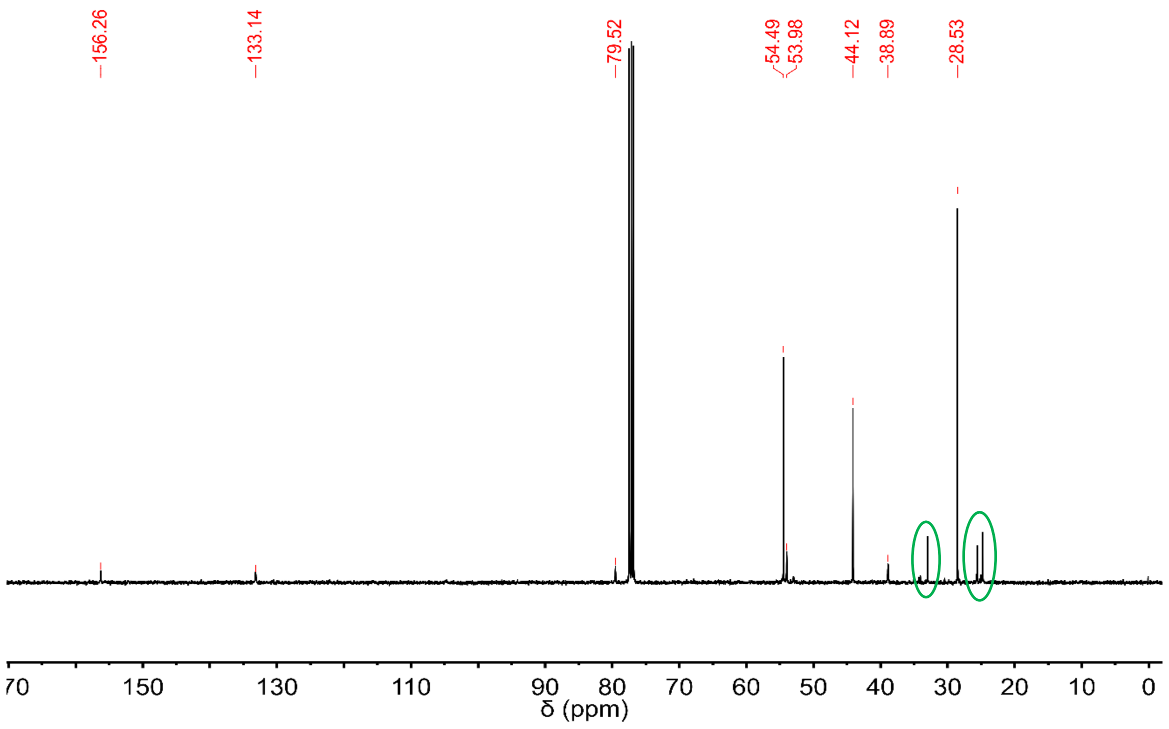

Figure 18. ^{13}C NMR (101 MHz) spectrum of *tert*-butyl (2-(bis(2-isothiocyanatoethyl)amino)ethyl)carbamate in $CDCl_3$ at 298 K. There is evidence of the DCC thiourea by product from the peaks present between 25-30 ppm and 30-35 ppm highlighted in green.

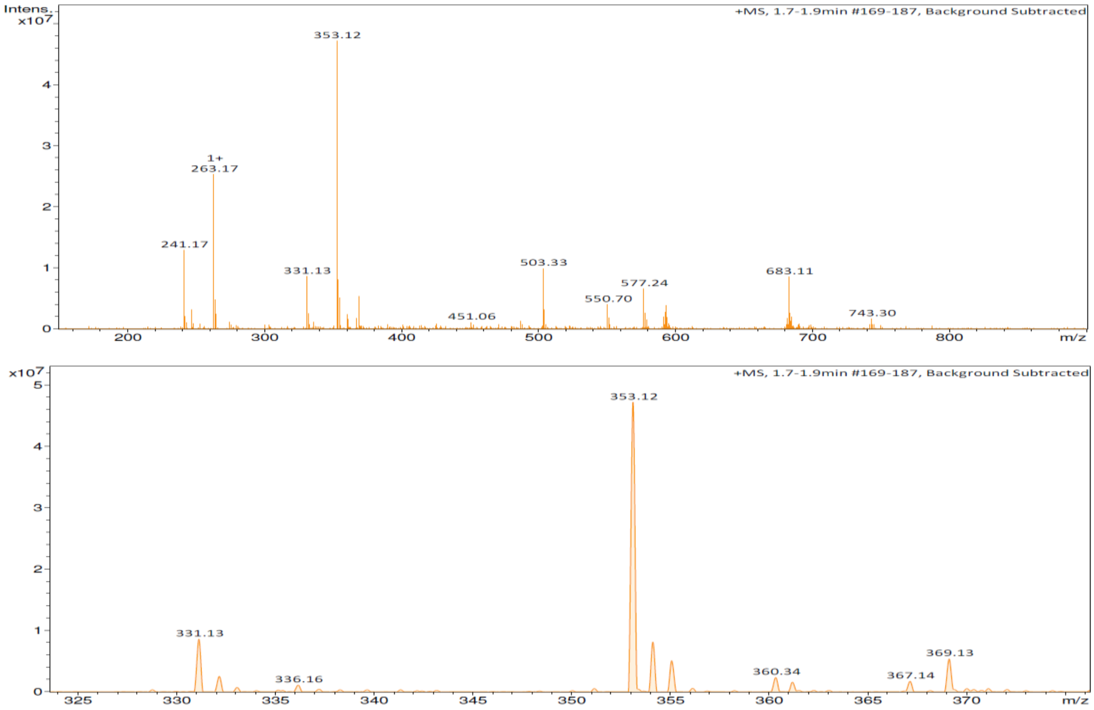

Figure 19. LR-MS (ESI$^+$) of *tert*-butyl (2-(bis(2-isothiocyanatoethyl)amino)ethyl)carbamate. There is evidence of the thiourea by-product from the peaks at *m/z* 241.17 [M + H]$^+$, 263.17 [M + Na]$^+$.

tert-Butyl (2-(bis(2-(3-pentylthioureido)ethyl)amino)ethyl)carbamate

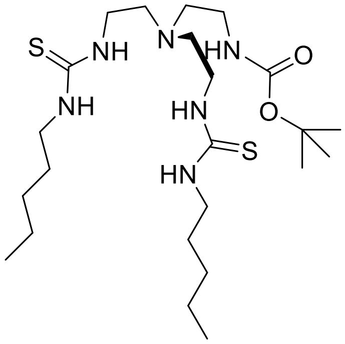

tert-Butyl (2-(bis(2-isothiocyanatoethyl)amino)ethyl)carbamate (200 mg, 0.61 mmol) was dissolved in anhydrous DCM (10 mL). 1-pentylamine (0.12 mL, 1.04 mmol) was added dropwise. The mixture was stirred for 18 hr at room temperature. After 18 hr solvent was removed and the crude product was purified by column chromatography (silica, DCM → 2% MeOH) to give a white crystalline solid (0.169 g, 55 %).

^{1}H NMR (400 MHz, DMSO-*d*$_6$) δ ppm 7.47 (br s, 2 H) 7.18 (br s, 2 H) 6.68 (t, *J*=4.9 Hz, 1 H) 3.40 (br s, 4 H) 3.28 (br s, 4 H) 2.98-2.97 (m, 2 H), 2.56 (t, *J*=6.4 Hz, 4H) 2.48 (br s, 2 H) 1.48-1.42 (m, 4 H) 1.37 (s, 9 H) 1.32-1.24 (m, 8 H) 0.86 (t, *J*=6.9 Hz, 6 H); **^{13}C NMR** (101 MHz, DMSO-*d*$_6$) δ ppm 182.20, 155.73, 77.64, 53.62, 52.81, 43.52, 41.64, 38.31, 28.63, 28.46, 28.25, 21.92, 13.93

LR-MS (ESI$^+$) *m/z* 505.38 [M + H]$^+$, 527.36 [M + Na]$^+$; **HR-MS** (ESI$^+$) calcd for $C_{23}H_{48}N_6O_2S_2$ [M+H]$^+$: 505.33529, found *m/z* 505.33561

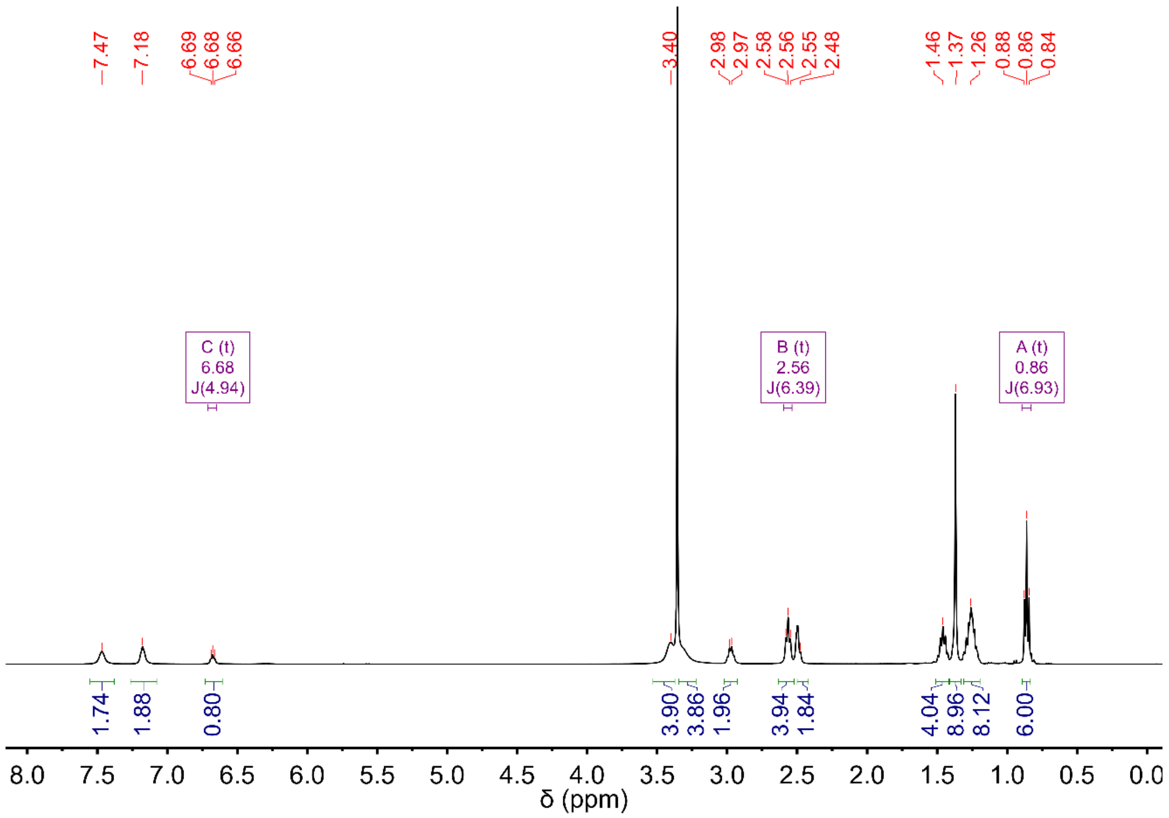

Figure 20. ^{1}H NMR (400 MHz) spectrum of *tert*-butyl (2-(bis(2-(3-pentylthioureido)ethyl)amino)ethyl)carbamate in $(CD_3)_2SO$ at 298 K.

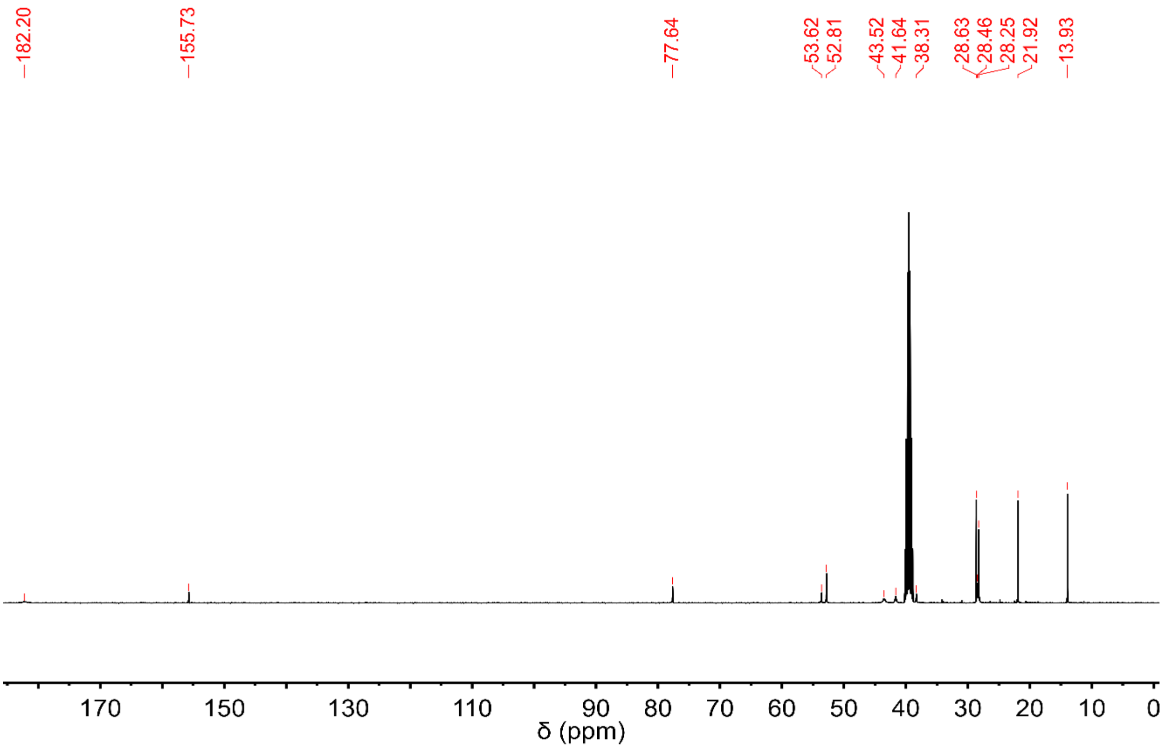

Figure 21. ^{13}C NMR (101 MHz) spectrum of *tert*-butyl (2-(bis(2-(3-pentylthioureido)ethyl)amino)ethyl)carbamate in $(CD_3)_2SO$ at 298 K.

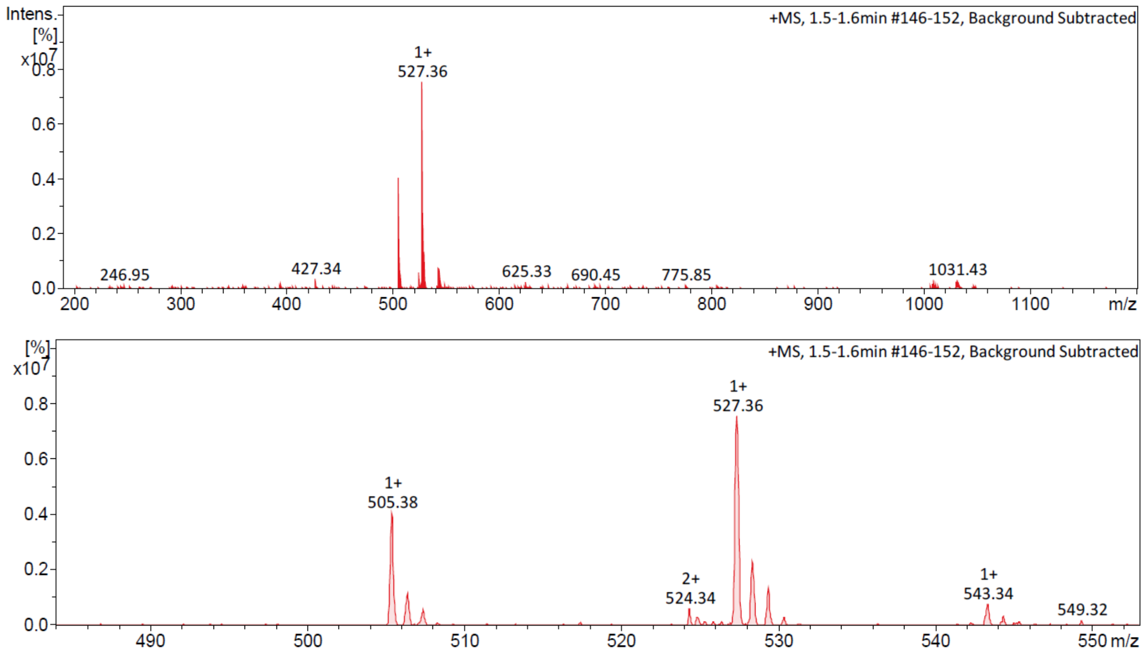

Figure 22. LR-MS (ESI$^+$) of *tert*-butyl (2-(bis(2-(3-pentylthioureido)ethyl)amino)ethyl)carbamate.

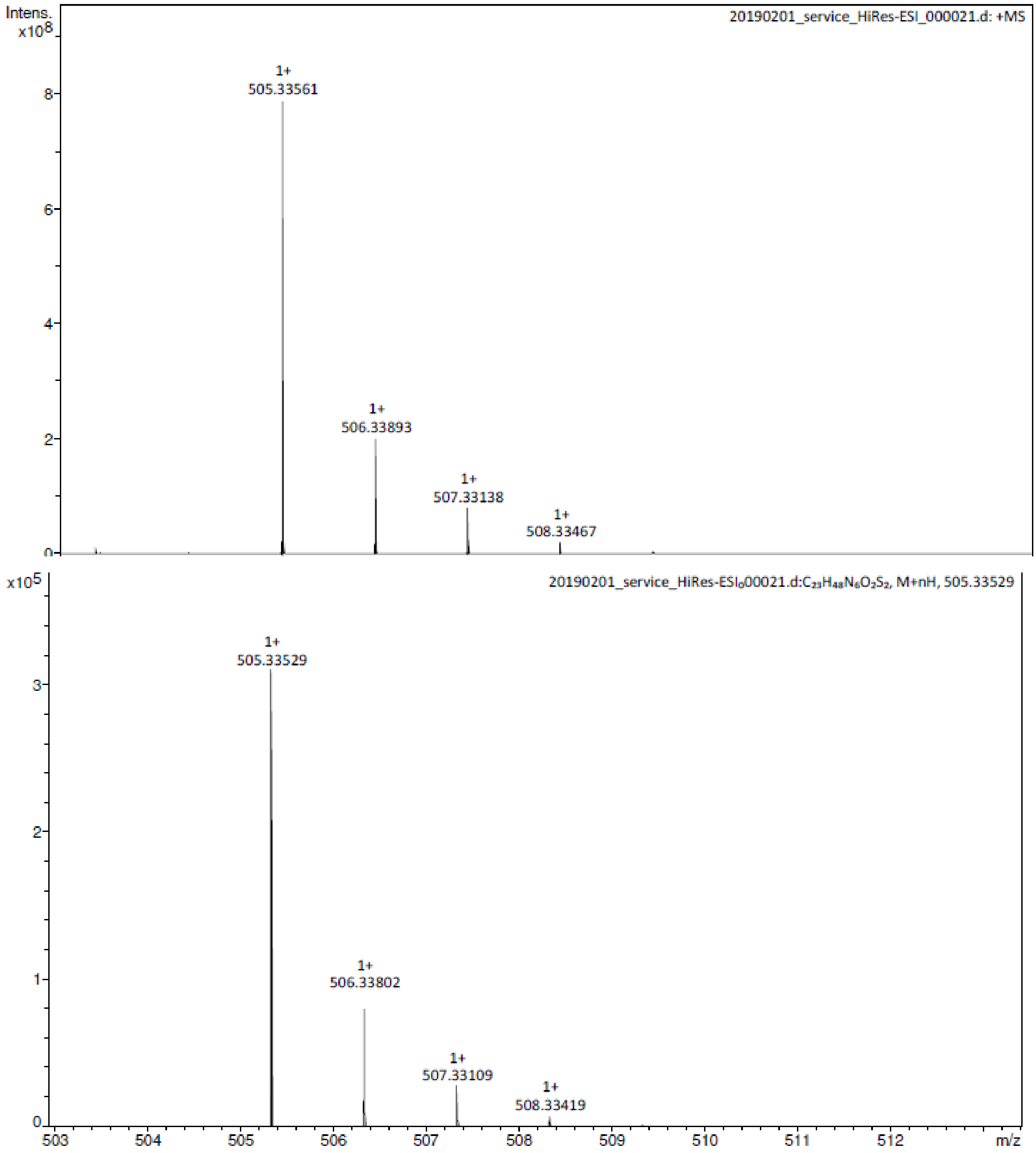

Figure 23. HR-MS (ESI$^+$) of *tert*-butyl (2-(bis(2-(3-pentylthioureido)ethyl)amino)ethyl)carbamate.

1,1'-(((2-(3-(*tert*-Pentyl)thioureido)ethyl)azanediyl)bis(ethane-2,1-diyl))bis(3-pentylthiourea)

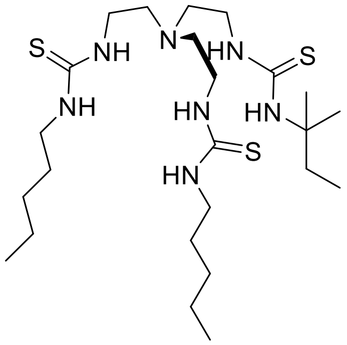

tert-Butyl (2-(bis(2-(3-pentylthioureido)ethyl)amino)ethyl)carbamate (100 mg, 0.20 mmol) was dissolved in $CHCl_3$. TFA (1 mL) was added dropwise and the mixture was stirred at room temperature for 1.5 hr. Solvent was removed and the residue was washed with toluene (3 x 5 mL) The glass-like solid was immediately dissolved in anhydrous DMF (4 mL) and TEA (0.97 mL, 6.93 mmol) was added dropwise. 2-Isothiocyanato-2-methylbutane (0.045 g, 0.35 mmol) was added to the reaction mixture dropwise and the solution was stirred at room temperature for 18 hr. After 18 hr solvent was removed and the crude product was purified by column chromatography (silica, DCM → 2% MeOH) to give a white crystalline solid (0.169 g, 55 %).

^{1}**H NMR** (400 MHz, DMSO-*d*$_6$) δ ppm 7.48 (br s, 2 H) 7.20 (br s, 2 H) 7.11 (br s, 1 H) 6.98 (s, 1 H) 3.45 (br s, 6 H) 3.31 (br s, 4 H) 2.65-2.58 (m, 6 H), 1.85 (q, *J*=7.3 Hz, 2H) 1.49-1.42 (m, 4 H) 1.34 (s, 6 H) 1.32-1.24 (m, 8 H) 0.87 (t, *J*=7.0 Hz, 6 H) 0.76 (t, *J*=7.4 Hz, 3 H); ^{13}**C NMR** (101 MHz, DMSO-*d*$_6$) δ ppm 181.30, 54.92, 54.79, 52.68, 52.56, 43.54, 41.54, 41.16, 32.06, 28.65, 28.46, 26.84, 21.93, 13.95, 8.31

LR-MS (ESI$^+$) *m/z* 534.40 $[M + H]^+$, 556.36 $[M + Na]^+$; **HR-MS** (ESI$^+$) calcd for $C_{24}H_{51}N_7S_3$ $[M+H]^+$: 556.32603, found *m/z* 556.32630

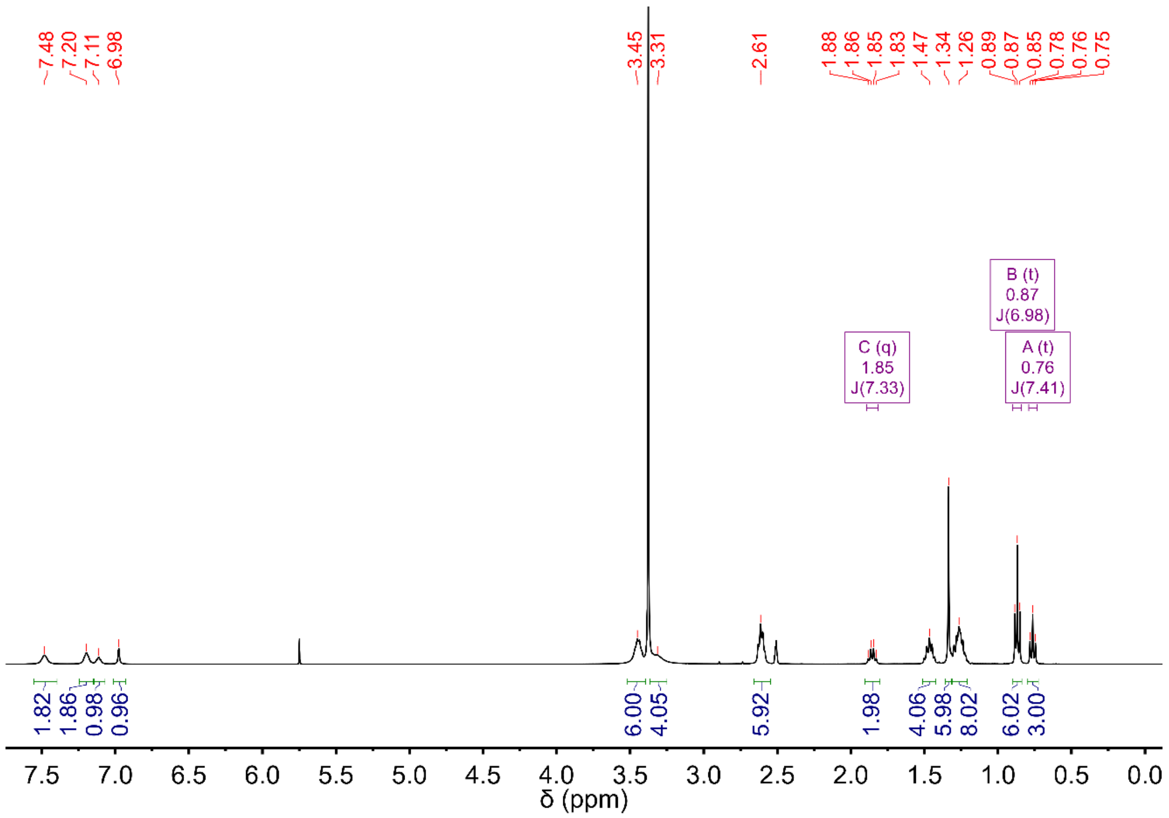

Figure 24. ^{1}H NMR (400 MHz) spectrum of 1,1'-(((2-(3-(*tert*-pentyl)thioureido)ethyl)azanediyl)bis(ethane-2,1-diyl))bis(3-pentylthiourea) in $(CD_3)_2SO$ at 298 K.

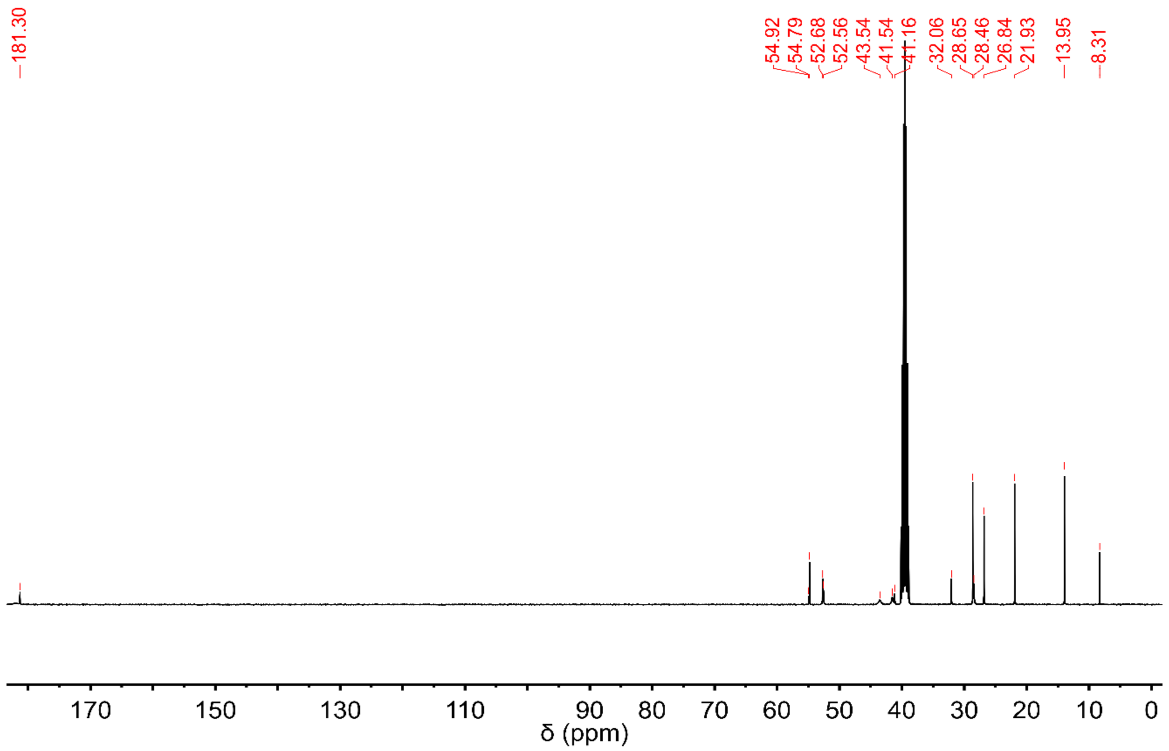

Figure 25. ^{13}C NMR (101 MHz) spectrum of 1,1'-(((2-(3-(*tert*-pentyl)thioureido)ethyl)azanediyl)bis(ethane-2,1-diyl))bis(3-pentylthiourea) in $(CD_3)_2SO$ at 298 K.

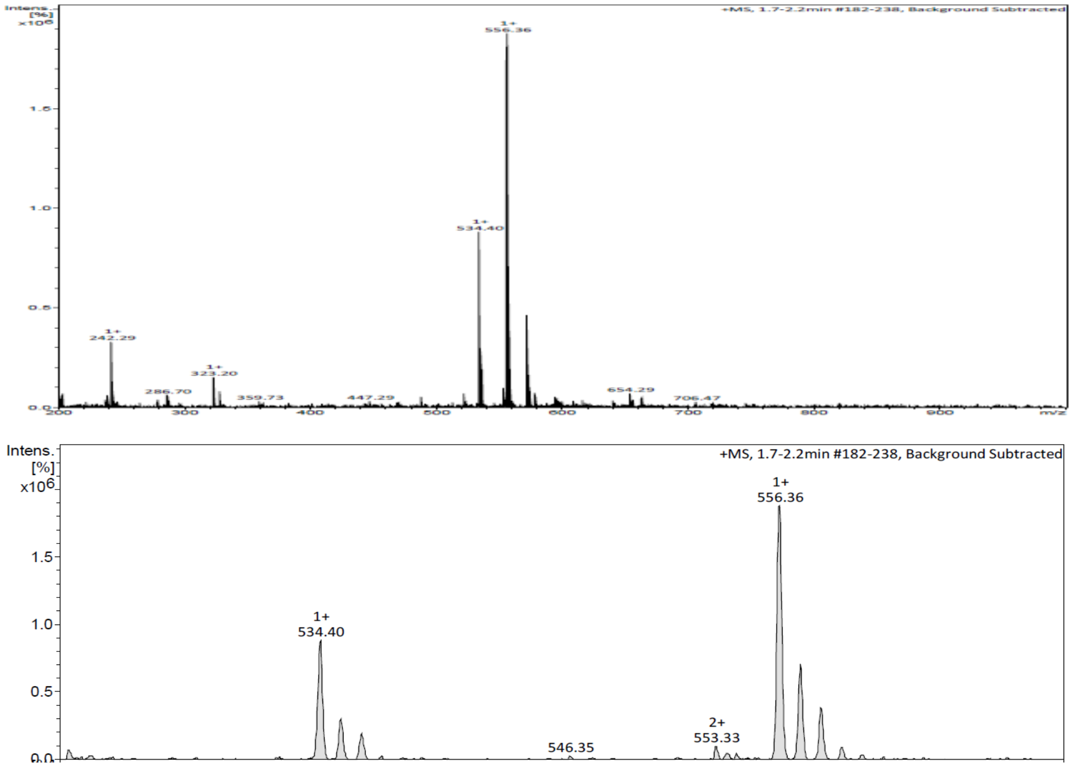

Figure 26. LR-MS (ESI$^+$) of 1,1'-(((2-(3-(*tert*-pentyl)thioureido)ethyl)azanediyl)bis(ethane-2,1-diyl))bis(3-pentylthiourea).

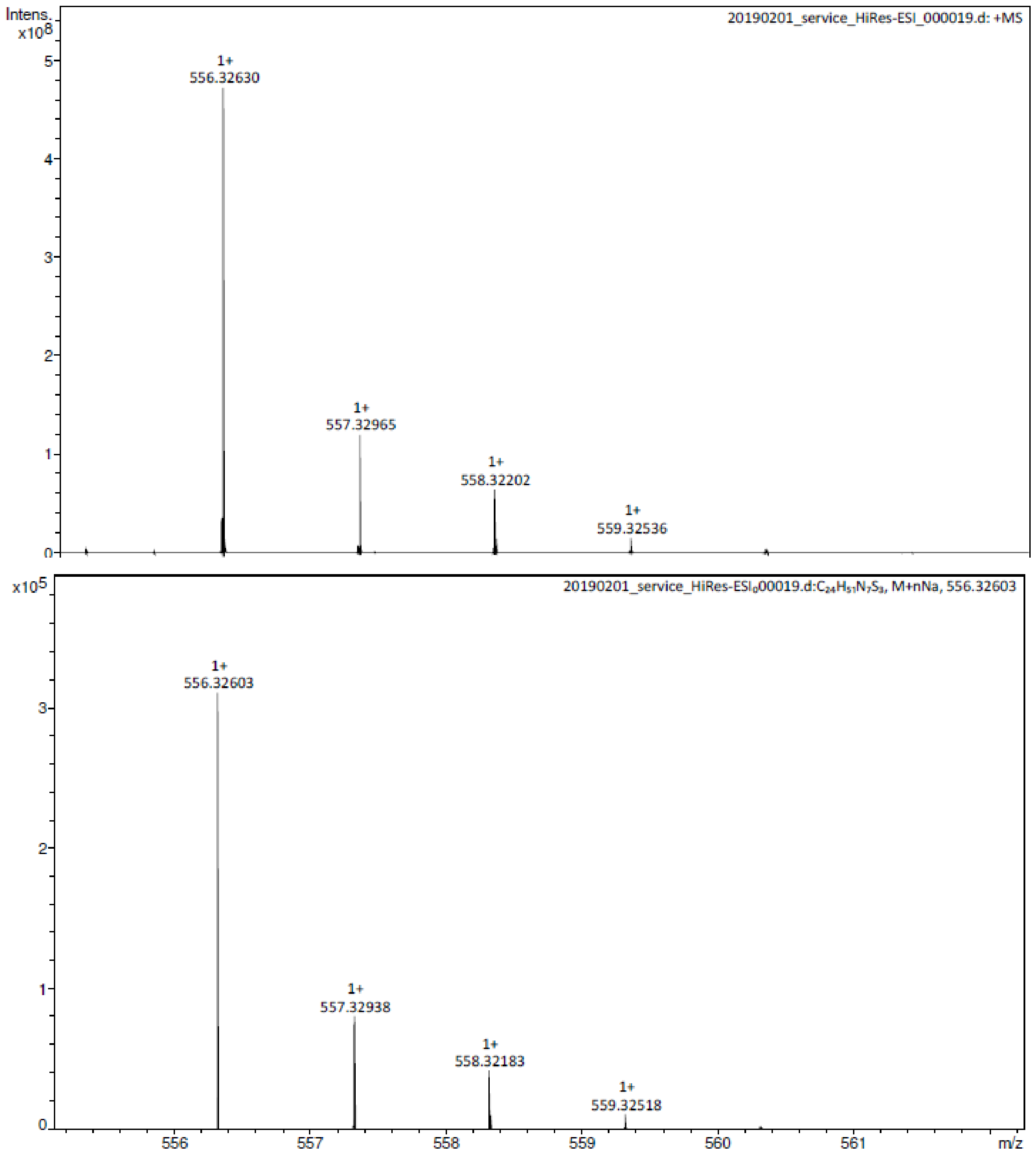

Figure 27. HR-MS (ESI$^+$) of 1,1'-(((2-(3-(*tert*-pentyl)thioureido)ethyl)azanediyl)bis(ethane-2,1-diyl))bis(3-pentylthiourea).

tert-Butyl (2-(bis(2-(3-(*tert*-pentyl)thioureido)ethyl)amino)ethyl)carbamate

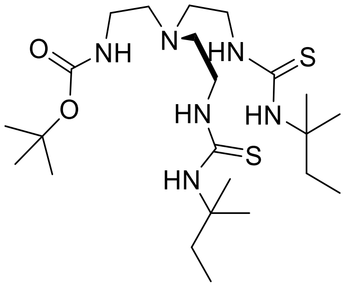

tert-Butyl (2-(bis(2-isothiocyanatoethyl)amino)ethyl)carbamate (450 mg, 1.36 mmol) was dissolved in anhydrous DCM (25 mL). 2-Methyl-2-butanamine (0.3 mL, 2.57 mmol) was added dropwise. The mixture was stirred for 18 hr at room temperature. After 18 hr solvent was removed and the crude product was purified by column chromatography (silica, DCM → 2% MeOH) to give a white crystalline solid (0.441 g, 64 %).

^{1}H NMR (400 MHz, DMSO-d_6) δ ppm 7.11 (br s, 2 H) 6.96 (s, 2 H) 6.66 (t, *J*=5.4 Hz, 1 H) 3.42-3.40 (m, 4 H) 2.99-2.97 (m, 2 H), 2.55 (t, *J*=6.6 Hz, 4 H) 2.48 (br s, 2 H) 1.85 (q, *J*=7.3 Hz, 4 H) 1.37 (s, 9 H) 1.33 (s, 12 H) 0.76 (t, *J*=7.4 Hz, 6 H); **^{13}C NMR** (101 MHz, DMSO-d_6) δ ppm 181.30, 155.71, 77.63, 54.73, 53.70, 52.74, 41.49, 38.25, 32.03, 28.26, 26.85, 8.29

LR-MS (ESI$^+$) *m/z* 505.38 $[M + H]^+$, 527.36 $[M + Na]^+$; **HR-MS** (ESI$^+$) calcd for $C_{23}H_{48}N_6O_2S_2$ $[M+H]^+$:505.33529, found *m/z* 505.33579

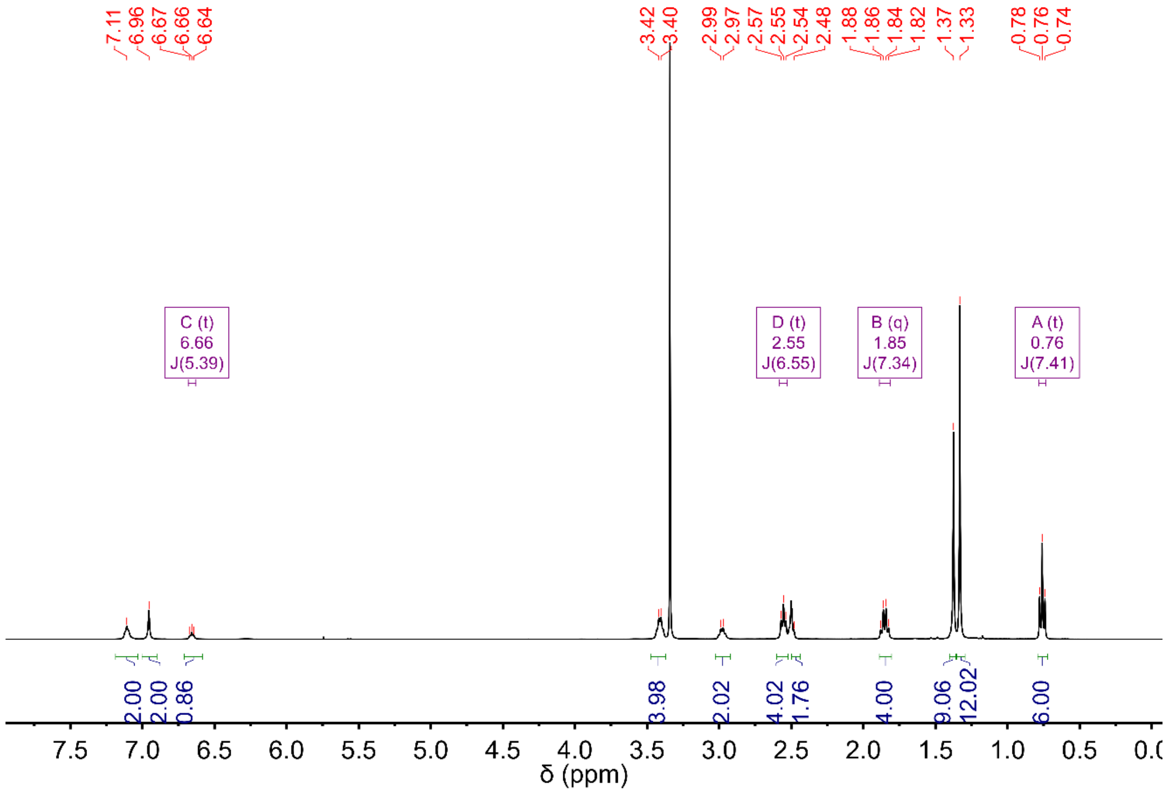

Figure 28. ^{1}H NMR (400 MHz) spectrum of *tert*-butyl (2-(bis(2-(3-(*tert*-pentyl)thioureido)ethyl)amino)ethyl)carbamate in $(CD_3)_2SO$ at 298 K.

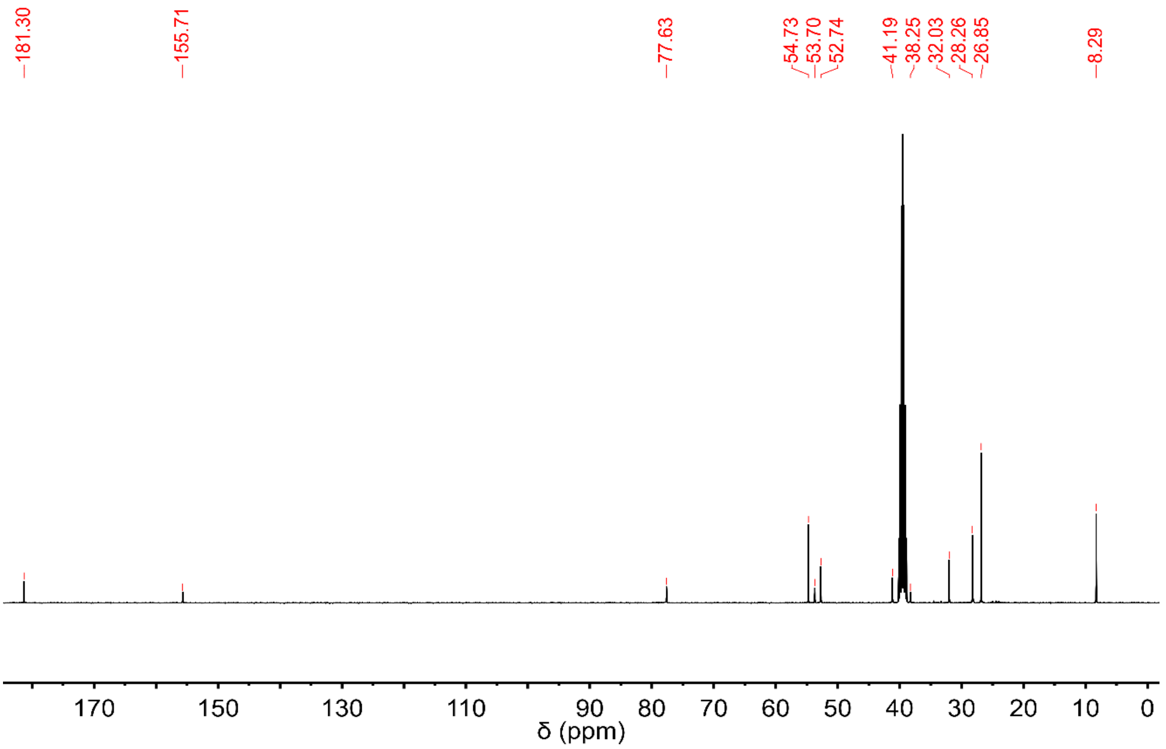

Figure 29. ^{13}C NMR (101 MHz) spectrum of *tert*-butyl (2-(bis(2-(3-(*tert*-pentyl)thioureido)ethyl)amino)ethyl)carbamate in $(CD_3)_2SO$ at 298 K.

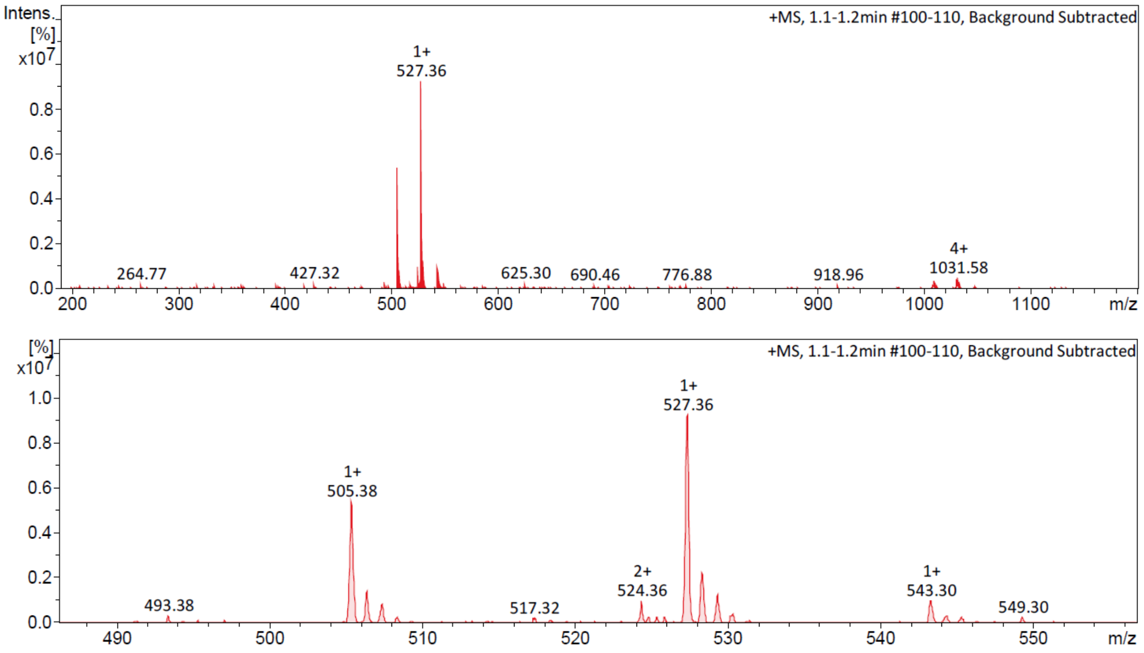

Figure 30. LR-MS (ESI$^+$) of *tert*-butyl (2-(bis(2-aminoethyl)amino)ethyl)carbamate.

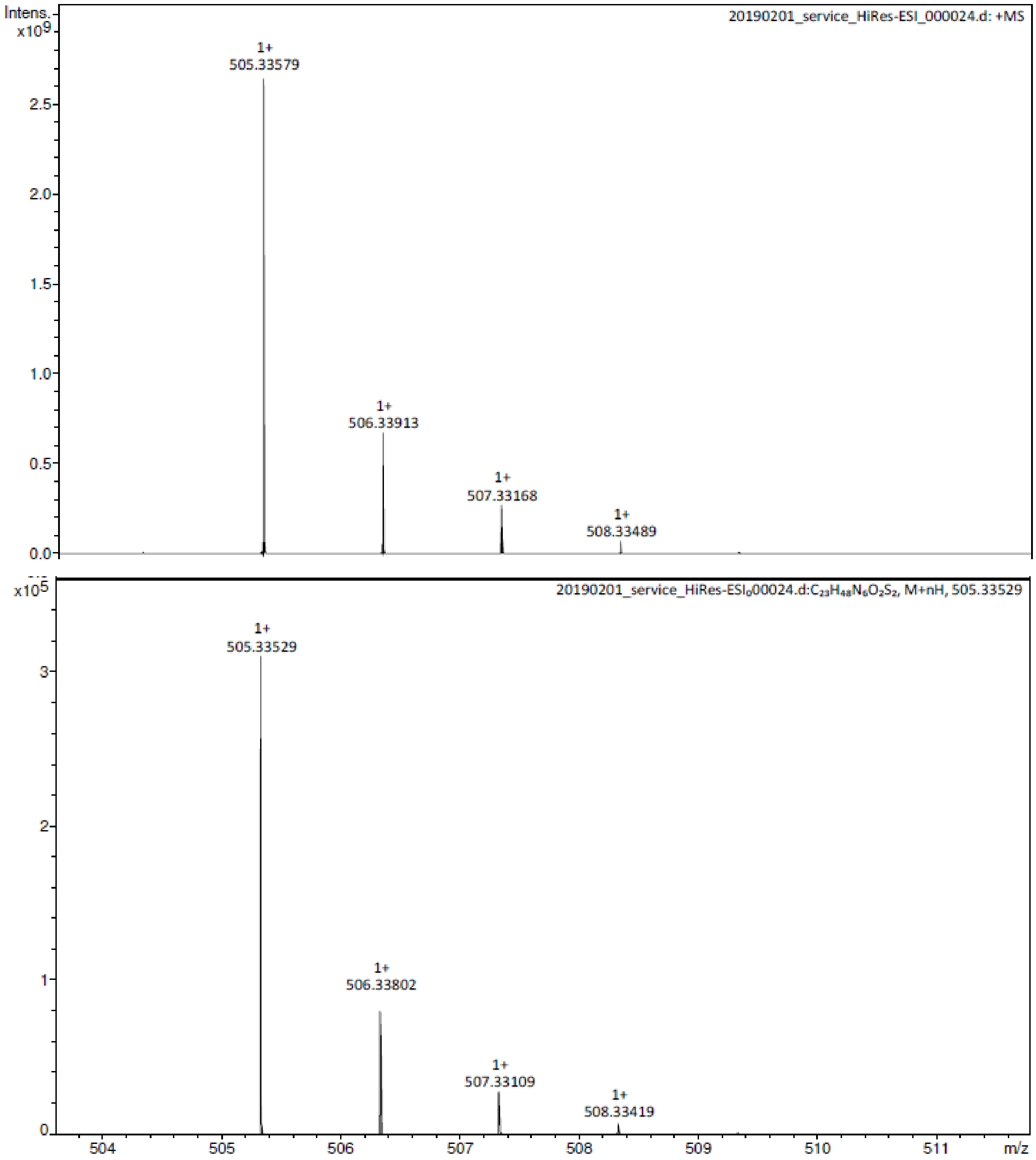

Figure 31. HR-MS (ESI$^+$) of *tert*-butyl (2-(bis(2-aminoethyl)amino)ethyl)carbamate.

1,1'-(((2-Aminoethyl)azanediyl)bis(ethane-2,1-diyl))bis(3-(*tert*-pentyl)thiourea)

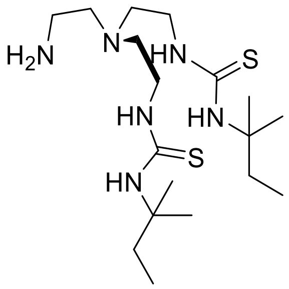

Tris-(2-aminoethyl)amine (0.18 mL, 1.23 mmol) was dissolved in DCM (5 mL). 2-Isothiocyanato-2-methylbutane (0.33 g, 2.55 mmol) was dissolved in DCM (1 mL) and added dropwise to the initial solution. The mixture was stirred for 5.5 hr at room temperature. After 5.5 hr solvent was removed and the crude product was purified by column chromatography (silica, DCM → 9% MeOH 1% NH_4OH) to give a clear oil (0.208 g, 42 %).

^{1}H NMR (400 MHz, DMSO-d_6) δ ppm 7.14 (br s, 2 H) 7.00 (s, 2 H) 3.43-3.42 (m, 6 H) 2.59-2.47 (m, 6 H), 1.85 (q, *J*=7.3 Hz, 4 H) 1.33 (s, 12 H) 0.76 (t, *J*=7.4 Hz, 6 H); **^{13}C NMR** (101 MHz, DMSO-d_6) δ ppm 181.30, 57.14, 54.74, 52.92, 41.18, 32.03, 28.26, 8.29

LR-MS (ESI$^+$) *m/z* 405.30 [M + H]$^+$, 427.28 [M + Na]$^+$; **HR-MS** (ESI$^+$) calcd for $C_{18}H_{40}N_6S_3$ [M+H]$^+$: 405.28286, found *m/z* 405.28286

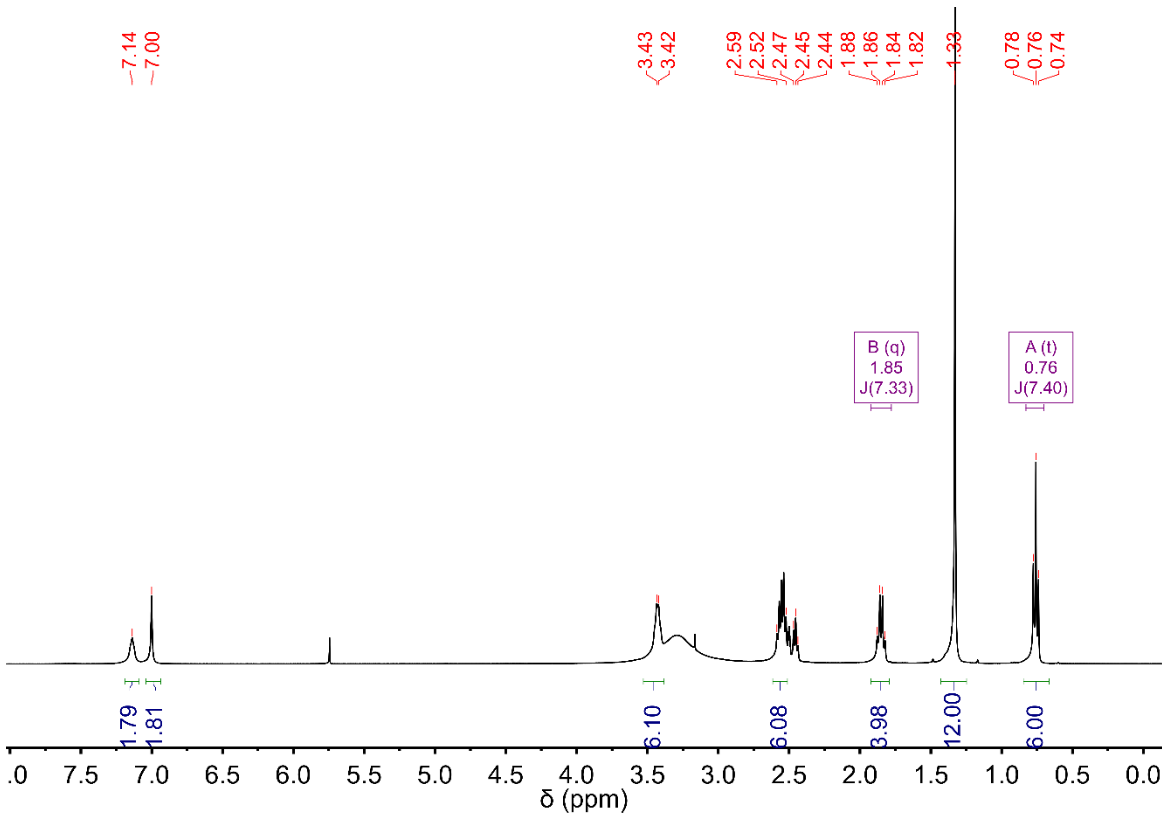

Figure 32. ^{1}H NMR (400 MHz) spectrum of 1,1'-(((2-aminoethyl)azanediyl)bis(ethane-2,1-diyl))bis(3-(*tert*-pentyl)thiourea) in $(CD_3)_2SO$ at 298 K.

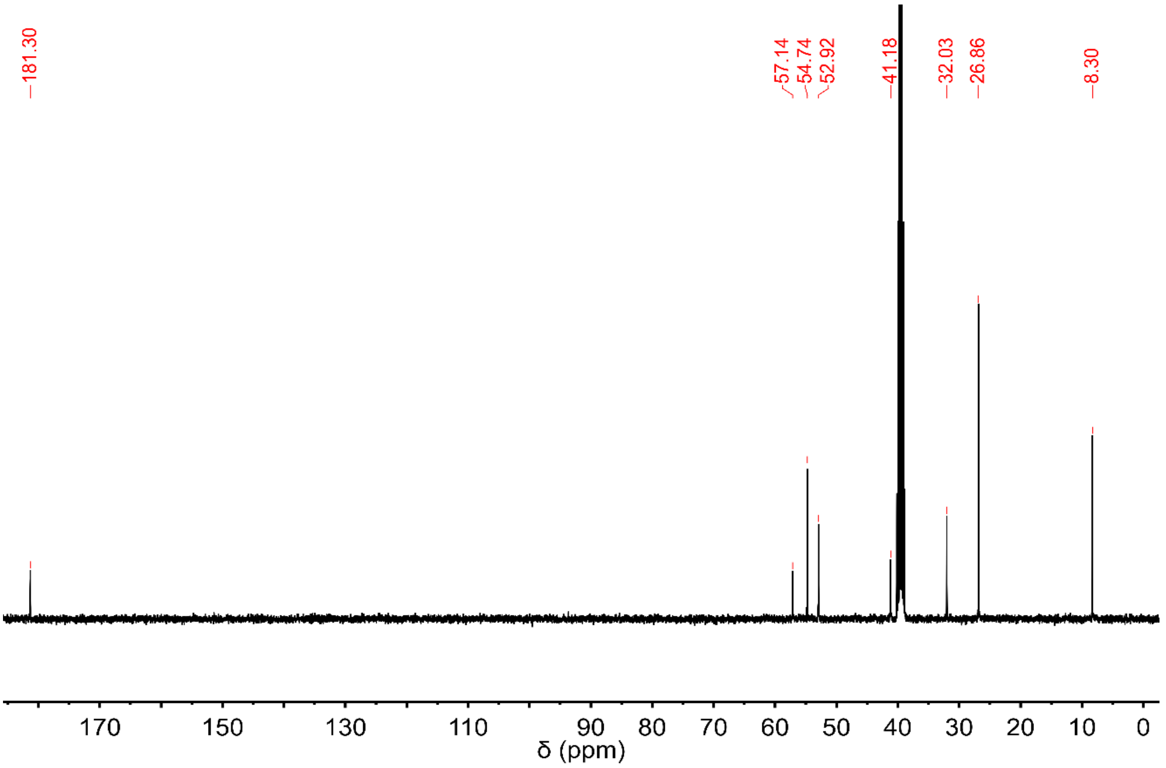

Figure 33. ^{13}C NMR (101 MHz) spectrum 1,1'-(((2-aminoethyl)azanediyl)bis(ethane-2,1-diyl))bis(3-(*tert*-pentyl)thiourea) in $(CD_3)_2SO$ at 298 K.

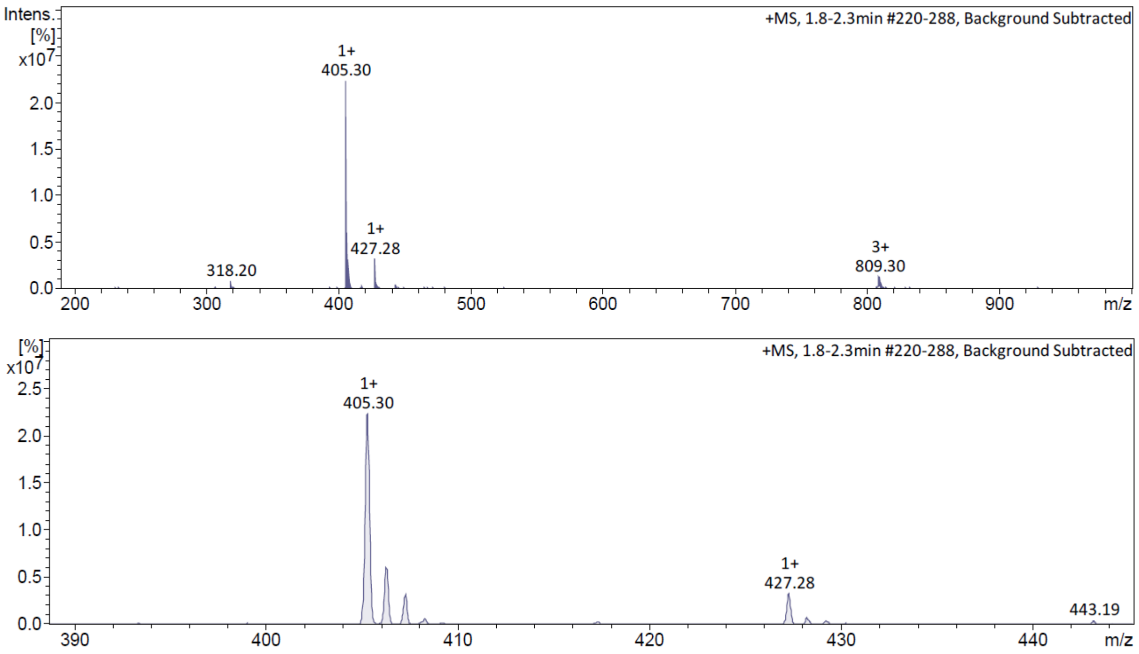

Figure 34. LR-MS (ESI$^+$) of 1,1'-(((2-aminoethyl)azanediyl)bis(ethane-2,1-diyl))bis(3-(*tert*-pentyl)thiourea).

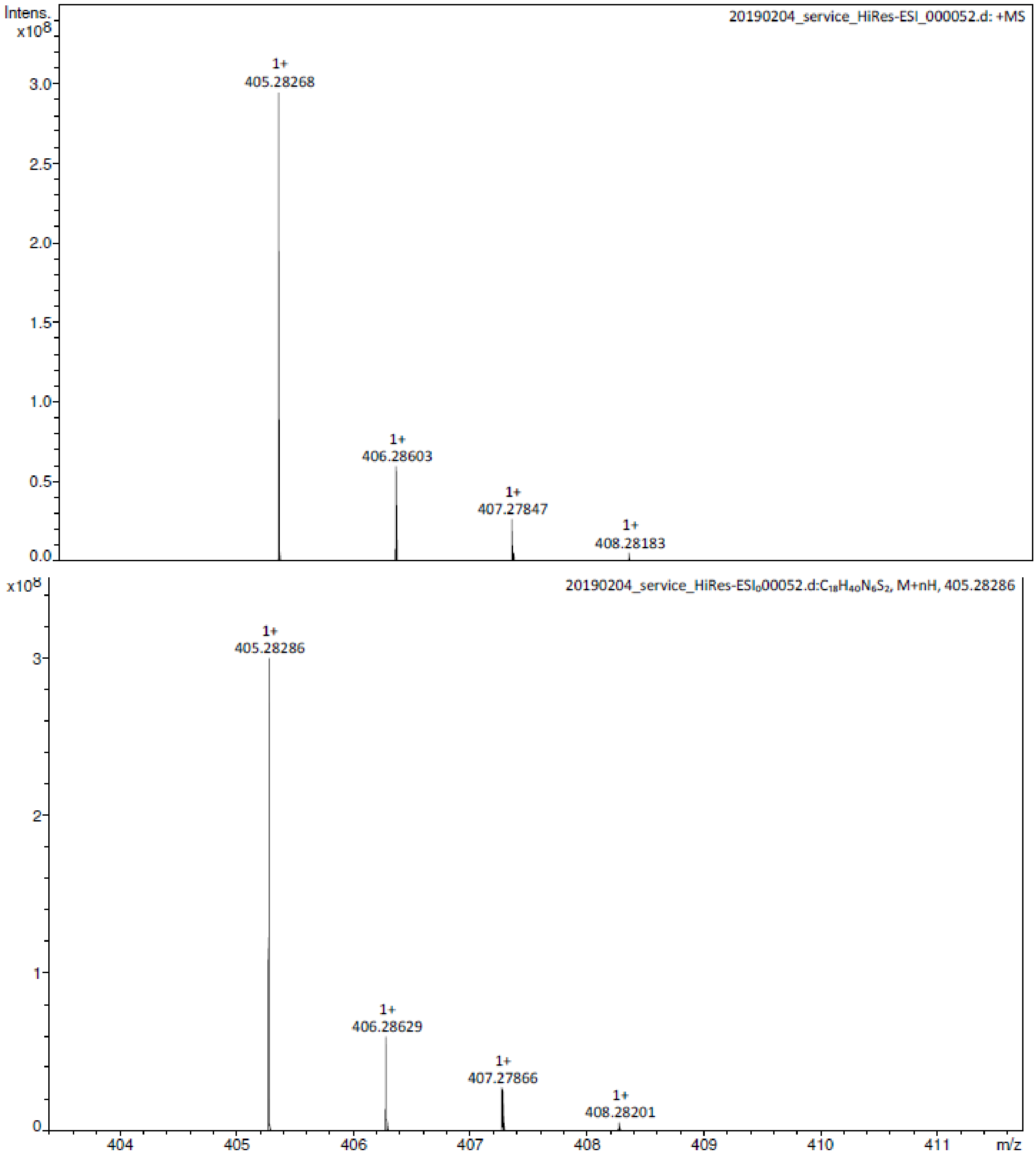

Figure 35. HR-MS (ESI$^+$) of 1,1'-(((2-aminoethyl)azanediyl)bis(ethane-2,1-diyl))bis(3-(*tert*-pentyl)thiourea).

1-(2-(Bis(2-(3-(*tert*-pentyl)thioureido)ethyl)amino)ethyl)-3-pentylthiourea

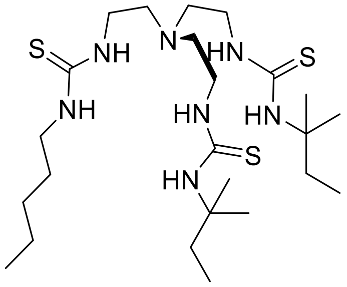

1,1'-(((2-aminoethyl)azanediyl)bis(ethane-2,1-diyl))bis(3-(*tert*-pentyl)thiourea) (100 mg, 0.25 mmol) was dissolved in DCM. 1-Pentylisothiocyanate (0.04 mL, 0.29 mmol) was added dropwise and the mixture was stirred at room temperature for 2 hr. After 2 hr solvent was removed and the crude product was purified by column chromatography (silica, DCM → 3% MeOH) to give a white crystalline solid (0.109 g, 83 %).

^{1}H NMR (400 MHz, DMSO-d_6) δ ppm 7.46 (br s, 1 H) 7.18 (br s, 1 H) 7.09 (t, *J*=4.7 Hz, 2 H) 6.95 (s, 2 H) 3.45-3.44 (m, 6 H) 3.31 (br s, 2 H) 2.63-2.58 (m, 6 H), 1.85 (q, *J*=7.3 Hz, 4 H) 1.49-1.41 (m, 4 H) 1.33 (s, 12 H) 1.32-1.24 (m, 4 H) 0.86 (t, *J*=6.9 Hz, 3 H) 0.76 (t, *J*=7.4 Hz, 6 H); **^{13}C NMR** (101 MHz, DMSO-d_6) δ ppm 181.30, 54.92, 54.78, 52.62, 52.53, 41.12, 32.05, 28.64, 28.46, 26.85, 21.92, 13.86, 8.31

LR-MS (ESI$^+$) 534.37 $[M + H]^+$, 556.35 $[M + Na]^+$; **HR-MS** (ESI$^+$) calcd for $C_{24}H_{51}N_7S_3$ $[M+H]^+$: 534.34408, found *m/z* 534.34373

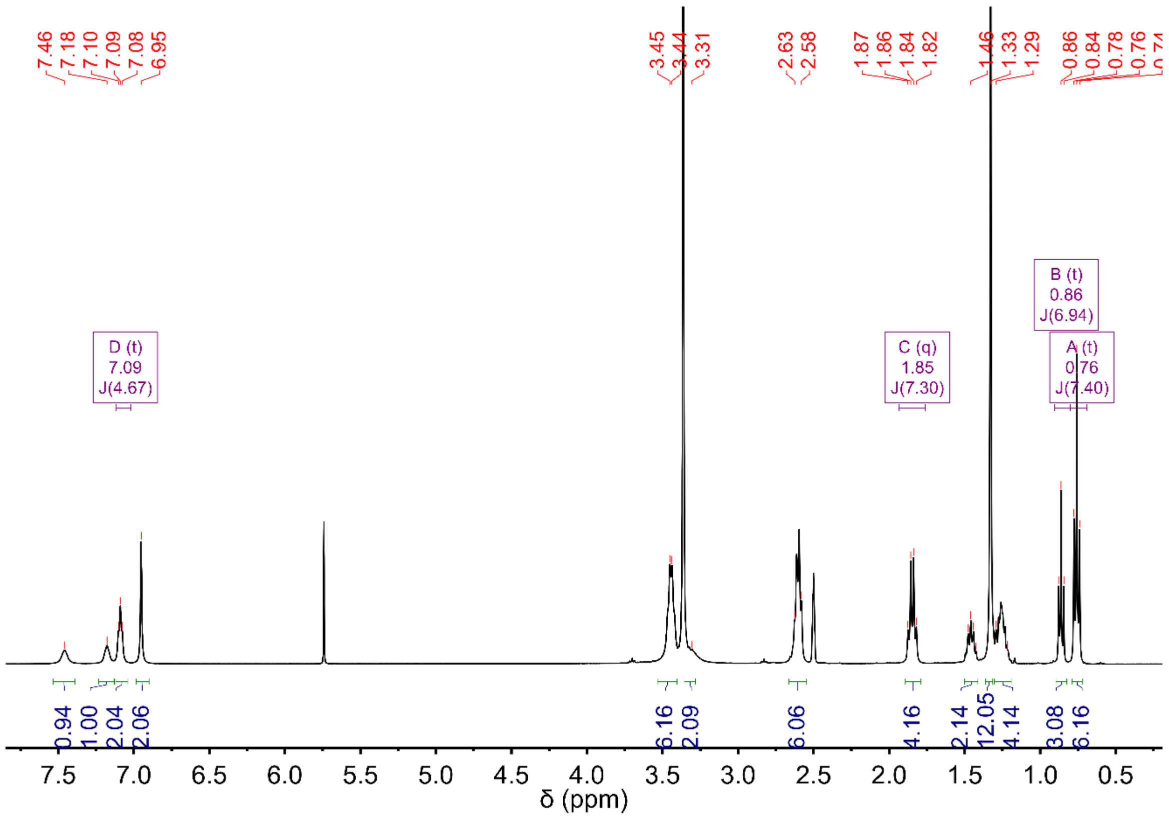

Figure 36. ^{1}H NMR (400 MHz) spectrum of 1-(2-(bis(2-(3-(*tert*-pentyl)thioureido)ethyl)amino)ethyl)-3-pentylthiourea in $(CD_3)_2SO$ at 298 K.

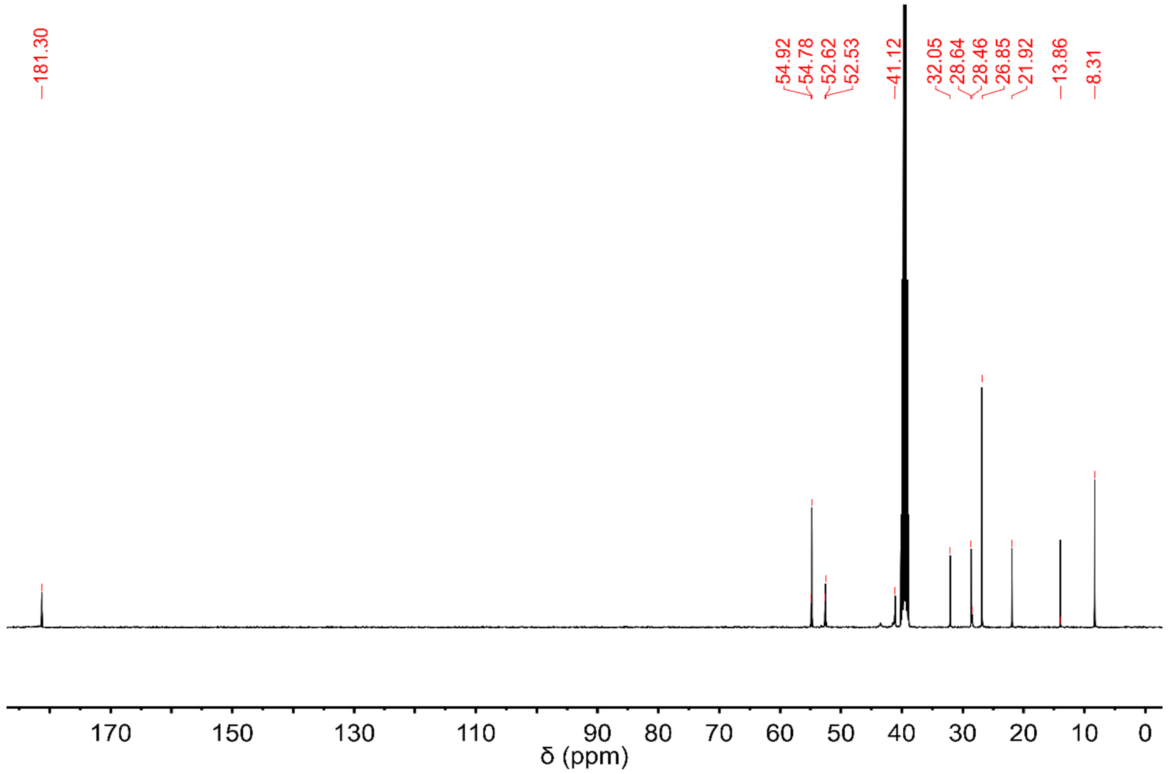

Figure 37. ^{13}C NMR (101 MHz) spectrum of 1-(2-(bis(2-(3-(*tert*-pentyl)thioureido)ethyl)amino)ethyl)-3-pentylthiourea in $(CD_3)_2SO$ at 298 K.

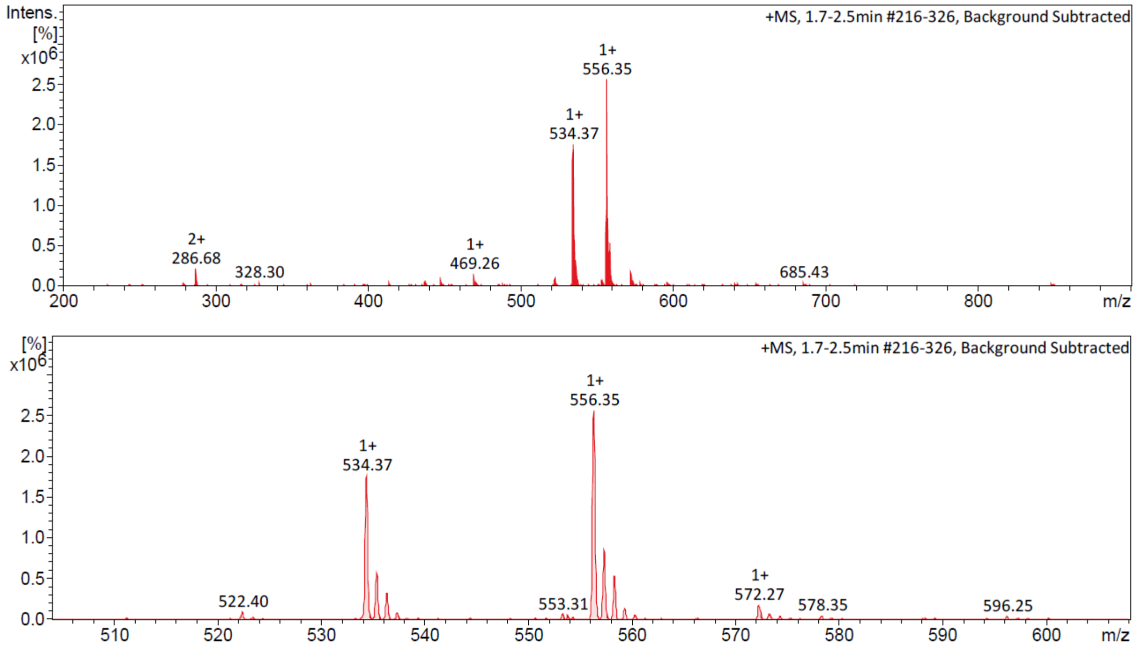

Figure 38. LR-MS (ESI$^+$) of 1-(2-(bis(2-(3-(*tert*-pentyl)thioureido)ethyl)amino)ethyl)-3-pentylthiourea.

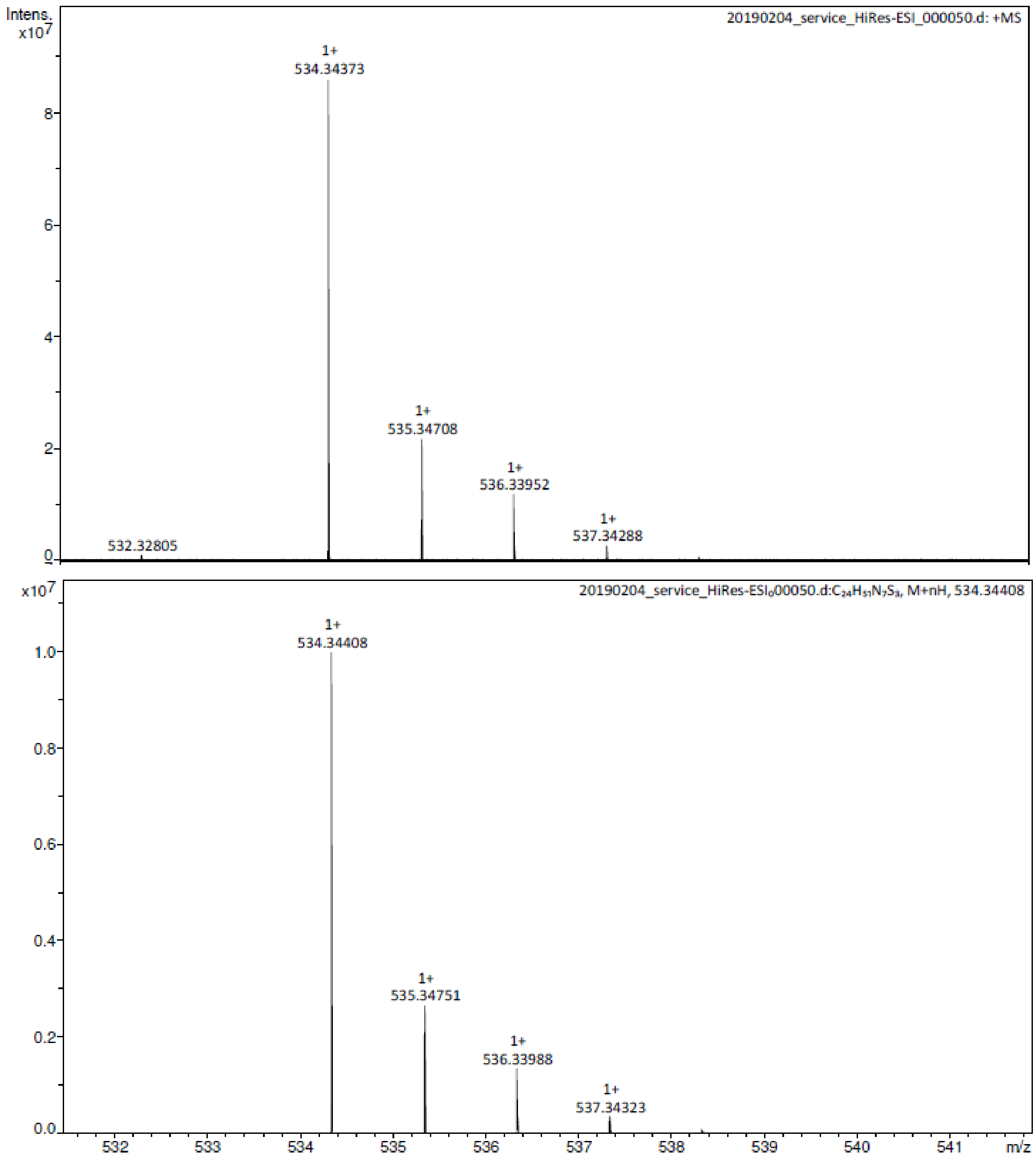

Figure 39. HR-MS (ESI$^+$) of 1-(2-(bis(2-(3-(*tert*-pentyl)thioureido)ethyl)amino)ethyl)-3-pentylthiourea.

tert-Butyl (2-(2,10-dithioxo-1,3,6,9,11-pentaazacyclooctadecan-6-yl)ethyl)carbamate

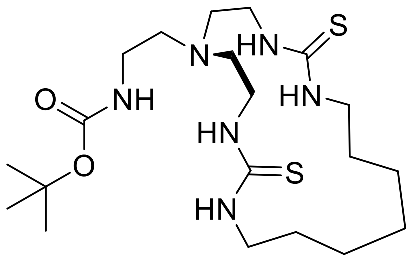

tert-Butyl (2-(bis(2-isothiocyanatoethyl)amino)ethyl)carbamate (250 mg, 0.756 mmol) was dissolved in anhydrous DCM (10 mL). 1,7-diaminoheptane (0.11 mL, 0.756 mmol) was dissolved in anhydrous DCM (10 mL). The two solutions were added dropwise simultaneously to anhydrous DCM (120 mL) at room temperature over 1 hr. The mixture was stirred for 14 days at room temperature. After 14 days solvent was removed, and the crude product was purified by column chromatography (silica, DCM → 3% MeOH) to give a white crystalline solid (0.106 g, 30 %).

^{1}H NMR (400 MHz, DMSO-d_6) δ ppm 7.42 (br s, 2 H) 7.15 (br s, 2 H) 6.69 (br s, 1 H) 3.42 (br s, 6 H) 3.21 (br s, 2 H) 2.99-2.96 (m, 2 H) 2.63 (s, 4 H) 2.55-2.52 (m, 2 H) 1.46 (br s, 4 H) 1.37 (s, 9 H) 1.27 (s, 6 H); **^{13}C NMR** (101 MHz, DMSO-d_6) δ ppm 181.51, 155.60, 79.18, 77.60, 52.91, 52.08, 42.66, 41.85, 37.99, 33.36, 28.26, 27.51, 26.80, 25.20, 24.48

LR-MS (ESI$^+$) *m/z* 461.28 [M + H]$^+$, 483.25 [M + Na]$^+$; **HR-MS** (ESI$^+$) calcd for $C_{20}H_{40}N_6O_2S_2$ [M+H]$^+$: 461.27269, found *m/z* 461.27242

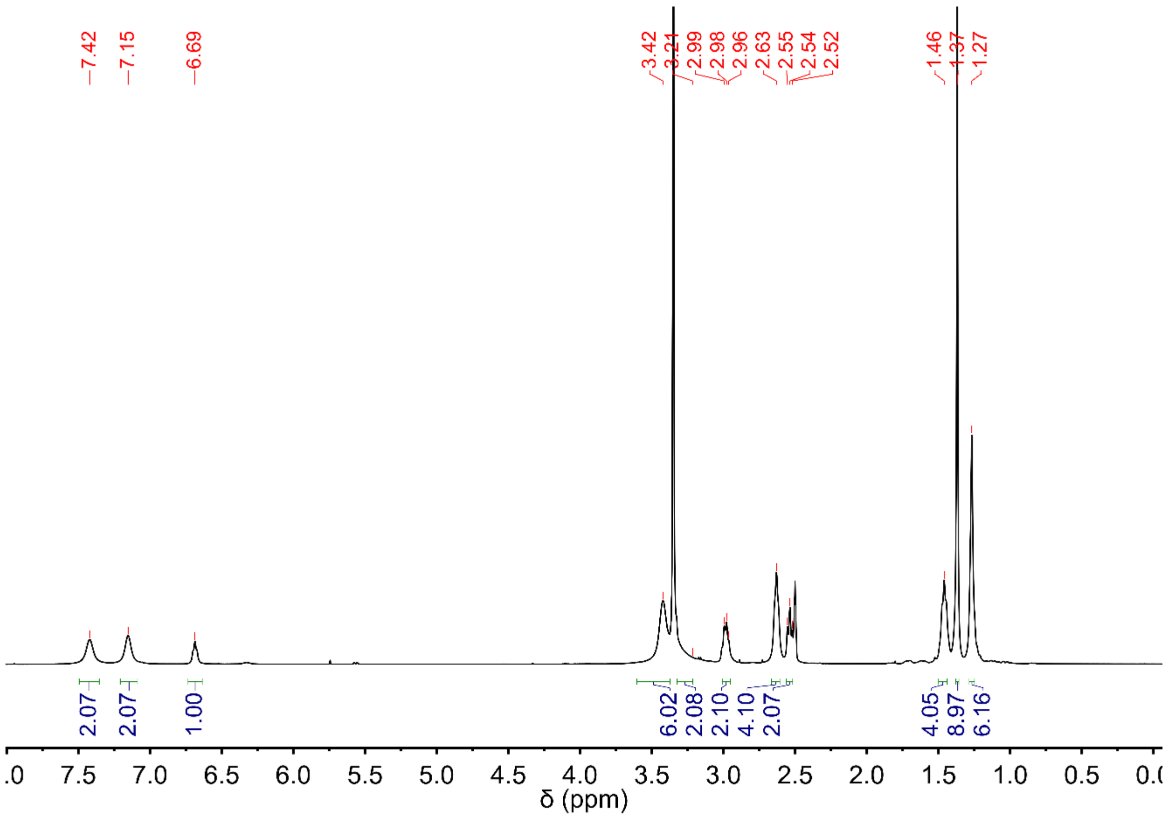

Figure 40. ^1H NMR (400 MHz) spectrum of *tert*-butyl (2-(2,10-dithioxo-1,3,6,9,11-pentaazacyclooctadecan-6-yl)ethyl)carbamate in $(CD_3)_2SO$ at 298 K.

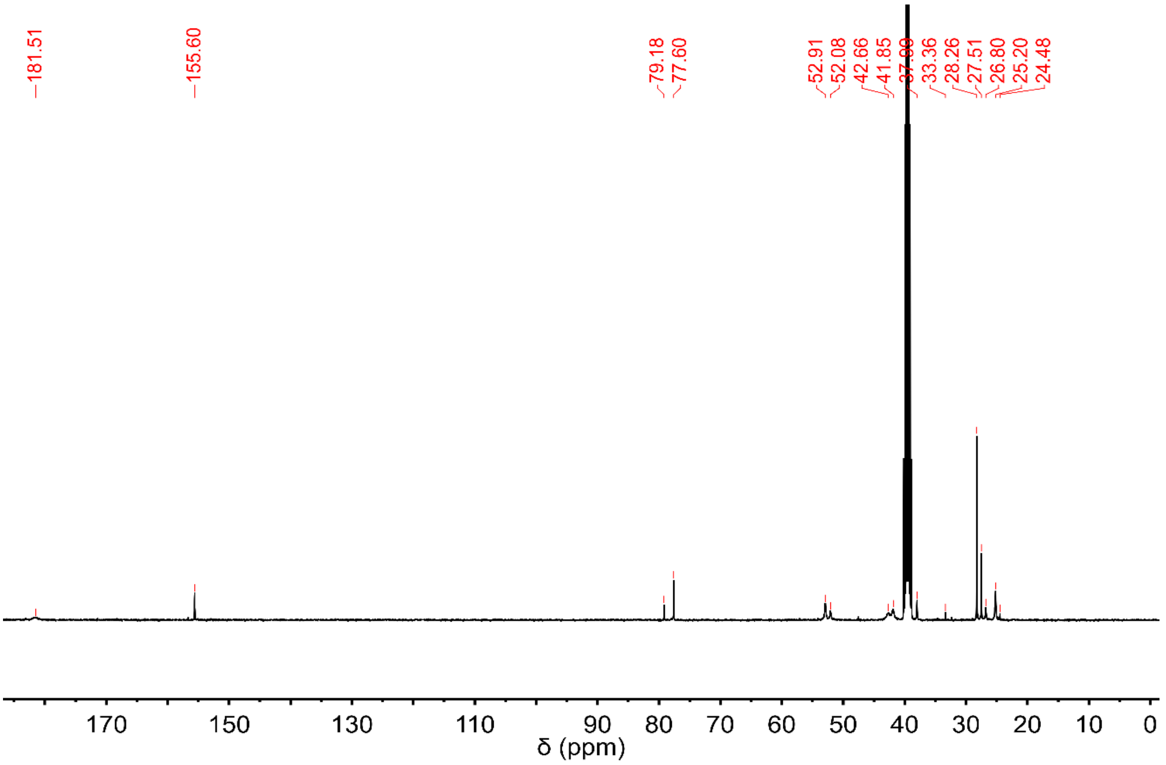

Figure 41. ^{13}C NMR (101 MHz) spectrum of *tert*-butyl (2-(2,10-dithioxo-1,3,6,9,11-pentaazacyclooctadecan-6-yl)ethyl)carbamate in $(CD_3)_2SO$ at 298 K.

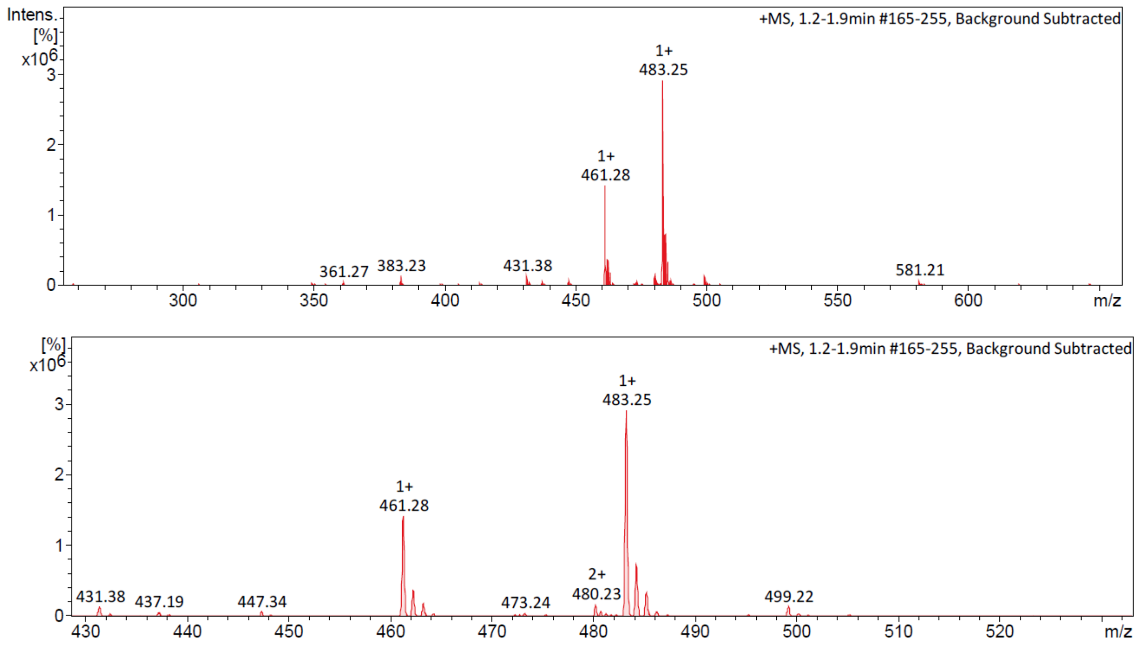

Figure 42. LR-MS (ESI$^+$) of *tert*-butyl (2-(2,10-dithioxo-1,3,6,9,11-pentaazacyclooctadecan-6-yl)ethyl)carbamate.

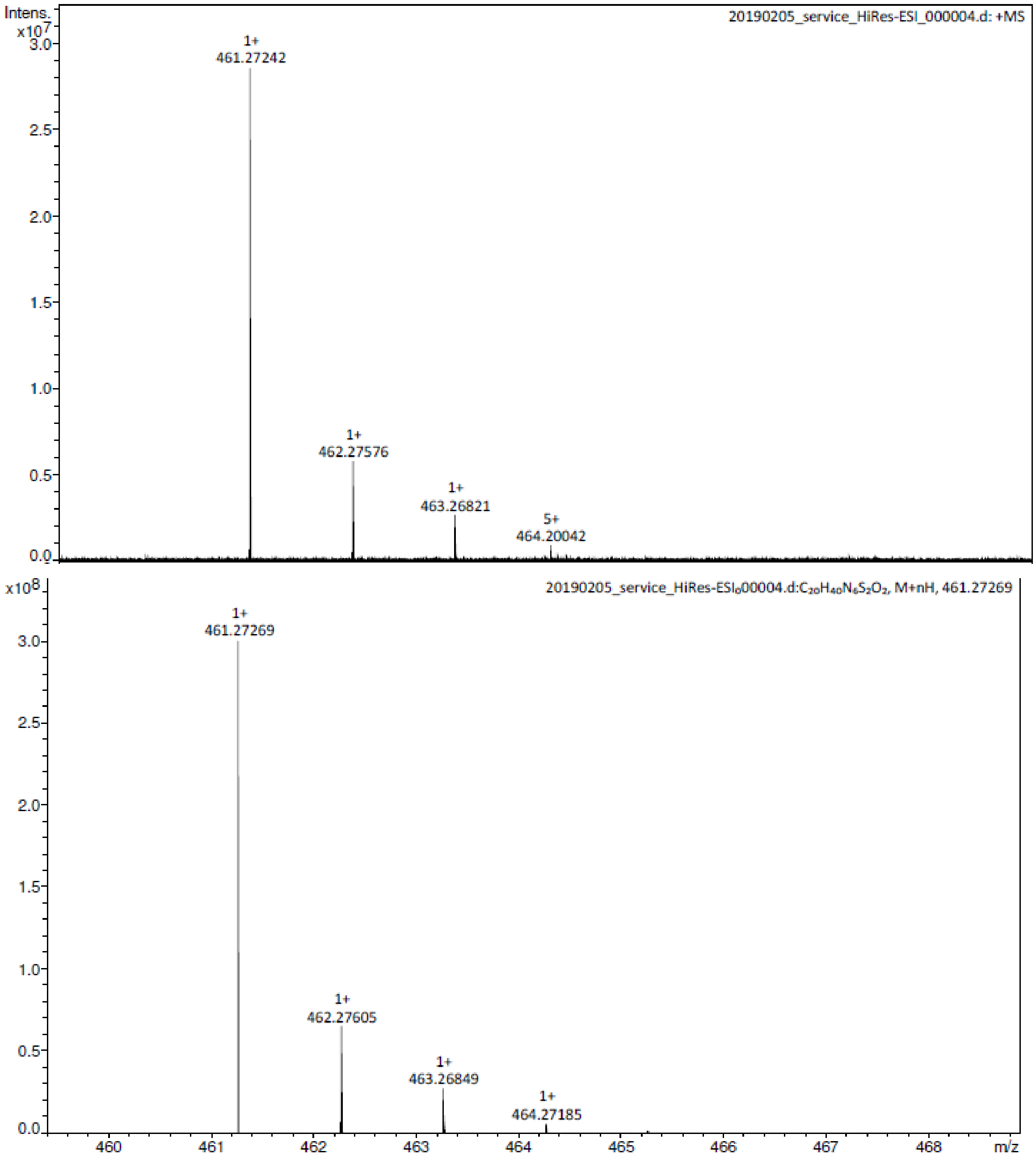

Figure 43. HR-MS (ESI$^+$) of *tert*-butyl (2-(2,10-dithioxo-1,3,6,9,11-pentaazacyclooctadecan-6-yl)ethyl)carbamate.

1-(2-(2,10-Dithioxo-1,3,6,9,11-pentaazacyclooctadecan-6-yl)ethyl)-3-(*tert*-pentyl)thiourea

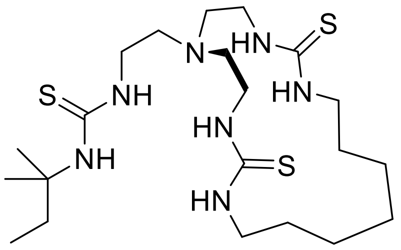

tert-Butyl (2-(2,10-dithioxo-1,3,6,9,11-pentaazacyclooctadecan-6-yl)ethyl)carbamate (100 mg, 0.217 mmol) was dissolved in $CHCl_3$. TFA (1 mL) was added dropwise and the mixture was stirred at room temperature for 3 hr. Solvent was removed and the residue was washed with toluene (3 x 5 mL) The glass-like solid was immediately dissolved in anhydrous DMF (4 mL) and TEA (0.8 mL, 5.74 mmol) was added dropwise. 2-Isothiocyanato-2-methylbutane (0.070 g, 0.545 mmol) was added to the reaction mixture dropwise and the solution was stirred at room temperature for 18 hr. After 18 hr solvent was removed and the crude product was purified by column chromatography (silica, DCM → 5% MeOH) to give a white crystalline solid (0.020 g, 7 %).

^{1}H NMR (400 MHz, DMSO-d_6) δ ppm 7.46 (br s, 2 H) 7.22 (br s, 3 H) 7.05 (br s, 1 H) 3.44 (br s, 6 H) 3.31 (br s, 4 H) 2.67 (br s, 6 H), 1.85 (q, *J*=7.3 Hz, 2H) 1.45 (br s, 4 H) 1.33 (s, 6 H) 1.26 (br s, 8 H) 0.76 (t, *J*=7.4 Hz, 3 H); **^{13}C NMR** (101 MHz, DMSO-d_6) δ ppm 181.34, 79.19, 54.79, 52.92, 51.10, 41.80, 40.95, 32.03, 27.69, 27.03, 26.85, 25.31, 22.12, 13.94, 8.32

LR-MS (ESI$^+$) *m/z* 490.29 $[M + H]^+$, 512.27 $[M + Na]^+$; **HR-MS** (ESI$^+$) calcd for $C_{21}H_{43}N_7S_3$ $[M+H]^+$:490.28148, found *m/z* 490.28122

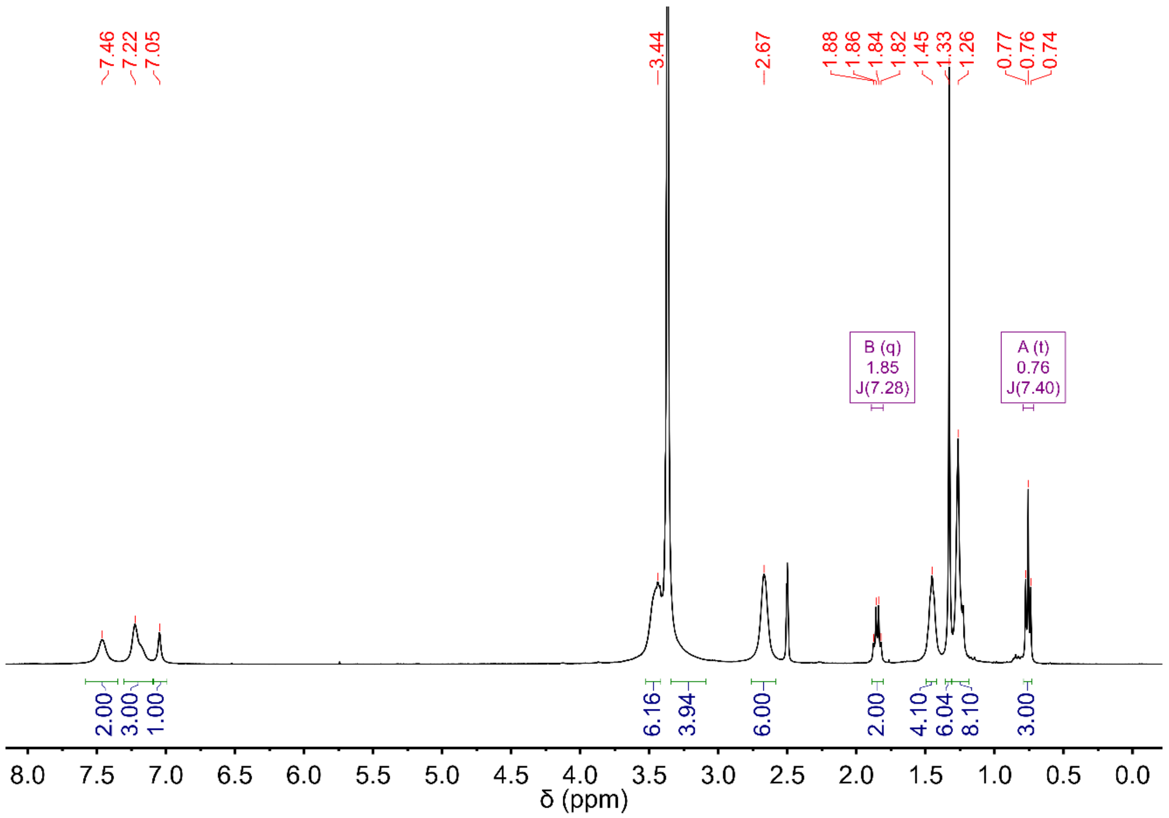

Figure 44. ^{1}H NMR (400 MHz) spectrum of 1-(2-(2,10-dithioxo-1,3,6,9,11-pentaazacyclooctadecan-6-yl)ethyl)-3-(*tert*-pentyl)thiourea in $(CD_3)_2SO$ at 298 K.

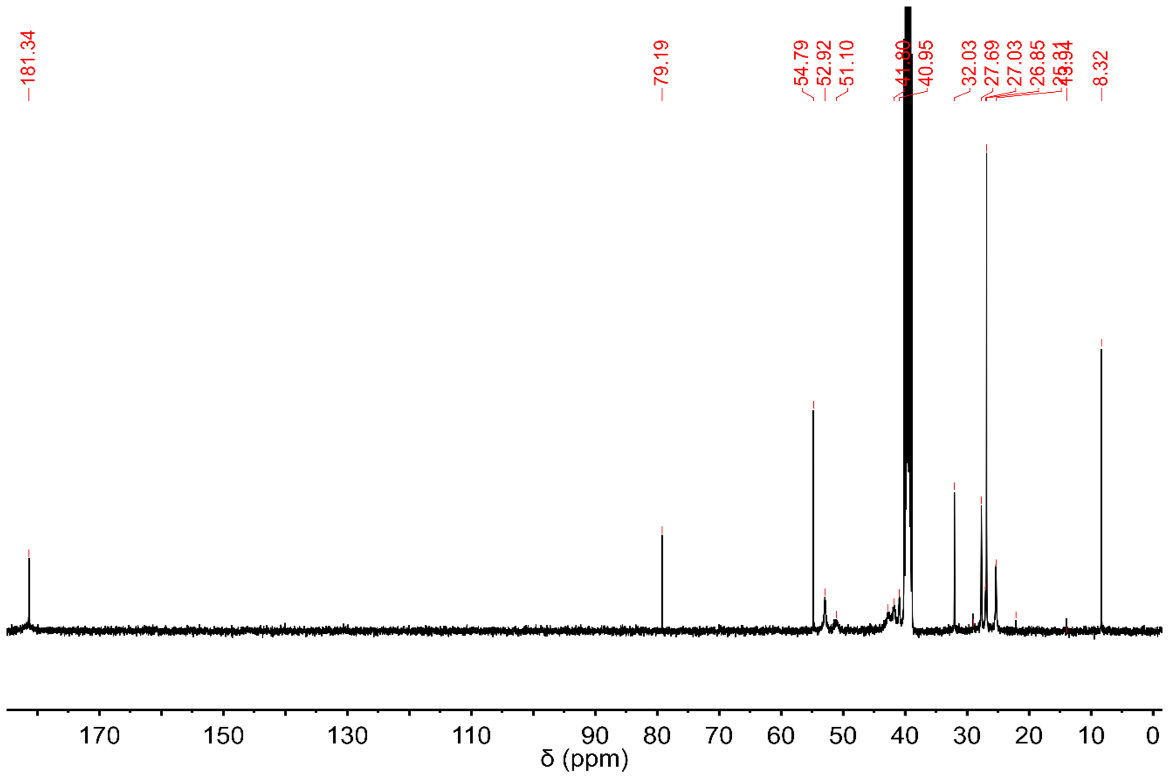

Figure 45. ^{13}C NMR (101 MHz) spectrum of 1-(2-(2,10-dithioxo-1,3,6,9,11-pentaazacyclooctadecan-6-yl)ethyl)-3-(*tert*-pentyl)thiourea in $(CD_3)_2SO$ at 298 K.

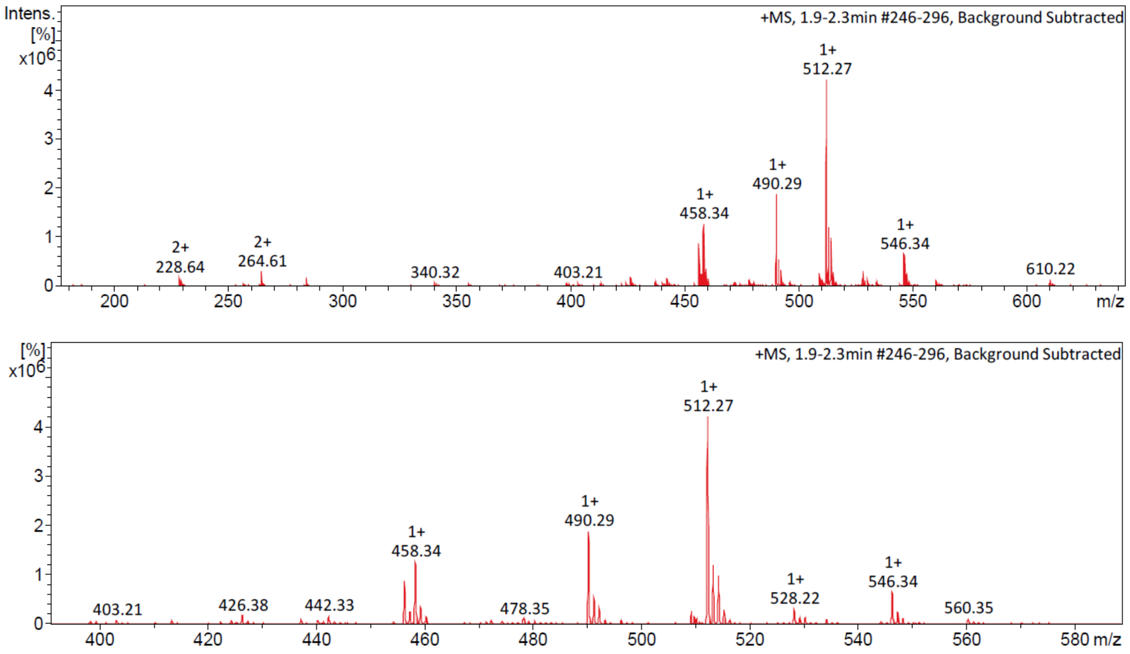

Figure 46. LR-MS (ESI$^+$) of 1-(2-(2,10-dithioxo-1,3,6,9,11-pentaazacyclooctadecan-6-yl)ethyl)-3-(*tert*-pentyl)thiourea.

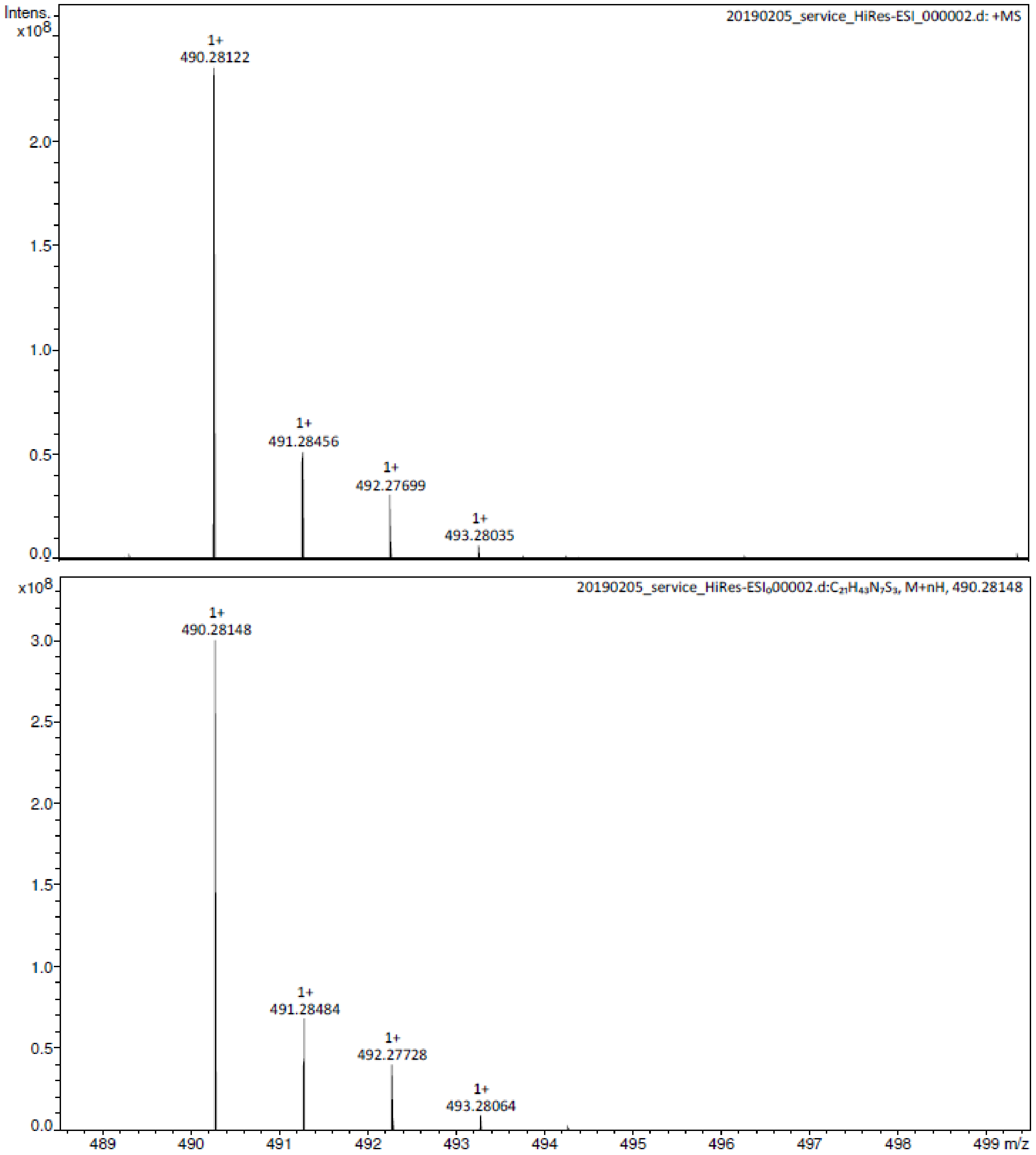

Figure 47. HR-MS (ESI$^+$) of 1-(2-(2,10-dithioxo-1,3,6,9,11-pentaazacyclooctadecan-6-yl)ethyl)-3-(*tert*-pentyl)thiourea.

1.1.2 ^{1}H NMR Titration Anion Binding Studies

Equipment and Sample Preparation

^{1}H NMR titrations were performed on a Bruker Avance DPX 400 spectrometer. For NMR titrations with chloride, a constant host concentration was maintained (~ 5.0 mM) by using the host solution to dissolve the guest to make the guest stock solution. For titrations performed with bicarbonate and phosphate, a host concentration of ~ 1 mM was maintained. Over the course of the titration Hamiltonian Microlitre syringes were used to add aliquots of the guest stock solution to the NMR sample of the host solution.

The anions were added as the tetrabutylammonium (TBA) salts after being dried under high vacuum (< 1.0 mmHg) for 24 h. Stock solutions of the host were prepared in a DMSO-d_6/0.5%H_2O mixture. The host stock solutions (500 µL) were transferred to an air-tight screw-cap NMR sample tube (5 mm ID) and the same host stock solution was used to prepare the standard guest titrant solution containing 20-100 mM of the TBA-anion salts. This ensured a constant concentration of the host for the duration of the titration experiment.

Titration Procedure

Over the course of the titration small aliquots (2-100 µL) of the standard guest solution were added to the host solution (~ 500 µL) in the NMR tube. For each titration 15-20 data points were collected and at the end of the titration approximately 10 equivalents of the guest anion salt were present.

Upon each addition of the standard guest solution the samples were thoroughly shaken in the NMR tube and then allowed to equilibrate for up to 2 minutes inside the NMR probe before the spectra were taken. Throughout each titration experiment all parameters of the NMR spectrometer remained constant.

Titration Data Fitting

In all cases the proton resonances were monitored for changes in chemical shift. Where possible two or more resonances were followed, allowing several data sets to use in determination of the association constant (K_a). Global fitting takes into account all data sets at the same time and improves the quality of the nonlinear curve fitting. The supramolecular.org web applet was used to fit the titration data for all alkyl tren compounds **1**-**7** with chloride and bicarbonate to a 1:1 model. Where possible titrations with di-hydrogen phosphate were fitted to the 1:1 model, but in the cases where the peaks broadened into the base line the data could not be fitted.

Titration Spectra

^{1}H NMR titration spectra and fitted binding isotherms shown for each receptor. The host concentration is included in the caption for each titration.

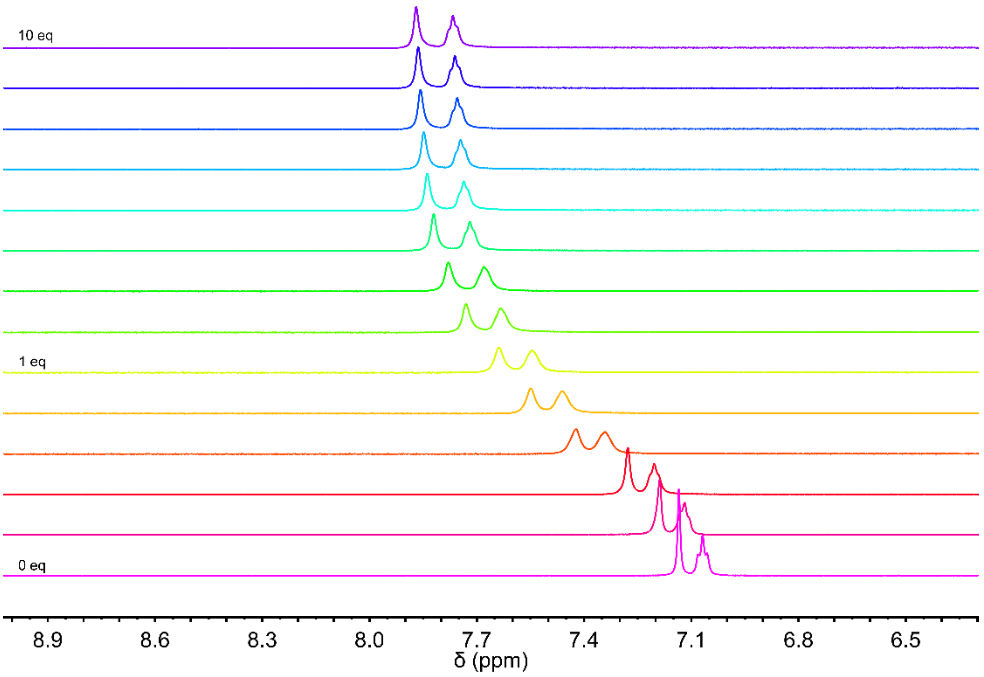

Figure 48. ^{1}H NMR titration spectra as a stack plot for t-butyl tren (5 mM) + TBACl in DMSO-d_6/0.5% H_2O at 298 K.

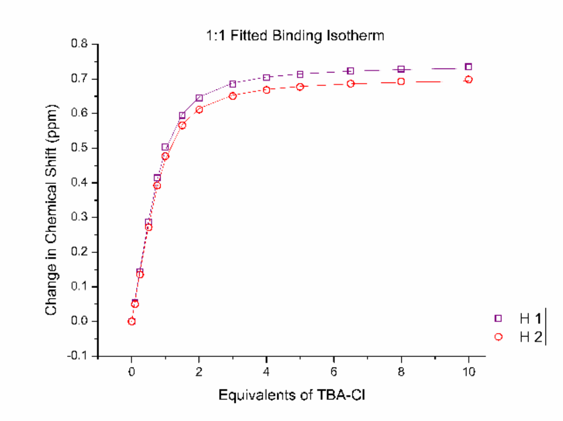

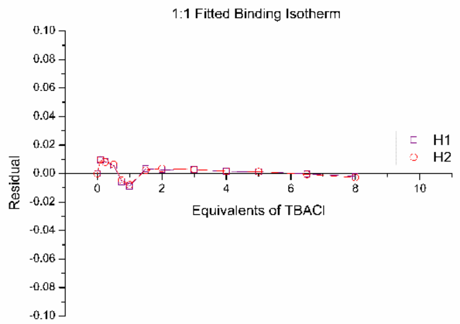

Figure 49. Fitted binding isotherm of t-butyl tren + TBACl showing the change in chemical shift of the NH protons fitted to the 1:1 binding model (left). K_a = 1110 M^{-1} Residual plot showing the random error obtained from the binding isotherm fitting (right).

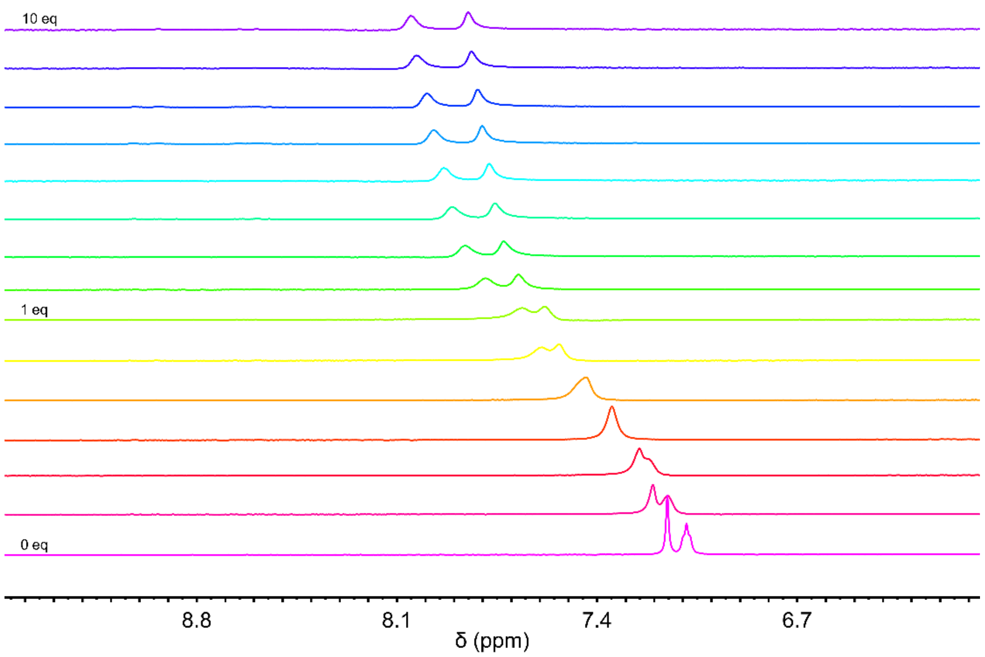

Figure 50. ^{1}H NMR titration spectra as a stack plot for t-butyl tren (1 mM) + $TEAHCO_3$ in DMSO-d_6/o.5% H_2O at 298 K.

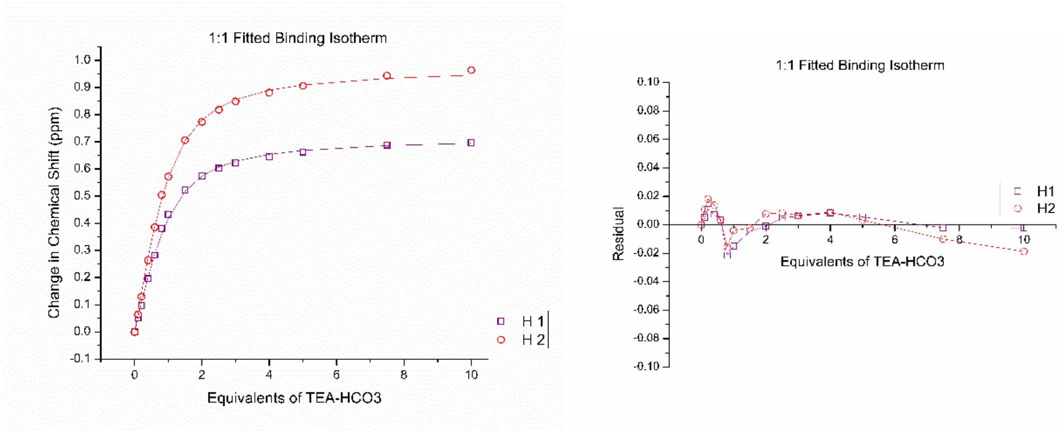

Figure 51. Fitted binding isotherm of t-butyl tren + $TEAHCO_3$ showing the change in chemical shift of the NH protons fitted to the 1:1 binding model (left). K_a = 2670 M^{-1} Residual plot showing the random error obtained from the binding isotherm fitting (right).

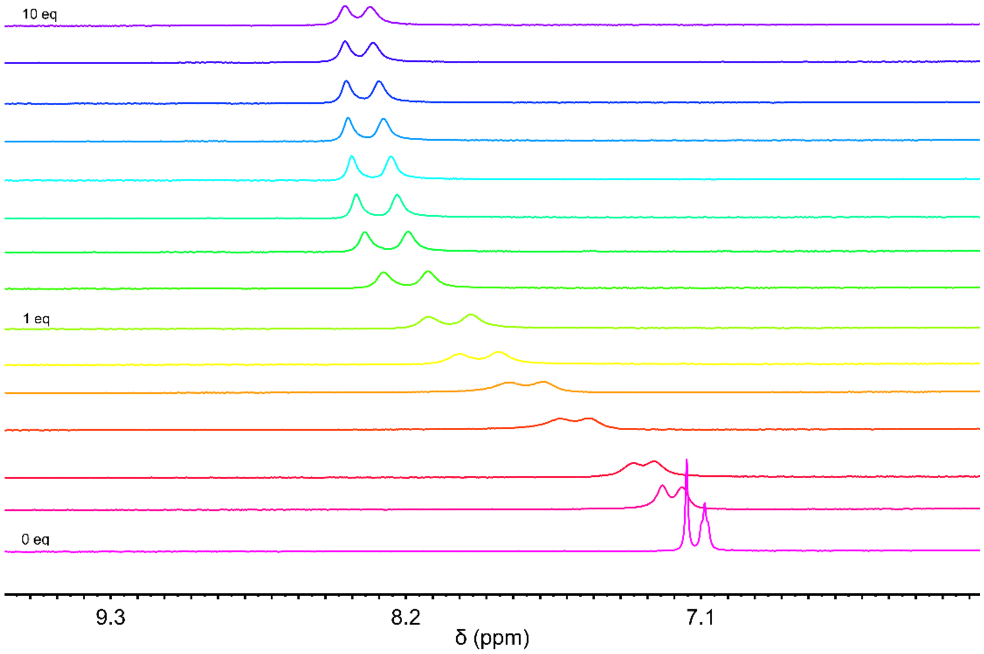

Figure 52. ^{1}H NMR titration spectra as a stack plot for t-butyl tren (1 mM) + $TBAH_2PO_4$ in DMSO-d_6/o.5% H_2O at 298 K.

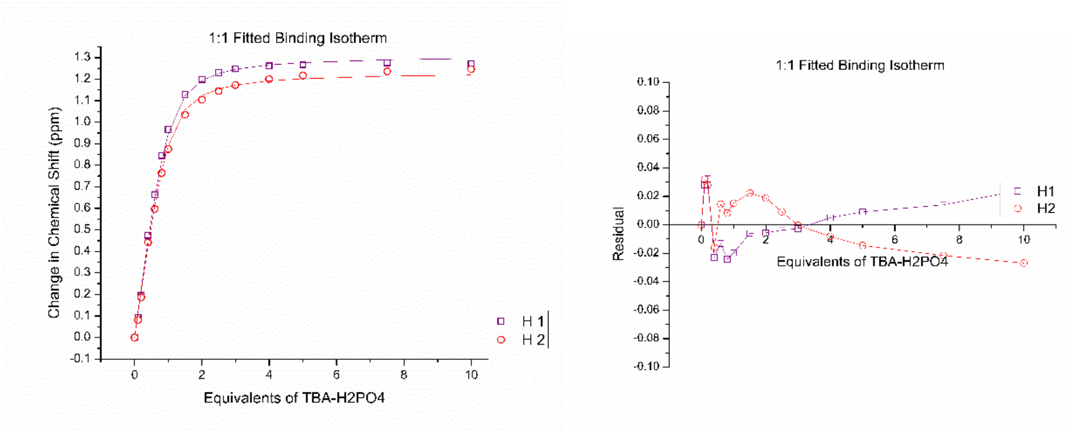

Figure 53. Fitted binding isotherm of t-butyl tren + $TBAH_2PO_4$ showing the change in chemical shift of the NH protons fitted to the 1:1 binding model (left). K_a = 7460 M^{-1} Residual plot showing the random error obtained from the binding isotherm fitting (right).

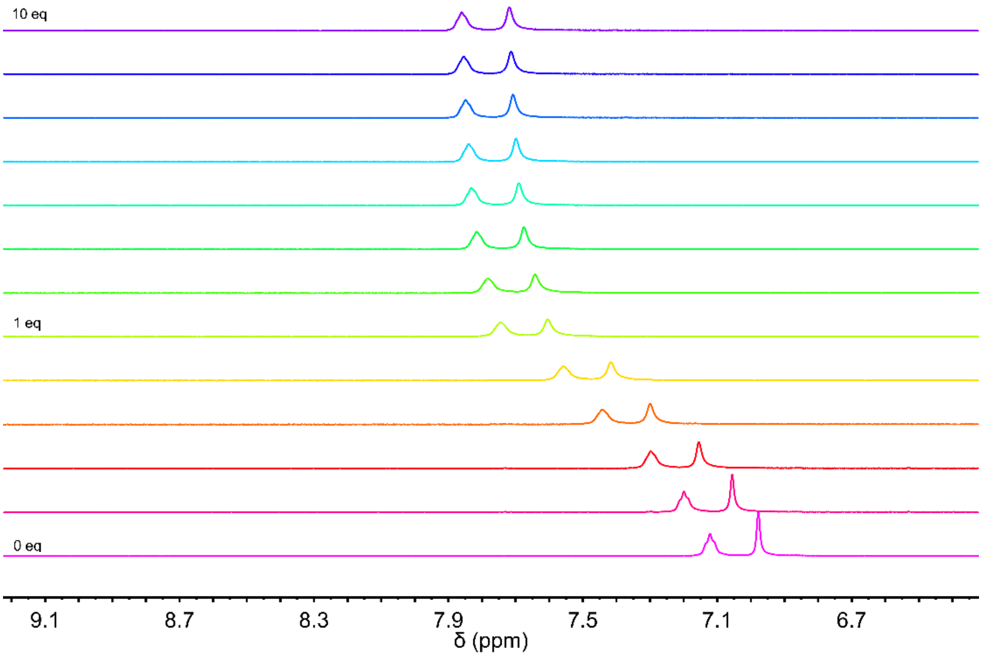

Figure 54. ^{1}H NMR titration spectra as a stack plot for t-pentyl tren (5 mM) + TBACl in DMSO-d_6/o.5% H_2O at 298 K.

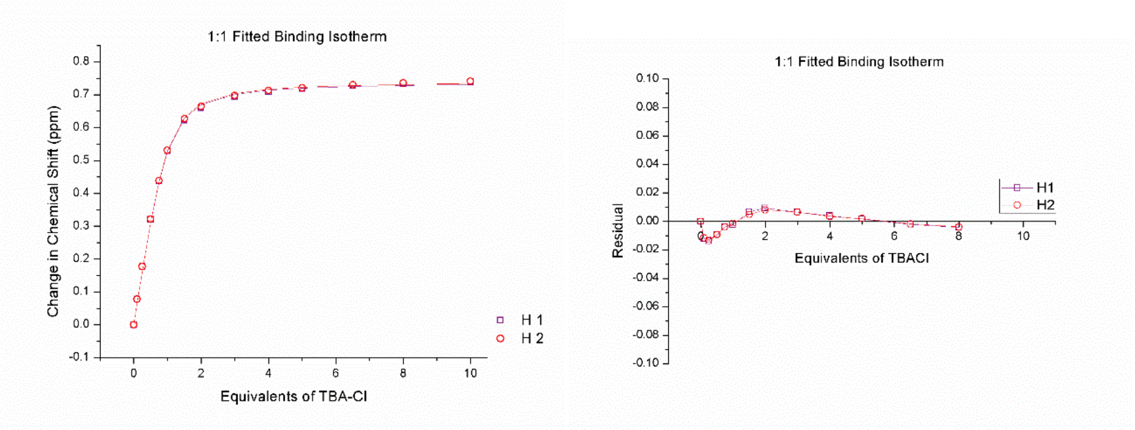

Figure 55. Fitted binding isotherm of t-pentyl tren + TBACl showing the change in chemical shift of the NH protons fitted to the 1:1 binding model (left). K_a = 1530 M^{-1} Residual plot showing the random error obtained from the binding isotherm fitting (right).

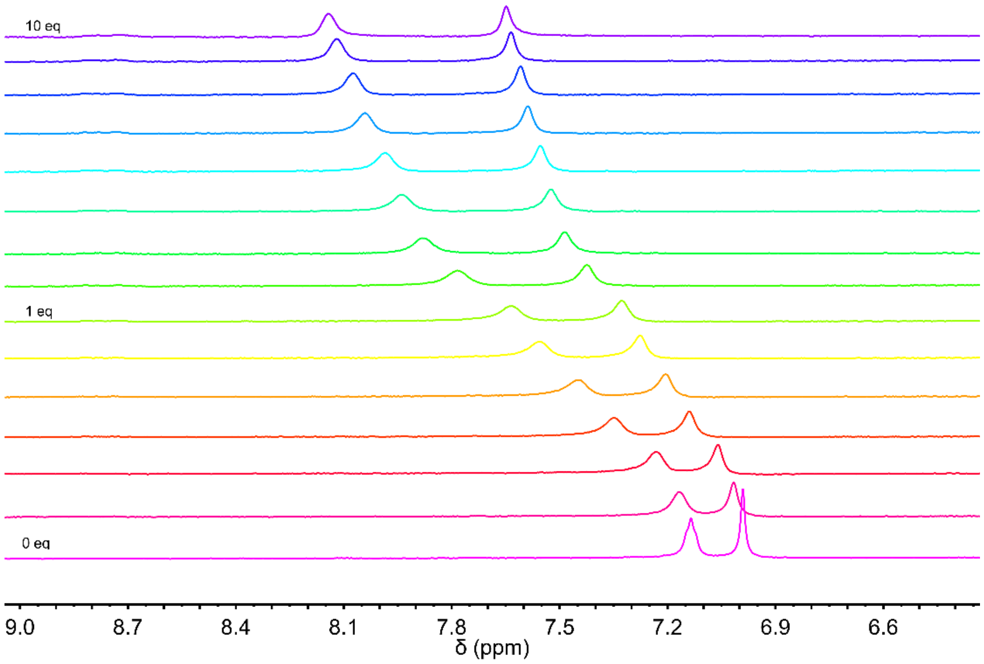

Figure 56. ^{1}H NMR titration spectra as a stack plot for t-pentyl tren (1 mM) + $TEAHCO_3$ in DMSO-d_6/0.5% H_2O at 298 K.

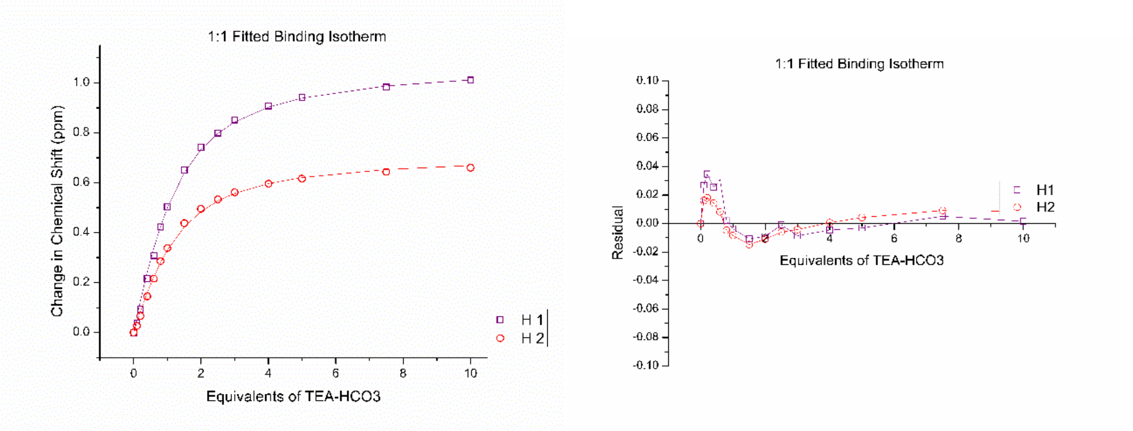

Figure 57. Fitted binding isotherm of t-pentyl tren + $TEAHCO_3$ showing the change in chemical shift of the NH protons fitted to the 1:1 binding model (left). K_a = 1500 M^{-1} Residual plot showing the random error obtained from the binding isotherm fitting (right).

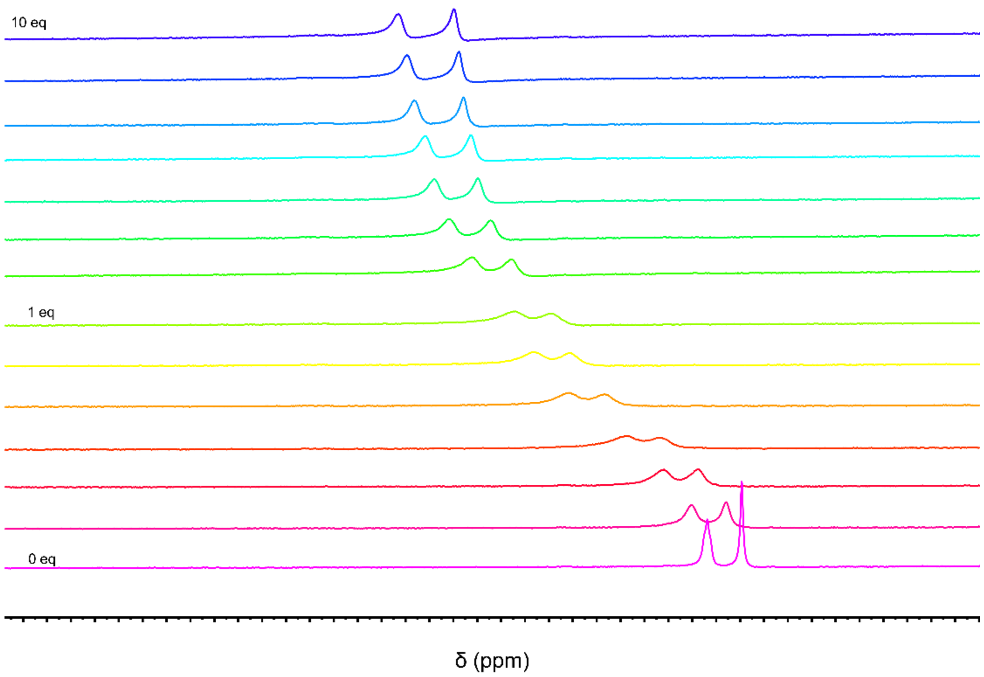

Figure 58. ^{1}H NMR titration spectra as a stack plot for t-pentyl tren (1 mM) + $TBAH_2PO_4$ in DMSO-d_6/o.5% H_2O at 298 K.

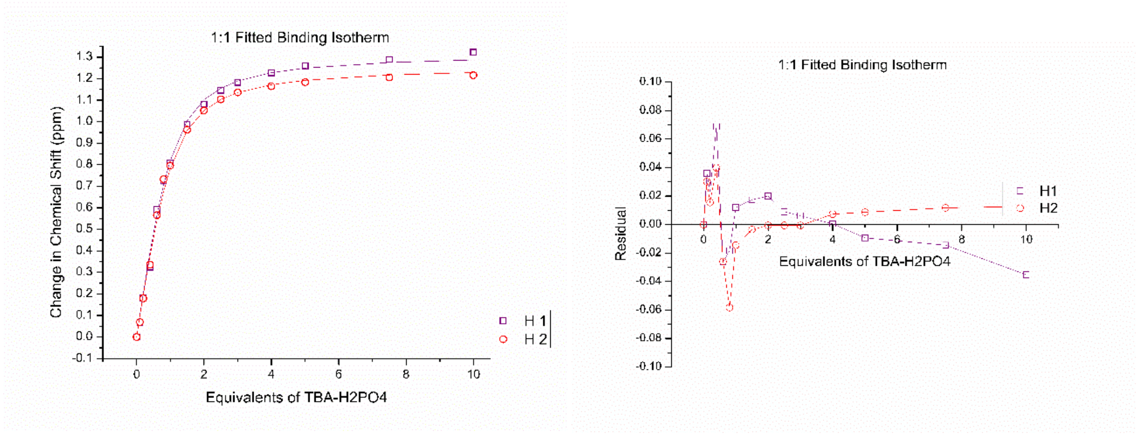

Figure 59. Fitted binding isotherm of t-pentyl tren + $TBAH_2PO_4$ showing the change in chemical shift of the NH protons fitted to the 1:1 binding model (left). K_a = 4080 M^{-1} Residual plot showing the random error obtained from the binding isotherm fitting (right).

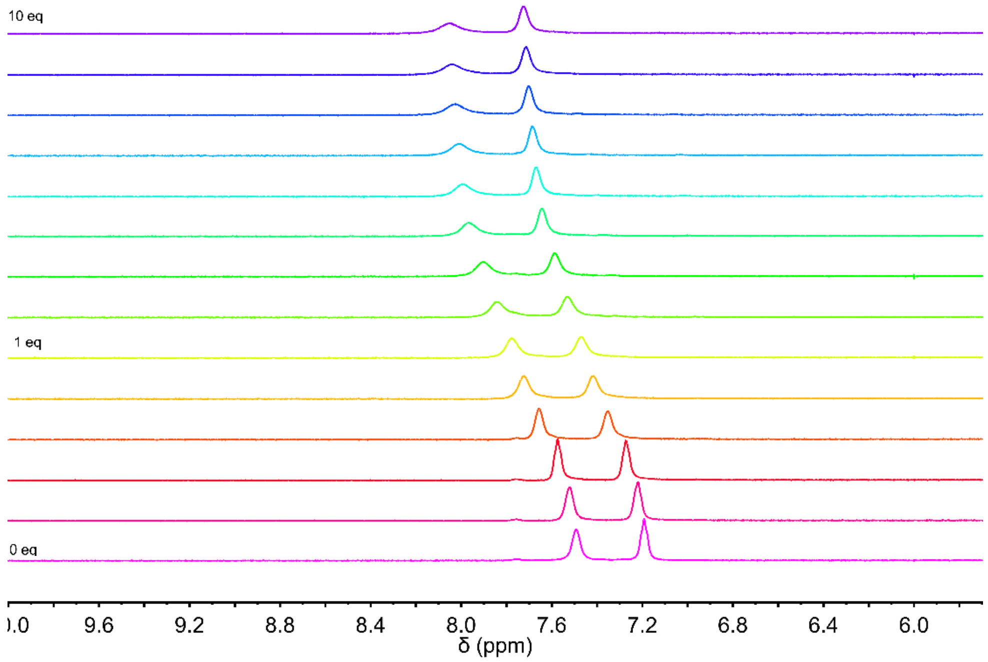

Figure 60. ^{1}H NMR titration spectra as a stack plot for n-pentyl tren (5 mM) + TBACl in DMSO-d_6/o.5% H_2O at 298 K.

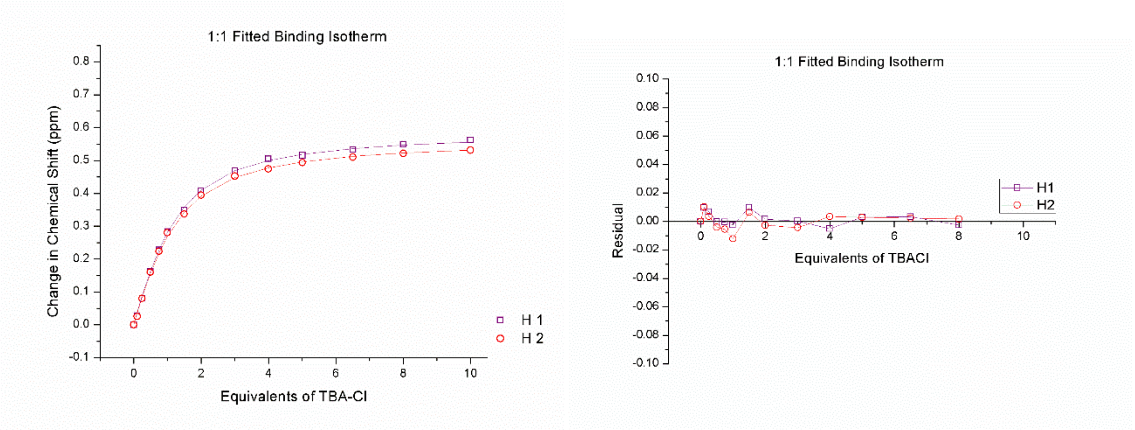

Figure 61. Fitted binding isotherm of n-pentyl tren + TBACl showing the change in chemical shift of the NH protons fitted to the 1:1 binding model (left). K_a = 346 M^{-1} Residual plot showing the random error obtained from the binding isotherm fitting (right).

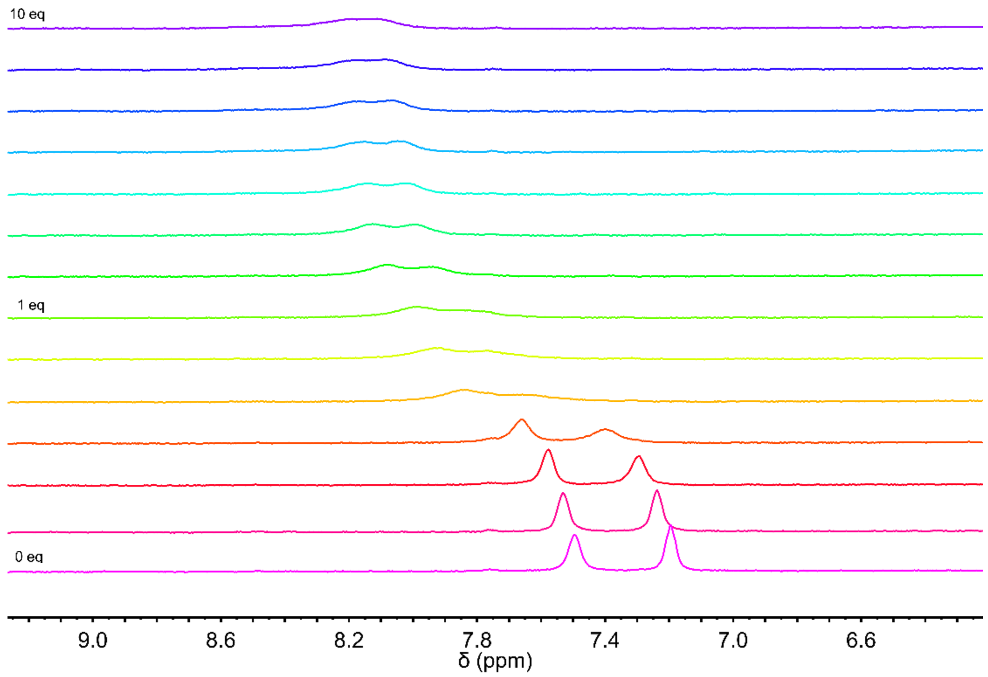

Figure 62. ^{1}H NMR titration spectra as a stack plot for n-pentyl tren (1 mM) + $TEAHCO_3$ in DMSO-d_6/o.5% H_2O at 298 K.

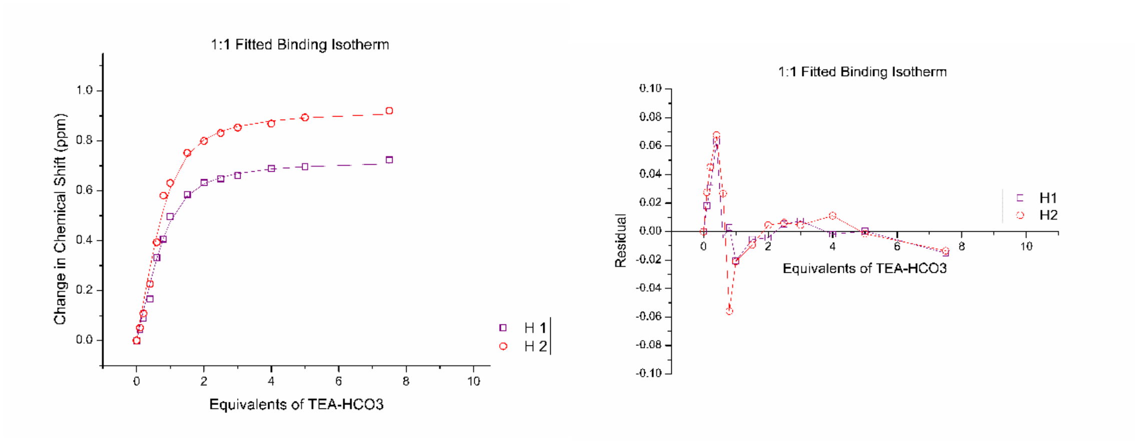

Figure 63. Fitted binding isotherm of n-pentyl tren + $TEAHCO_3$ showing the change in chemical shift of the NH protons fitted to the 1:1 binding model (left). K_a = 5010 M^{-1} Fitted to 7.5 eq. due to peaks broadening and merging at 10 eq. Residual plot showing the random error obtained from the binding isotherm fitting (right).

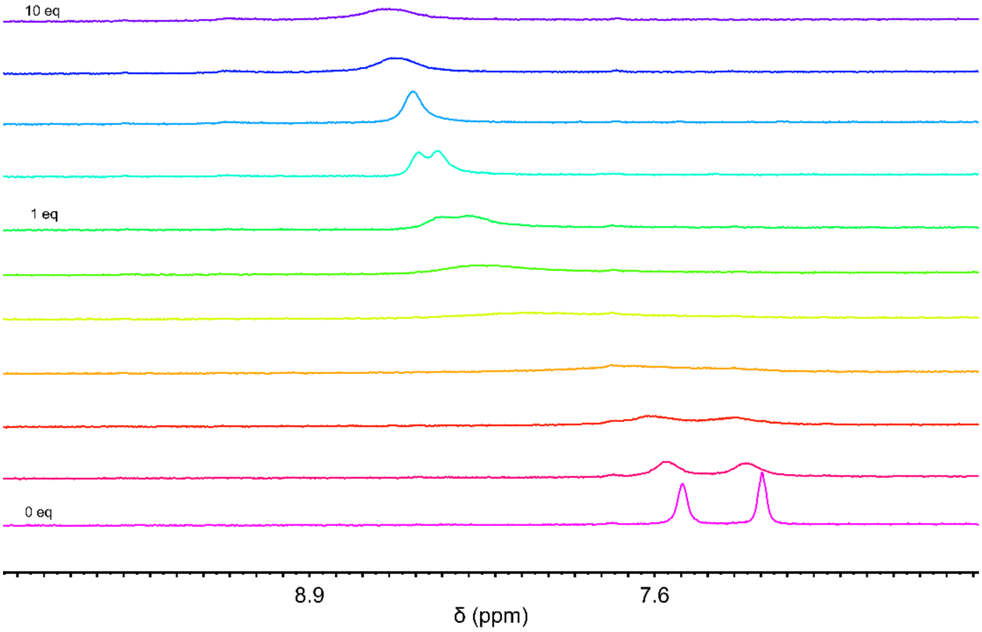

Figure 64. ^{1}H NMR titration spectra as a stack plot for n-pentyl tren (1 mM) + $TBAH_2PO_4$ in DMSO-d_6/o.5% H_2O at 298 K. Peaks broaden between 0 – 1 eq. due to strong binding, then between 1 – 4 eq. peaks sharpen due to formation of the 1:1 complex, above 4 eq. peaks broaden showing the formation of higher order complexes. Data cannot be fitted to any binding models.

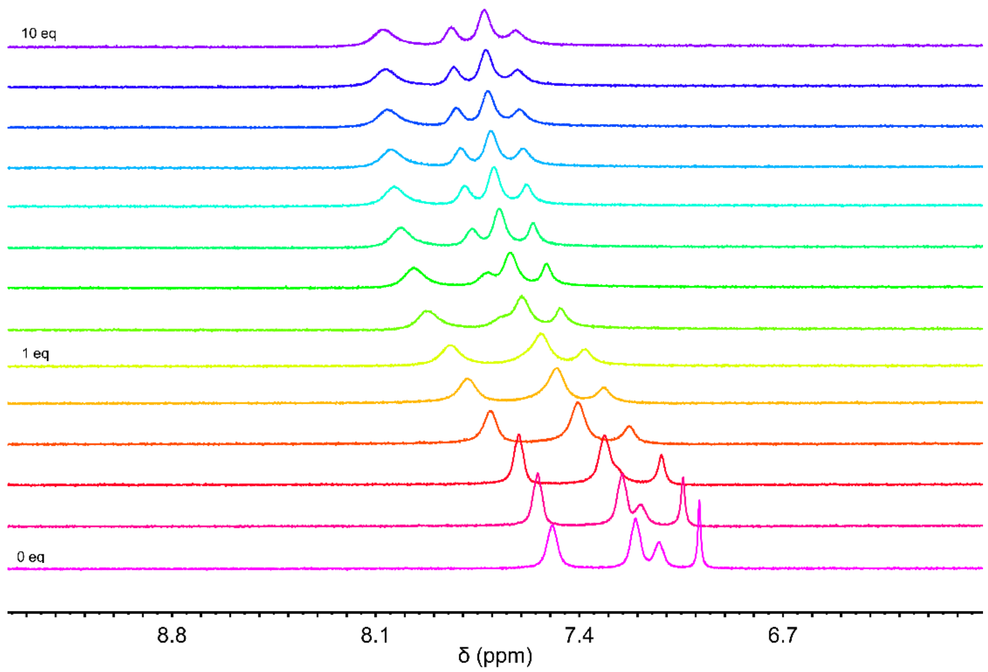

Figure 65. ^{1}H NMR titration spectra as a stack plot for di-n-pentyl mono-t-pentyl tren (5 mM) + TBACl in DMSO-d_6/o.5% H_2O at 298 K.

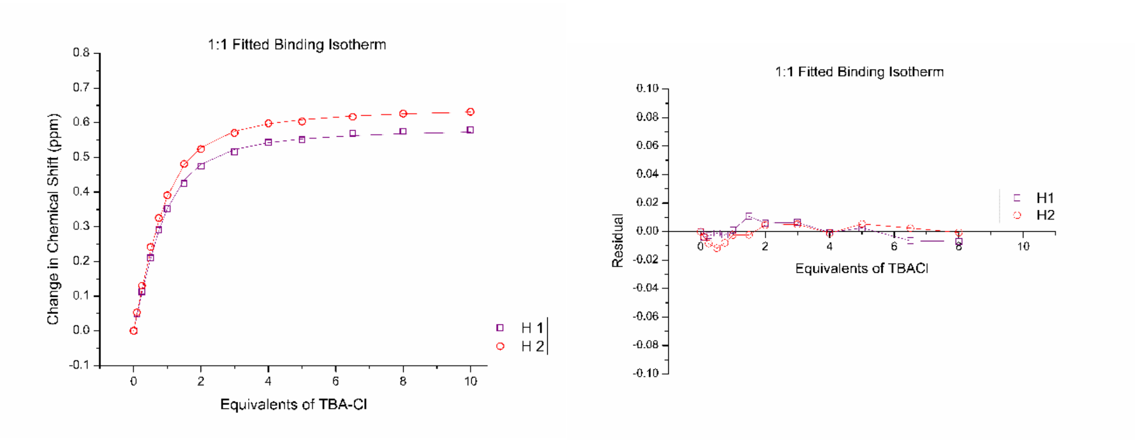

Figure 66. Fitted binding isotherm of di-n-pentyl mono-t-pentyl tren + TBACl showing the change in chemical shift of two NH protons fitted to the 1:1 binding model (left). K_a = 770 M^{-1} Residual plot showing the random error obtained from the binding isotherm fitting (right).

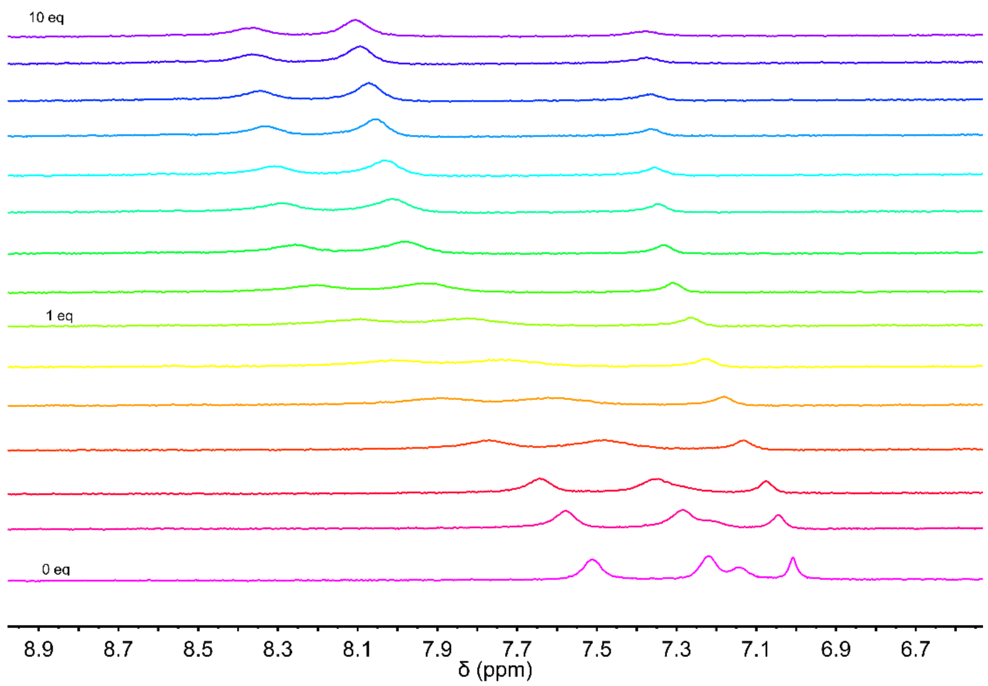

Figure 67. ^{1}H NMR titration spectra as a stack plot for di-n-pentyl mono-t-pentyl tren (1mM) + $TEAHCO_3$ in DMSO-d_6/0.5% H_2O at 298 K.

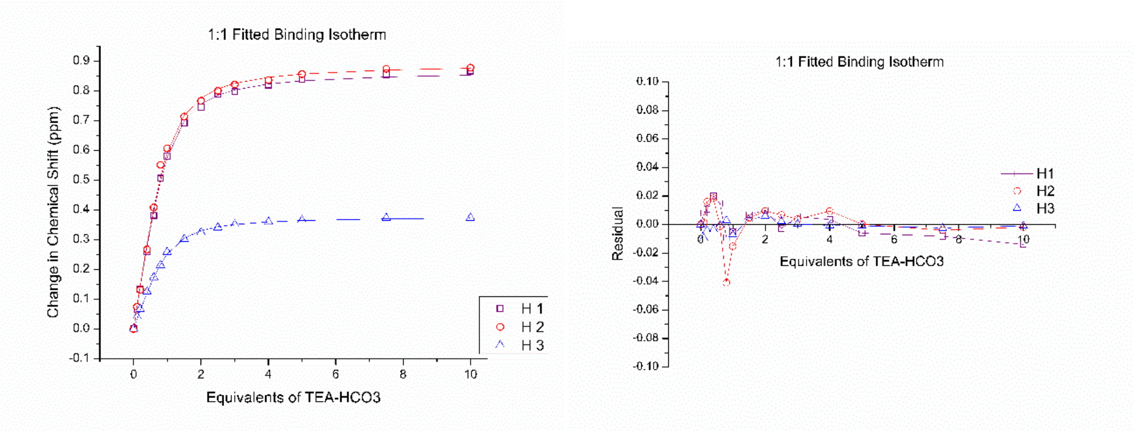

Figure 68. Fitted binding isotherm of di-n-pentyl mono-t-pentyl tren + $TEAHCO_3$ showing the change in chemical shift of three NH protons fitted to the 1:1 binding model (left). K_a = 5560 M^{-1} Residual plot showing the random error obtained from the binding isotherm fitting (right).

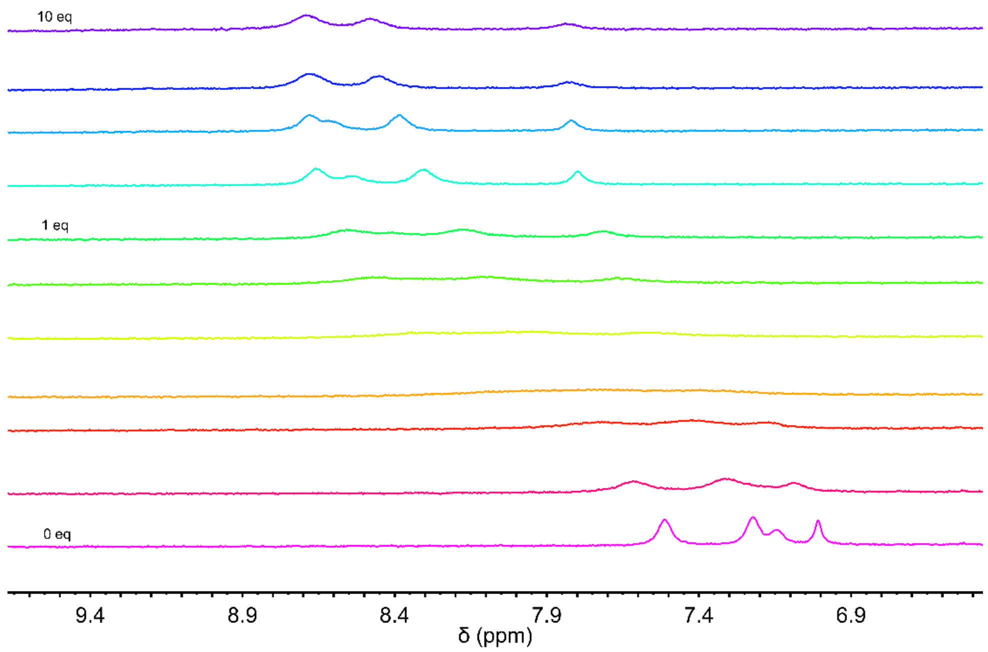

Figure 69. ^{1}H NMR titration spectra as a stack plot for di-n-pentyl mono-t-pentyl tren (1 mM) + $TBAH_2PO_4$ in DMSO-d_6/o.5% H_2O at 298 K. Peaks broaden between 0 – 1 eq. due to strong binding, then between 1 – 10 eq. peaks sharpen due to formation of the 1:1 complex. Data cannot be fitted to any binding models.

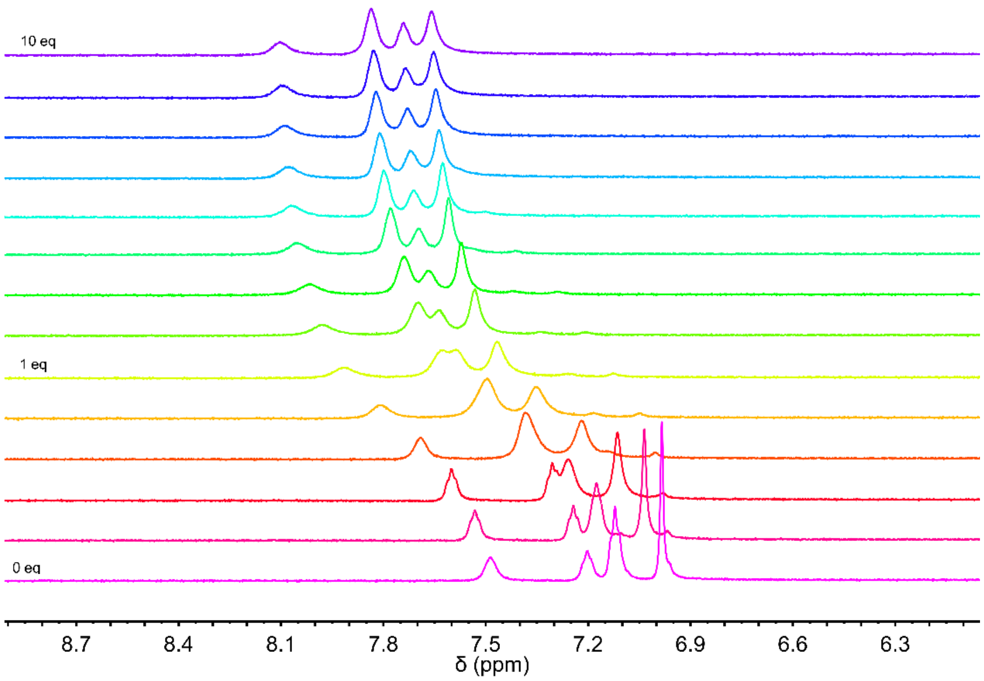

Figure 70. ^{1}H NMR titration spectra as a stack plot for mono-n-pentyl di-t-pentyl tren (5 mM) + TBACl in DMSO-d_6/o.5% H_2O at 298 K.

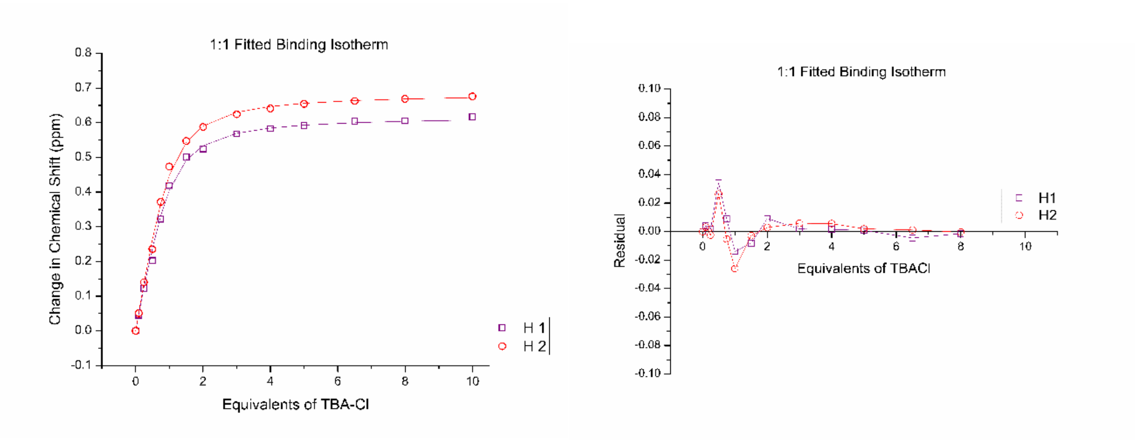

Figure 71. Fitted binding isotherm of mono-n-pentyl di-t-pentyl + TBACl showing the change in chemical shift of two NH protons fitted to the 1:1 binding model (left). K_a = 1080 M^{-1} Residual plot showing the random error obtained from the binding isotherm fitting (right).

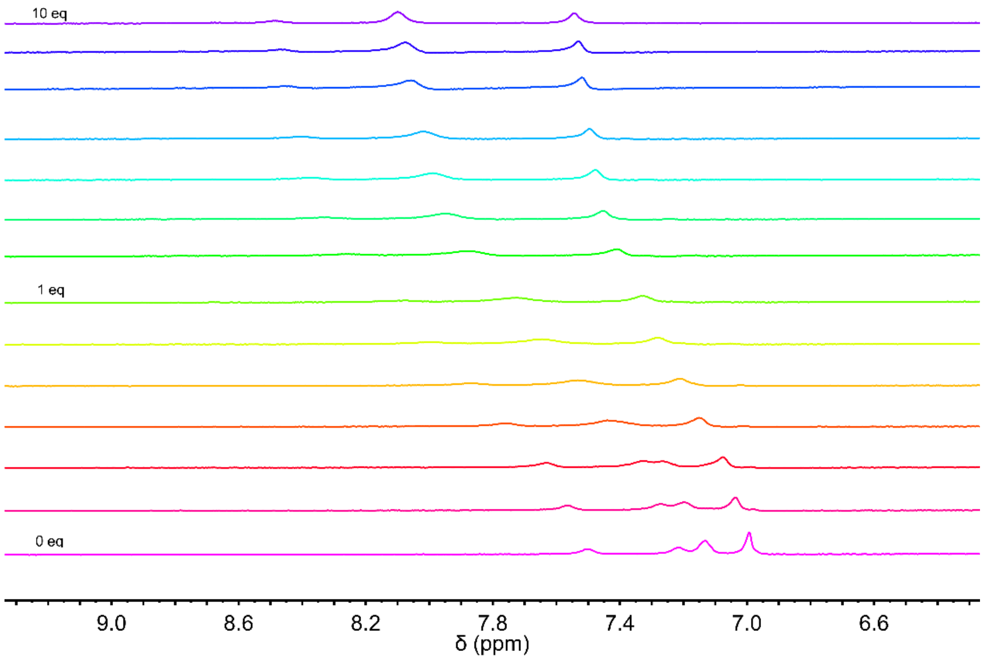

Figure 72. ^{1}H NMR titration spectra as a stack plot for mono-n-pentyl di-t-pentyl tren (1mM) + $TEAHCO_3$ in DMSO-d_6/o.5% H_2O at 298 K.

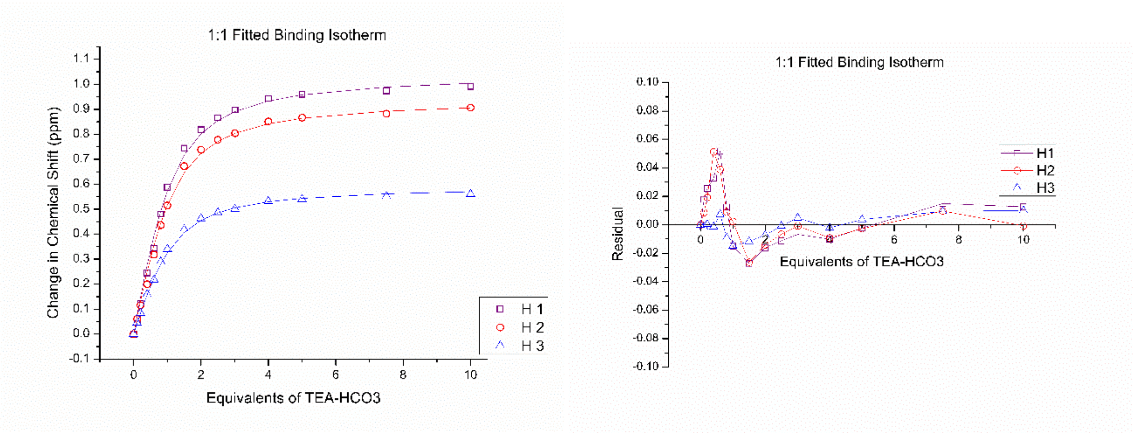

Figure 73. Fitted binding isotherm of mono-n-pentyl di-t-pentyl tren + $TEAHCO_3$ showing the change in chemical shift of three NH protons fitted to the 1:1 binding model (left). K_a = 4930 M^{-1} Residual plot showing the random error obtained from the binding isotherm fitting (right).

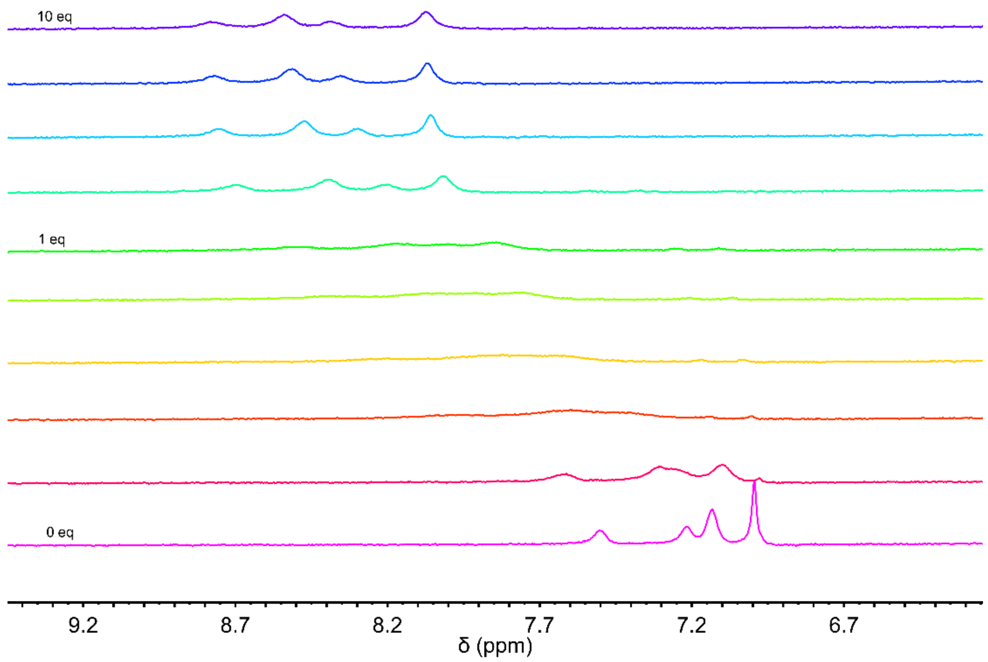

Figure 74. ^{1}H NMR titration spectra as a stack plot for mono-n-pentyl di-t-pentyl tren (1 mM) + $TBAH_2PO_4$ in DMSO-d_6/o.5% H_2O at 298 K. Peaks broaden between 0 – 1 eq. due to strong binding, then between 2 – 10 eq. peaks sharpen due to formation of the 1:1 complex. Data cannot be fitted to any binding models.

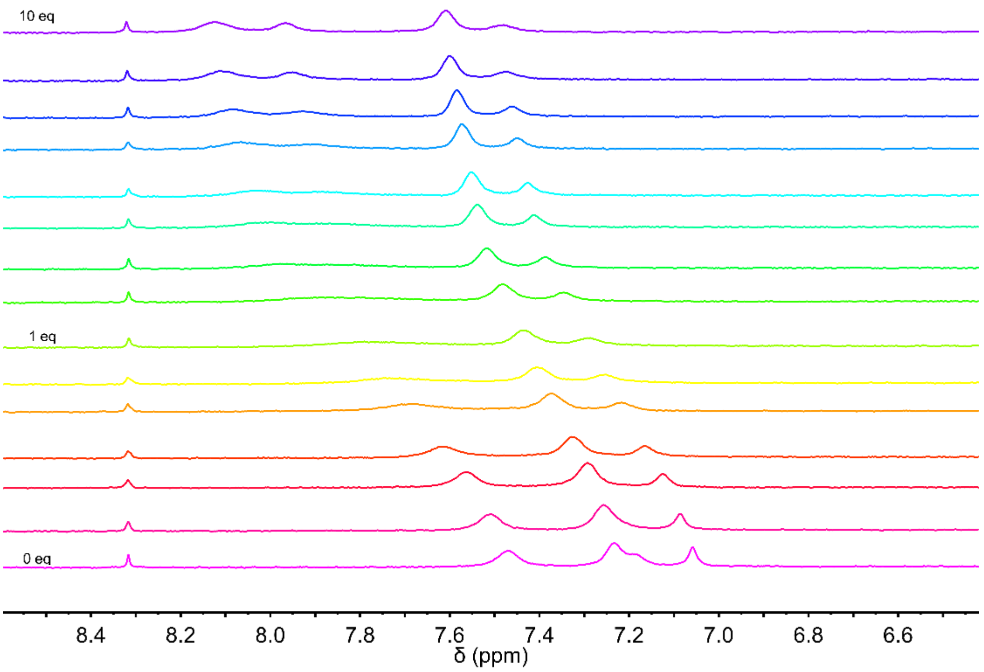

Figure 75. ^{1}H NMR titration spectra as a stack plot for macrocycle tren (1 mM) + TBACl in DMSO-d_6/o.5% H_2O at 298 K.

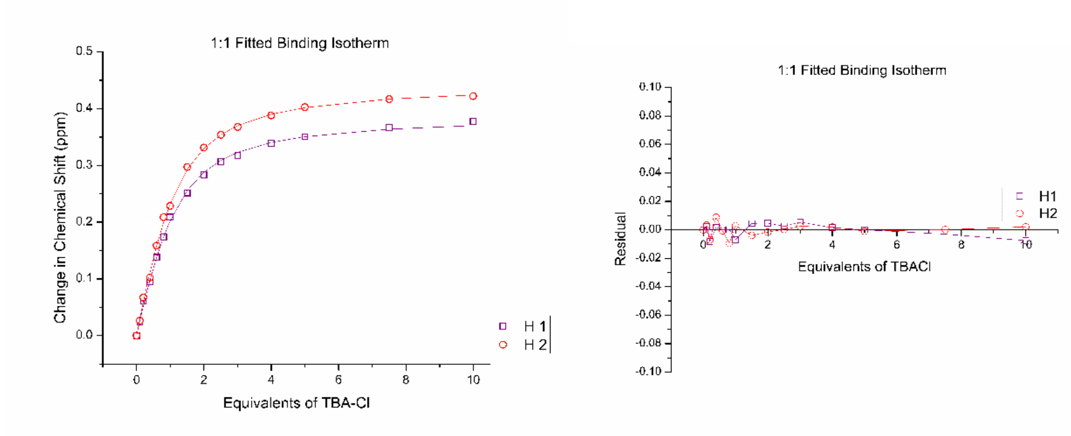

Figure 76. Fitted binding isotherm of macrocycle tren + TBACl showing the change in chemical shift of two NH protons fitted to the 1:1 binding model (left). K_a = 2170 M^{-1} Residual plot showing the random error obtained from the binding isotherm fitting (right).

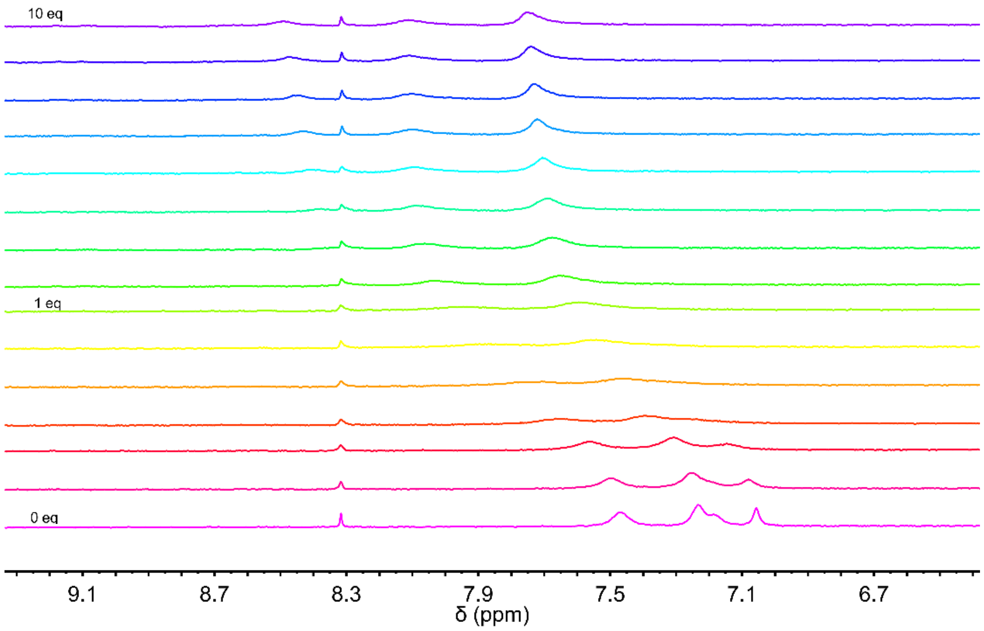

Figure 77. ^{1}H NMR titration spectra as a stack plot for macrocycle tren (1 mM) + $TEAHCO_3$ in DMSO-d_6/o.5% H_2O at 298 K.

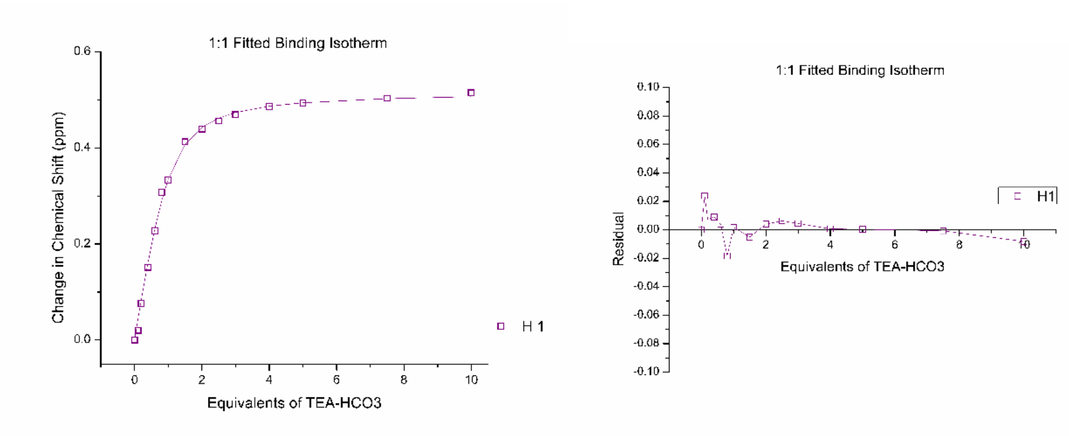

Figure 78. Fitted binding isotherm of macrocycle tren + $TEAHCO_3$ showing the change in chemical shift of one NH proton fitted to the 1:1 binding model (left). K_a = 4930 M^{-1} Residual plot showing the random error obtained from the binding isotherm fitting (right).

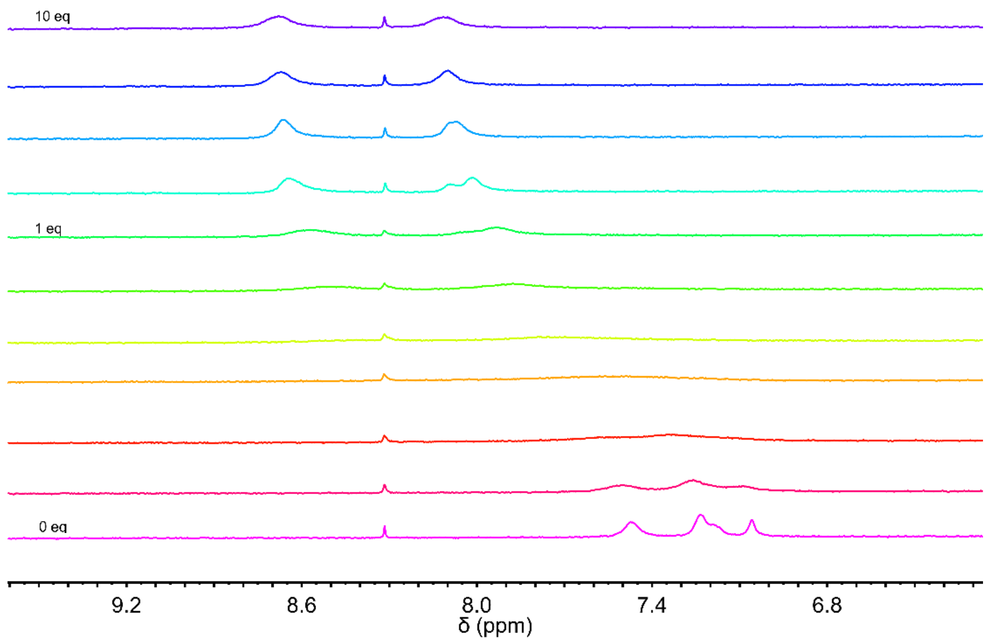

Figure 79. ^{1}H NMR titration spectra as a stack plot for macrocycle tren (1 mM) + $TBAH_2PO_4$ in DMSO-*d*$_6$/o.5% H_2O at 298 K. Peaks broaden between 0 – 1 eq. due to strong binding, then between 1 – 10 eq. peaks sharpen due to formation of the 1:1 complex. Data cannot be fitted to any binding models.

1.1.3 Cationophore Coupled Assay

Unilamellar vesicles were prepared using previously reported literature procedures. A lipid film of POPC (1-palmitoyl-2-oleoyl-sn-glycero-3-phosphocholine) was prepared from a chloroform solution under reduced pressure and then dried under vacuum for 4 or more hours. The lipid film was rehydrated by vortexing with an internal solution of HEPES buffered potassium chloride (KCl) at pH 7.2. The lipid suspension was then subjected to 9 freeze-thaw cycles and left to rest at room temperature for 30 minutes. After this the suspension was extruded 25 times through a 200 nm polycarbonate membrane resulting in unilamellar vesicles of an average diameter of 200 nm. The vesicles were then passed through a sephadex column saturated with HEPES buffered external potassium gluconate (KGlu) at pH 7.2, which allowed the exchange of any unencapsulated KCl for KGlu. The lipid solution obtained after sephadex was diluted to a standard volume (usually 10 mL) with the external KGlu solution to obtain a lipid stock of known concentration.

For each measurement the lipid stock solution was diluted with the external buffered solution to a standard volume (5.0 mL) with a lipid concentration of 1.0 mM. Where a cationophore (valinomycin or monensin) was used, a 1 mol% DMSO solution of the cationophore (0.5 mM, 10 μL) was added to the lipid solution first. Then the addition of the test compound as a DMSO solution (10 μL) was added to start the experiment and the chloride efflux was monitored using a chloride selective electrode. After 5 minutes detergent (50 μL) was added to lyse the vesicles and the 100% chloride efflux reading was taken at 7 minutes.

The screening was performed with the transporter alone, the transporter plus valinomycin or the transporter plus monensin, to assess the transport mechanism in different vesicle conditions. Transport processes coupled to valinomycin show electrogenic character and transport processes coupled to monensin show electroneutral character. Receptor concentration depended upon transporter activity and is included in the legend for each assay.

Percentage chloride efflux over time for receptors **1-6** tested in the cationophore coupled assay.

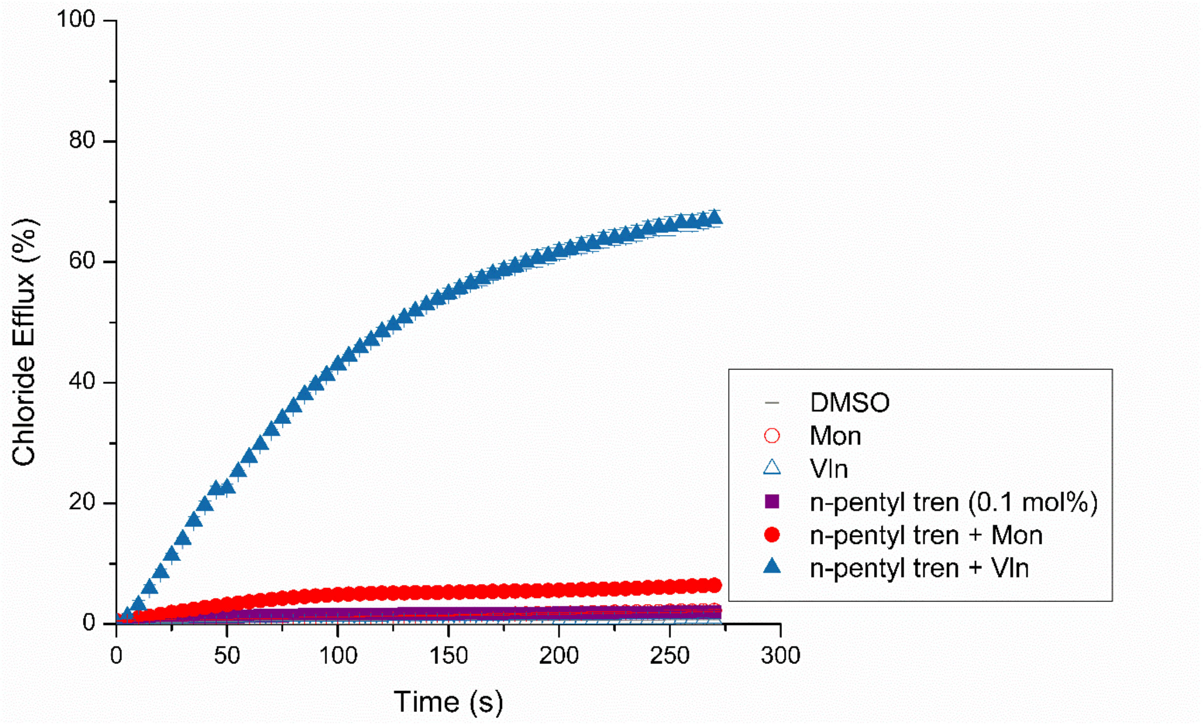

Figure 80. Chloride efflux facilitated by **1** (0.1 mol%) in the presence of valinomycin (blue), monensin (red) and alone (purple) in the cationophore coupled assay.

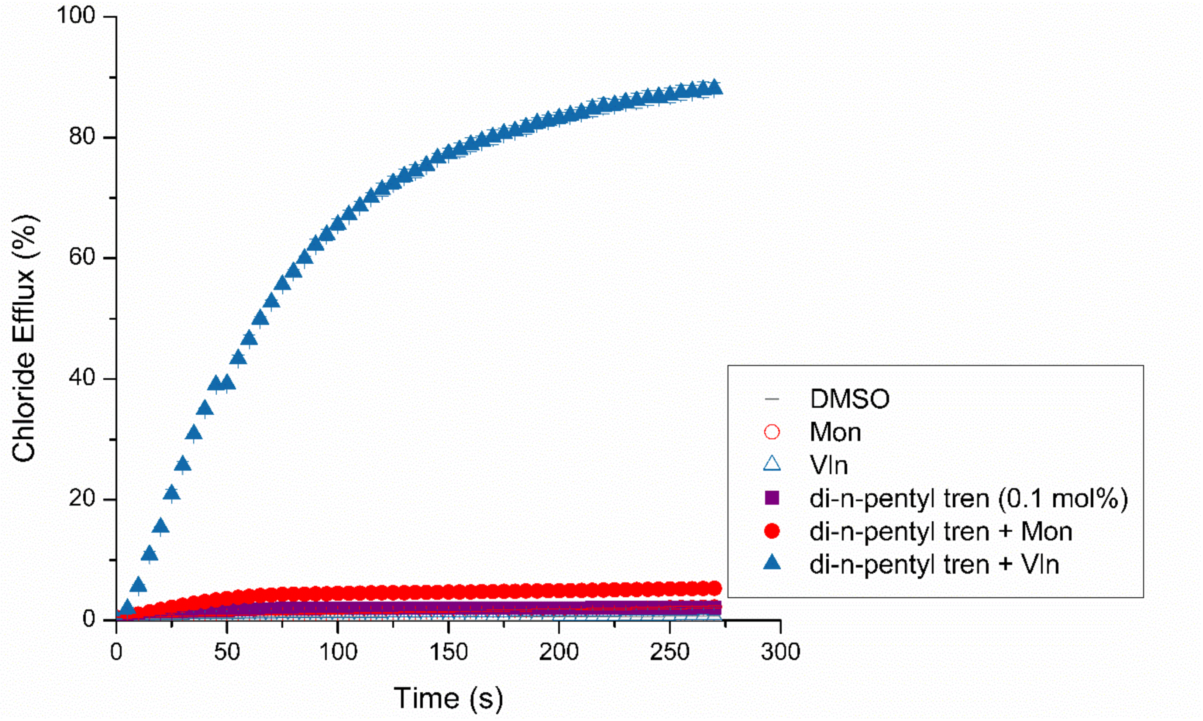

Figure 81. Chloride efflux facilitated by **2** (0.1 mol%) in the presence of valinomycin (blue), monensin (red) and alone (purple) in the cationophore coupled assay.

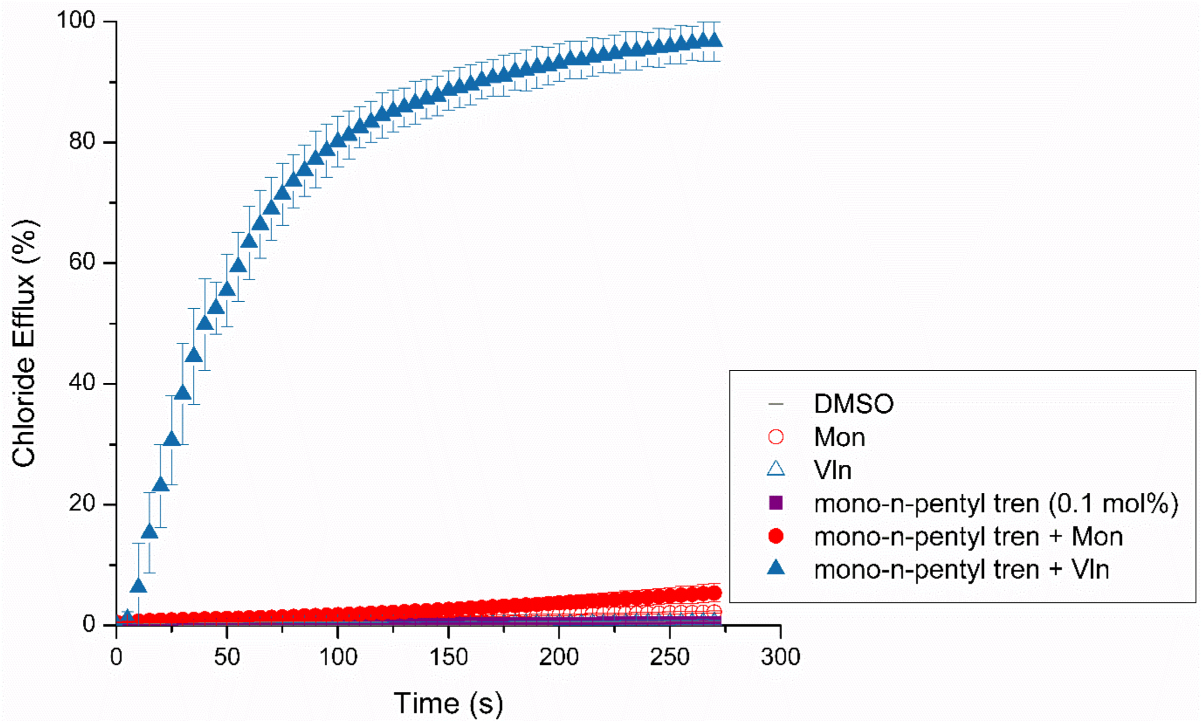

Figure 82. Chloride efflux facilitated by **3** (0.1 mol%) in the presence of valinomycin (blue), monensin (red) and alone (purple) in the cationophore coupled assay.

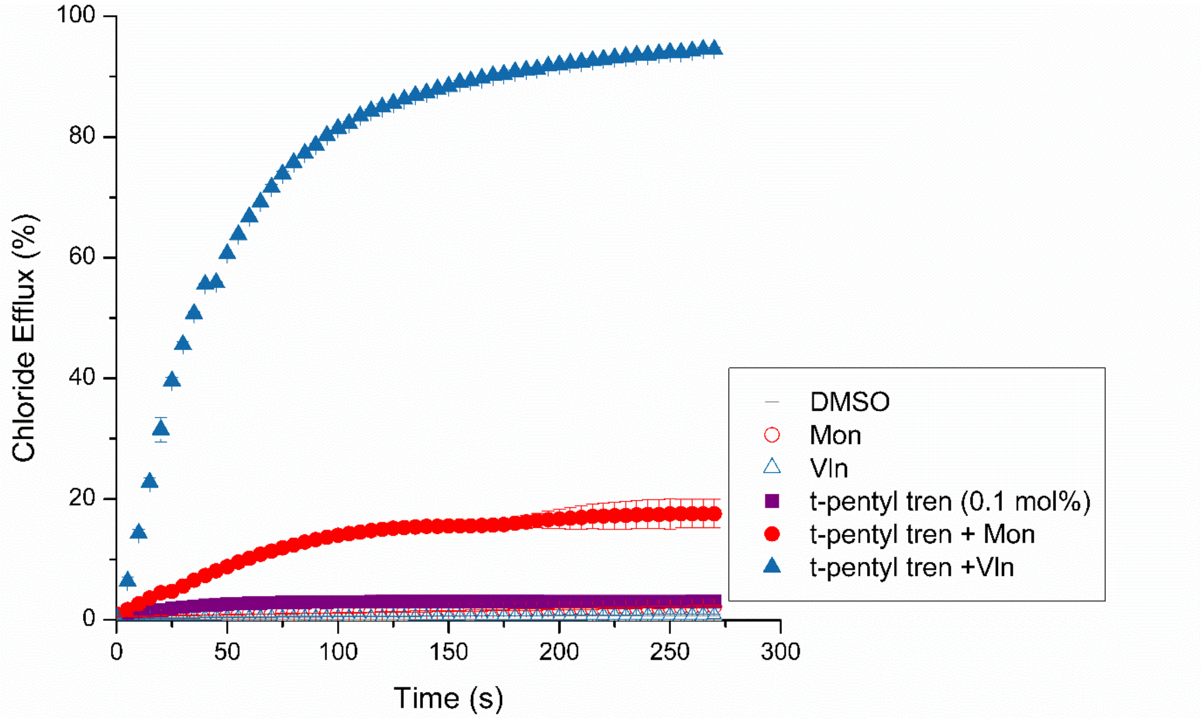

Figure 83. Chloride efflux facilitated by **4** (0.1 mol%) in the presence of valinomycin (blue), monensin (red) and alone (purple) in the cationophore coupled assay.

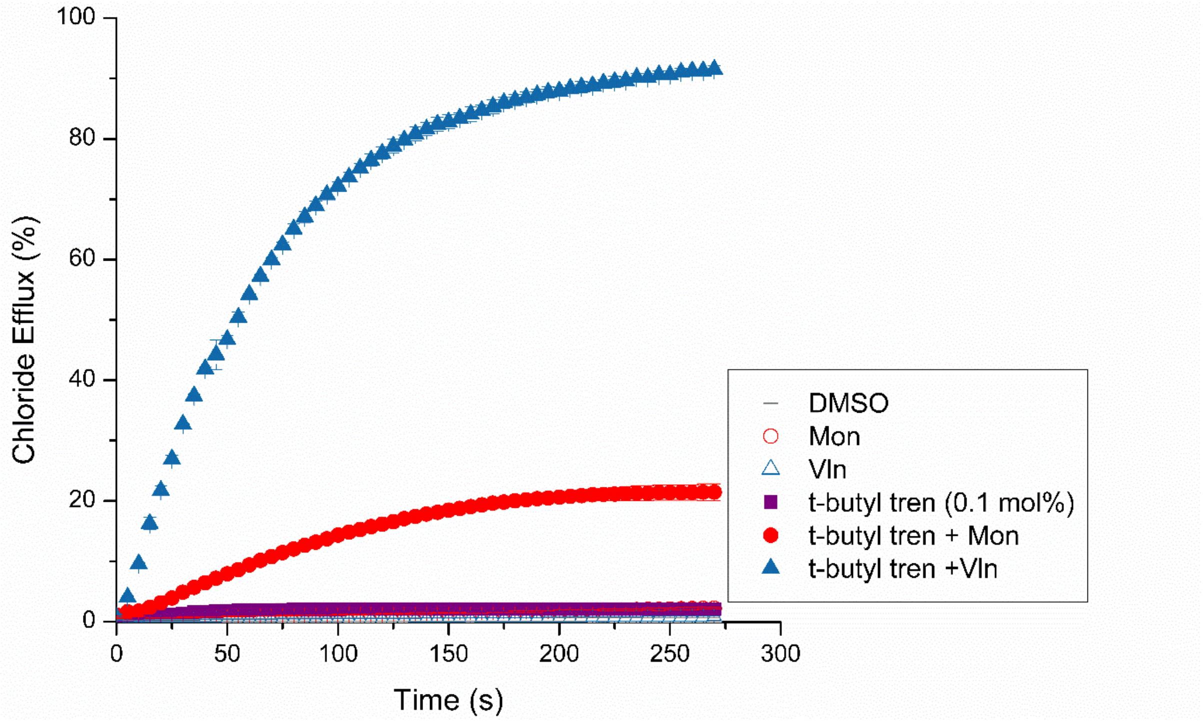

Figure 84. Chloride efflux facilitated by **5** (0.1 mol%) in the presence of valinomycin (blue), monensin (red) and alone (purple) in the cationophore coupled assay.

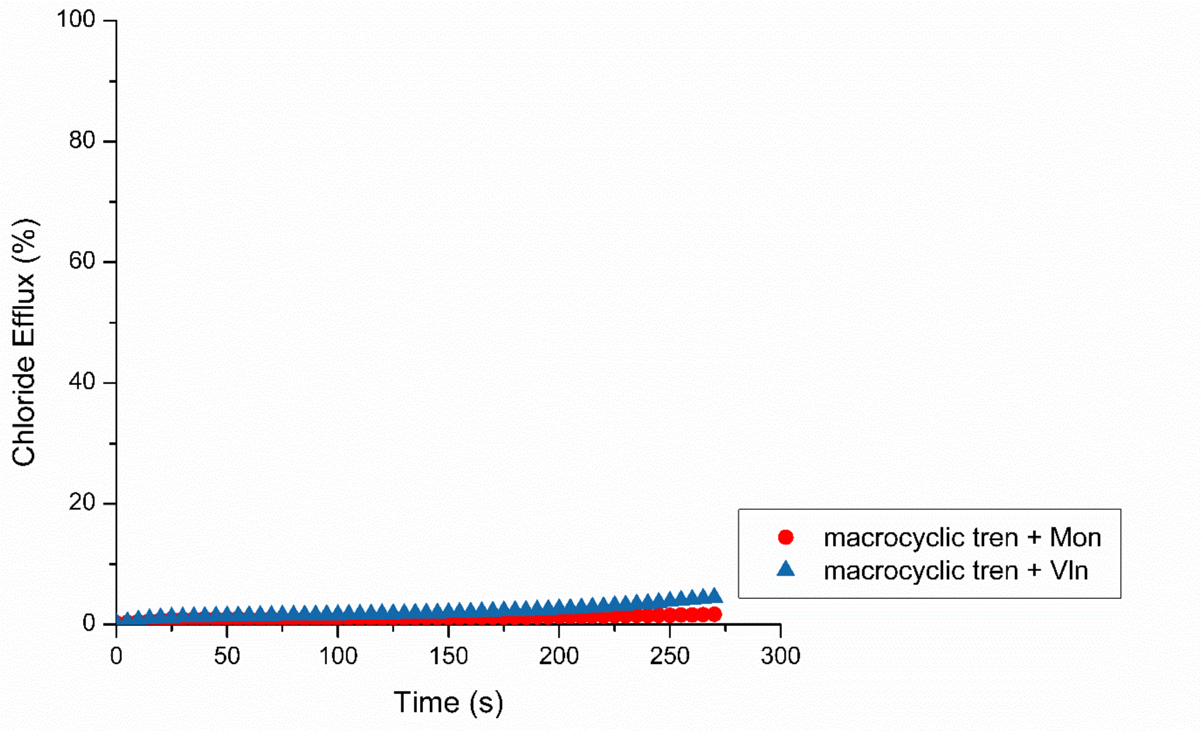

Figure 85. Chloride efflux facilitated by **6** (0.1 mol%) in the presence of valinomycin (blue), monensin (red) and alone (purple) in the cationophore coupled assay.

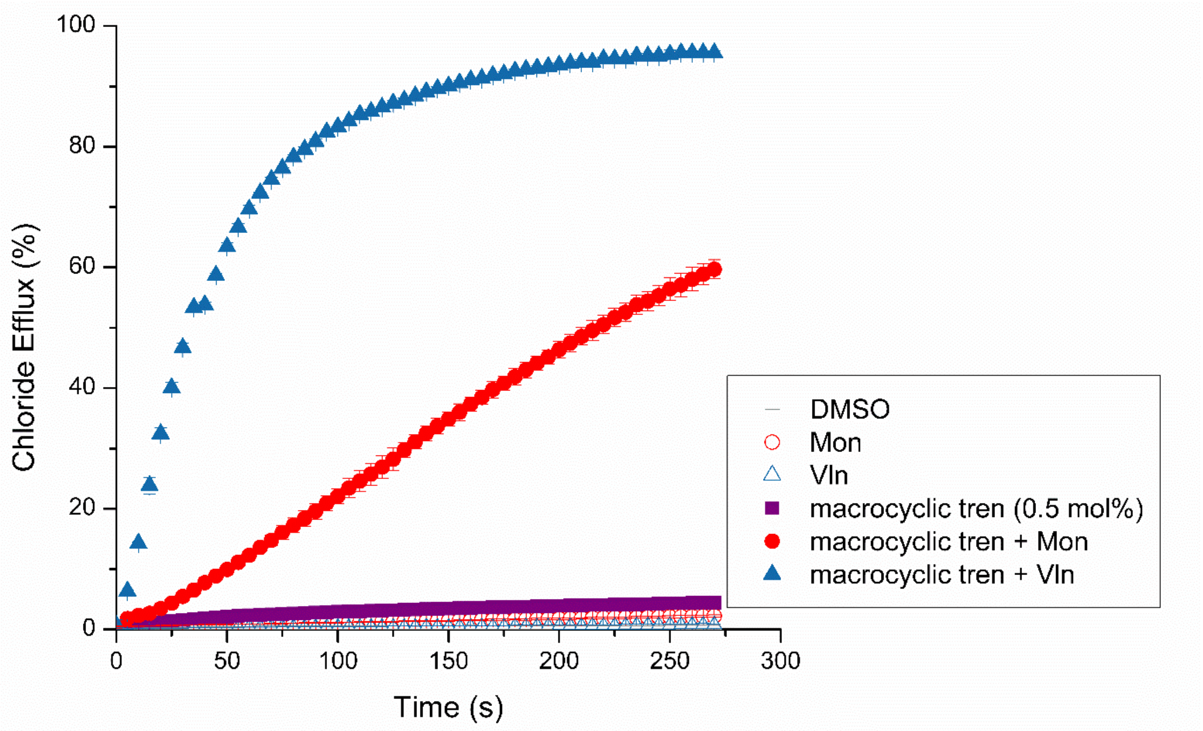

Figure 86. Chloride efflux facilitated by **6** (0.5 mol%) in the presence of valinomycin (blue), monensin (red) and alone (purple) in the cationophore coupled assay.

1.1.4 NMDG-Cl Assay

Unilamellar vesicles were prepared using previously reported literature procedures. A lipid film of POPC (1-palmitoyl-2-oleoyl-sn-glycero-3-phosphocholine) was prepared from a chloroforms olution under reduced pressure and then dried under vacuum for 4 or more hours. The lipid film was rehydrated by vortexing with an internal solution of NMDG-Cl (100 mM) containing HPTS (1 mM) buffered to pH 7 with HEPES (10 mM). The lipid suspension was then subjected to 9 freeze-thaw cycles and left to rest at room temperature for 30 minutes. After this, the suspension was extruded 25 times through a 200 nm polycarbonate membrane resulting in unilamellar vesicles of a mean diameter of 200 nm. The un-encapsulated HPTS was removed by size exclusion chromatography using a sephadex G-25 column and an external solution eluent that does not contain HPTS. The lipid solution obtained after sephadex was diluted to a standard volume (usually 5 mL) with the NMDG-Cl (100 mM) buffered to pH 7 with HEPES, external solution to obtain a lipid stock of known concentration. Where required, BSA treatment of vesicles was performed on the whole lipid stock by adding 1 mol% (with respect to lipid) to the lipid solution and leaving to stir for 30 minutes.

The lipid stock was diluted with the external buffer solution to a standard volume (2.5 mL), to afford a solution with a lipid concentration of 0.1 mM. The compounds were added as a DMSO solution (5 μL) and when an assisting ionophore (gramicidin) or oleic acid was used this was added before the transporter and added as a DMSO solution (5 μL, 0.1 mol% or 2 mol% respectively (with respect to lipid)). To start the experiment, after the addition of the compounds, a NMDG base pulse (25 μL, 0.5 M) was spiked in to generate a pH gradient across the membrane. After 200 s, detergent (50 μL of Triton X-100 (11 w%) in H_2O:DMSO (7:1 v/v)) was added to lyse the vesicles and after 5 mins a final reading was taken. This value represented 100 % and was used for calibration. The fractional fluorescence intensity (I_f) was calculated using:

$$I_f = \frac{R_t - R_0}{R_d - R_0}$$

where R_t is the fluorescence ratio at time t, R_0 is the fluorescence ratio at time 0 and R_d is the fluorescence ratio at the end of the experiment, after the addition of detergent. The HPTS exchange assay was performed as described above testing the compounds at varying concentrations. From these results the fluorescence ratio at 200 s was plotted as a function of

the transporter concentration (mol%, with respect to lipid). The data points were then fitted to the Hill equation using OriginPro 9.1:

$$y = y_0 + (y_{max} - y_0)\frac{x^n}{k^n + x^n}$$

where y is the I_f at 200 s and x is the transporter concentration (mol %, with respect to lipid). y_0 is the I_f obtained for the blank DMSO run, y_{max} is the maximum I_f value and k and n are the parameters to be fitted. n is the Hill coefficient and k is the EC50.

The rate of pH dissipation through H^+/Cl^- symport (or Cl^-/OH^-antiport) by receptors **1**-**6** is shown by the change in fluorescence intensity over time in three different conditions. 1. BSA treated vesicles – to monitor the H^+/Cl^- symport (or Cl^-/OH^-antiport), 2. In the presence of gramicidin – to monitor the Cl^- uniport, and 3. In the presence of fatty acids – to evaluate the Cl^- selectivity over the contribution to the fatty acid assisted H^+/Cl^- symport. The results of Hill Analysis for each receptor is shown and receptor concentration is included in the legend for each assay.

t-Butyl tren

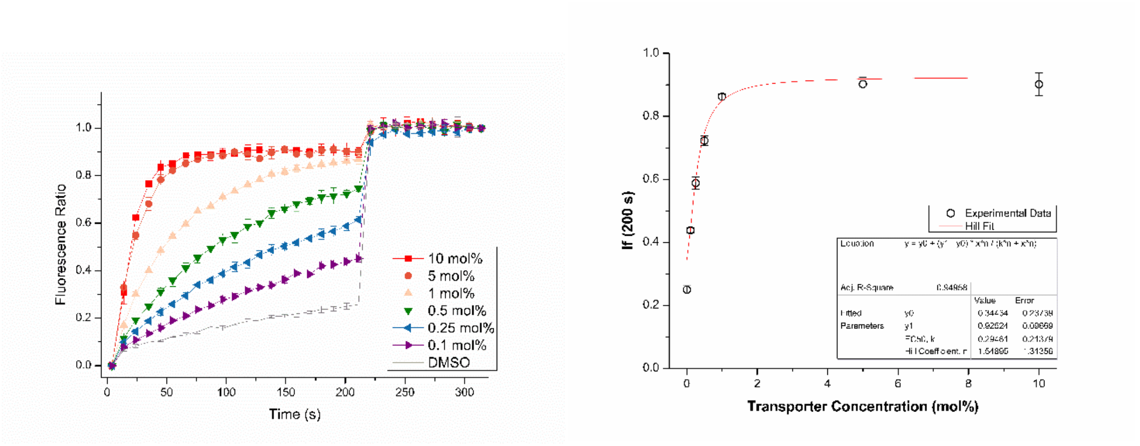

Figure 87. Hill analysis of H^+/Cl^- symport (or Cl^-/OH^- antiport) facilitated by t-butyl tren in the NMDG-Cl assay with ‘fatty acid free conditions’ in BSA treated vesicles.

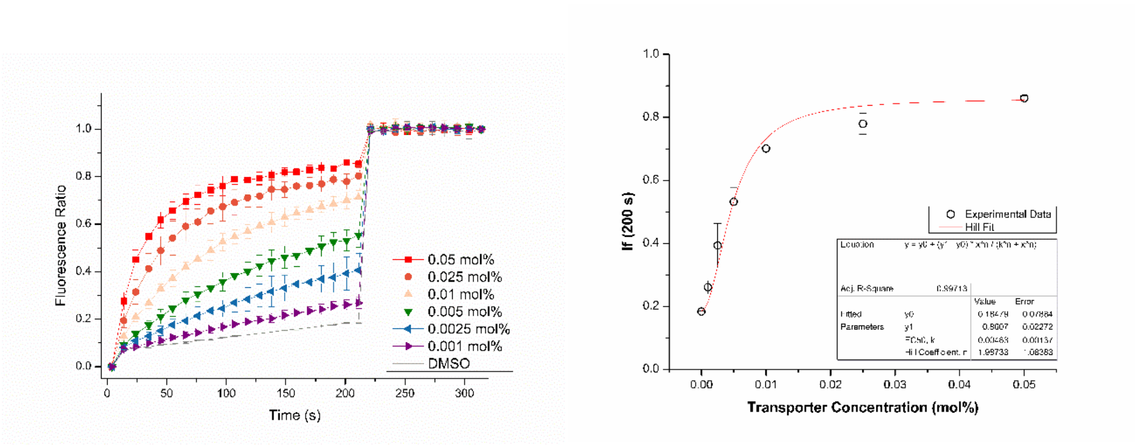

Figure 88. Hill analysis of H^+/Cl^- symport (or Cl^-/OH^- antiport) facilitated by a combination of gramicidin and t-butyl tren in the NMDG-Cl assay.

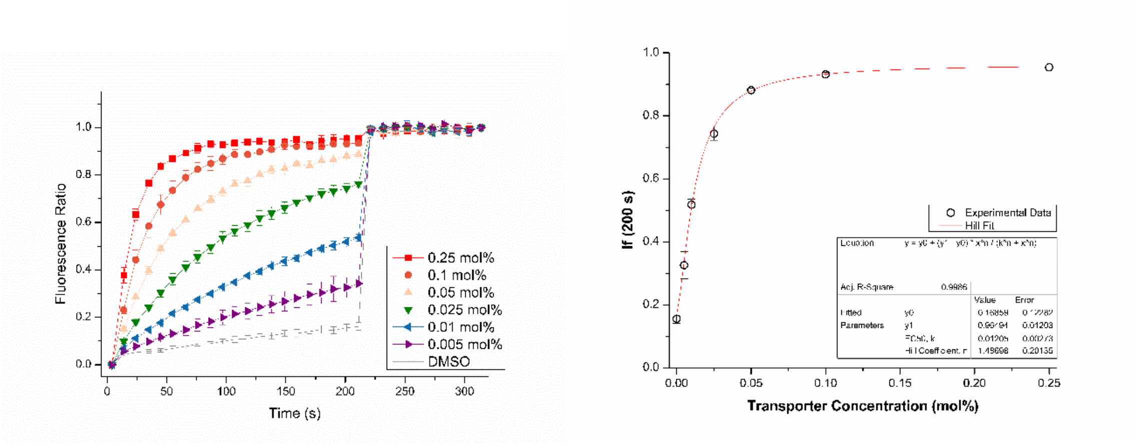

Figure 89. Hill analysis of H^+/Cl^- symport (or Cl^-/OH^- antiport) facilitated by a combination of oleic acid and t-butyl tren in the NMDG-Cl assay.

t-Pentyl tren

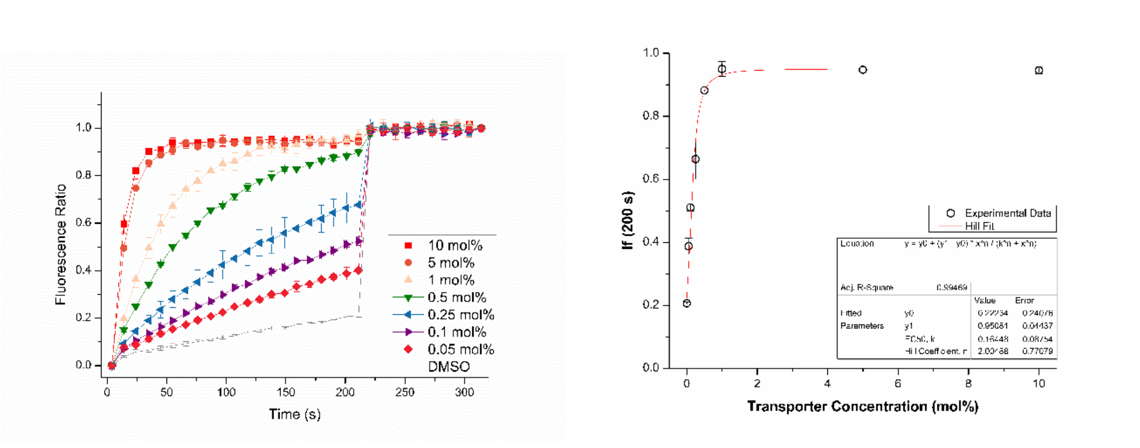

Figure 90. Hill analysis of H^+/Cl^- symport (or Cl^-/OH^- antiport) facilitated by t-pentyl tren in the NMDG-Cl assay with ‘fatty acid free conditions’ in BSA treated vesicles.

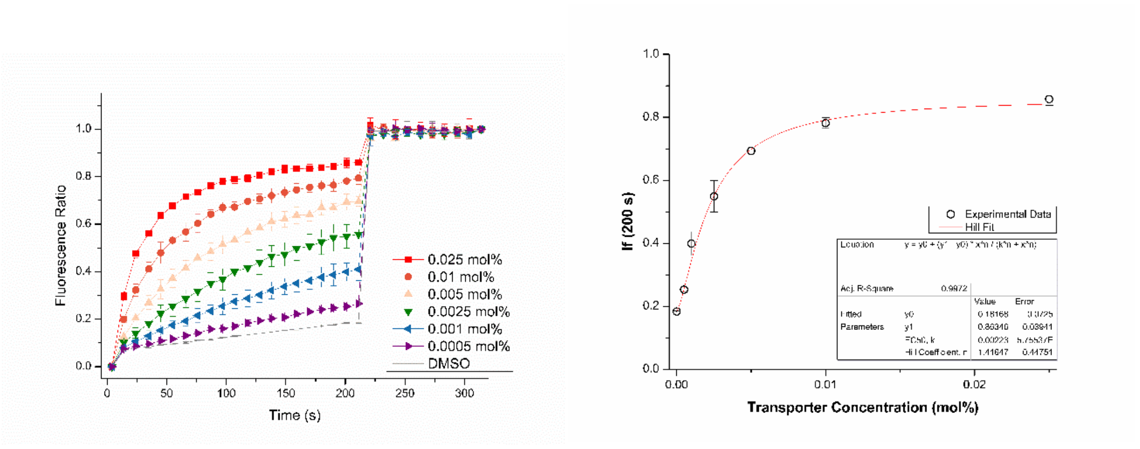

Figure 91. Hill analysis of H^+/Cl^- symport (or Cl^-/OH^- antiport) facilitated by a combination of gramicidin and t-pentyl tren in the NMDG-Cl assay.

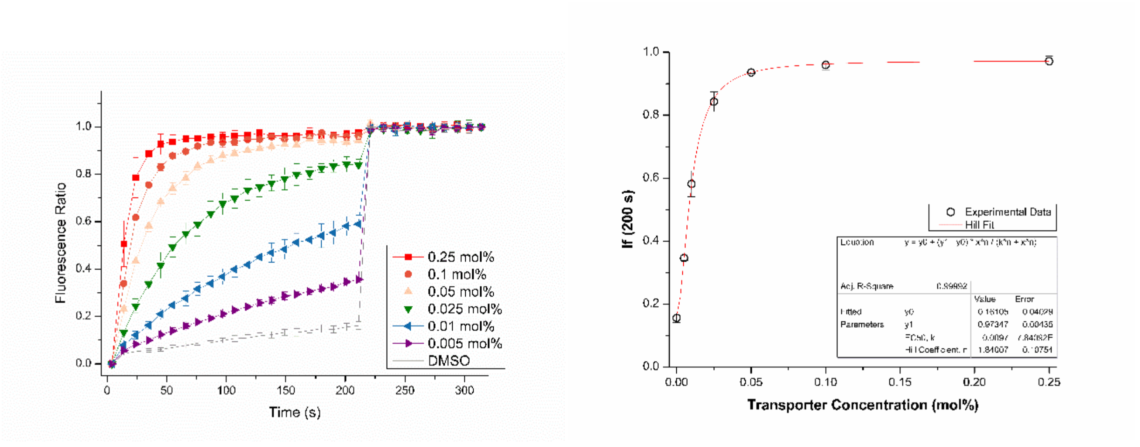

Figure 92. Hill analysis of H^+/Cl^- symport (or Cl^-/OH^- antiport) facilitated by a combination of oleic acid and t-pentyl tren in the NMDG-Cl assay.

n-Pentyl tren

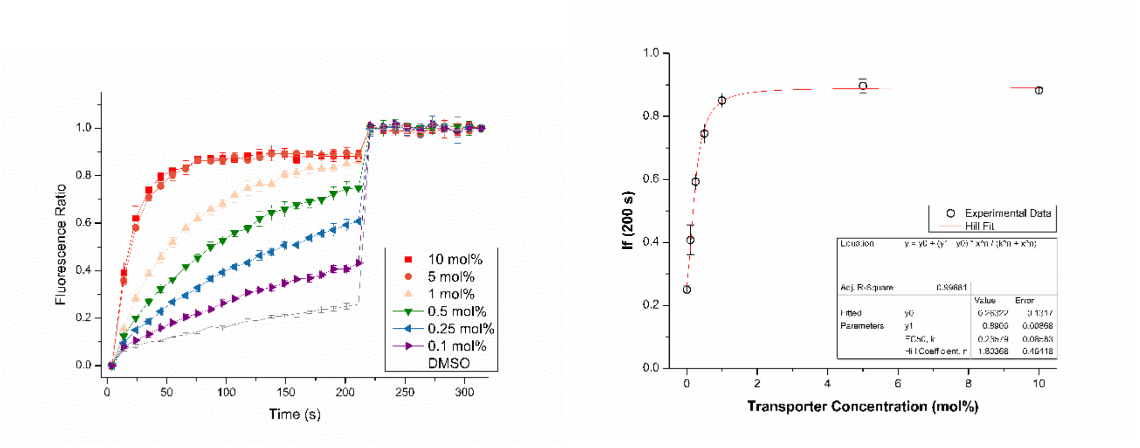

Figure 93. Hill analysis of H^+/Cl^- symport (or Cl^-/OH^- antiport) facilitated by n-pentyl tren in the NMDG-Cl assay with ‘fatty acid free conditions’ in BSA treated vesicles.

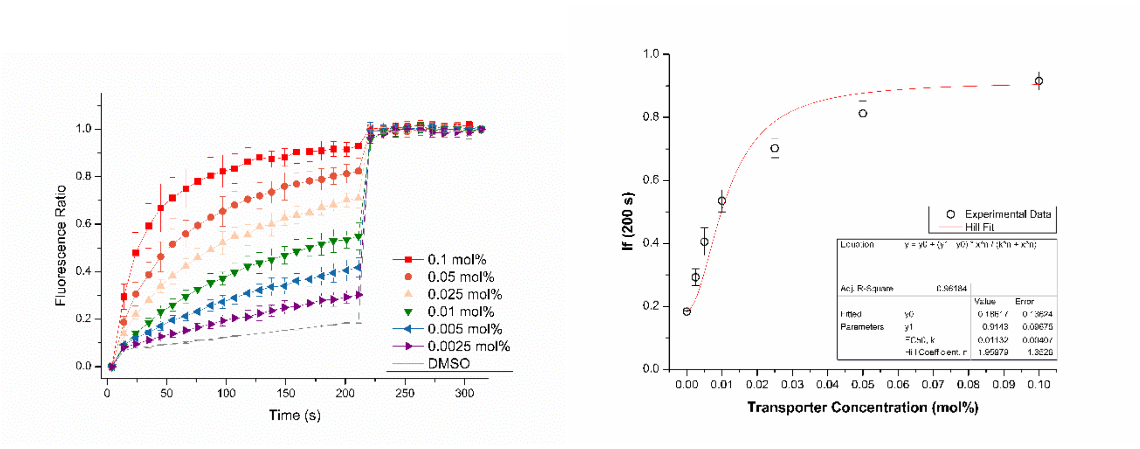

Figure 94. Hill analysis of H^+/Cl^- symport (or Cl^-/OH^- antiport) facilitated by a combination of gramicidin and n-pentyl tren in the NMDG-Cl assay.

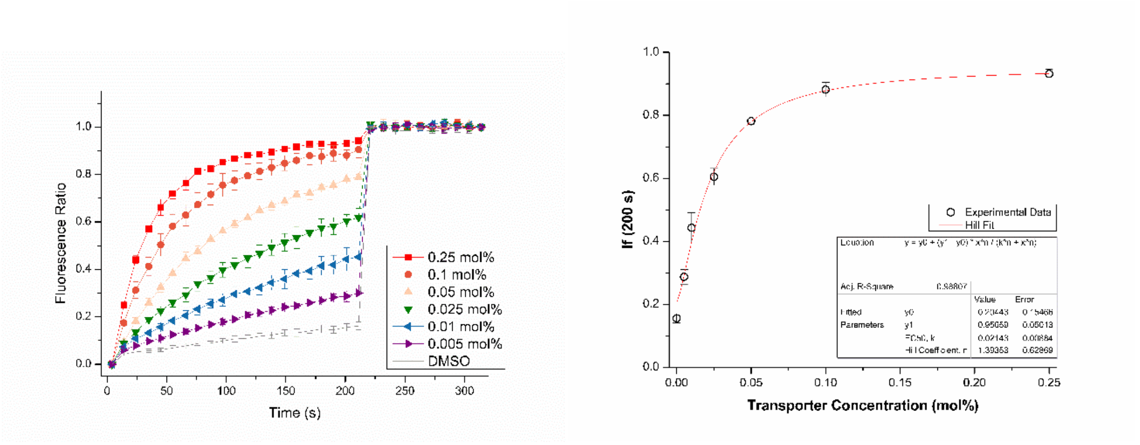

Figure 95. Hill analysis of H^+/Cl^- symport (or Cl^-/OH^- antiport) facilitated by a combination of oleic acid and n-pentyl tren in the NMDG-Cl assay.

Di-n-pentyl mono-t-pentyl tren

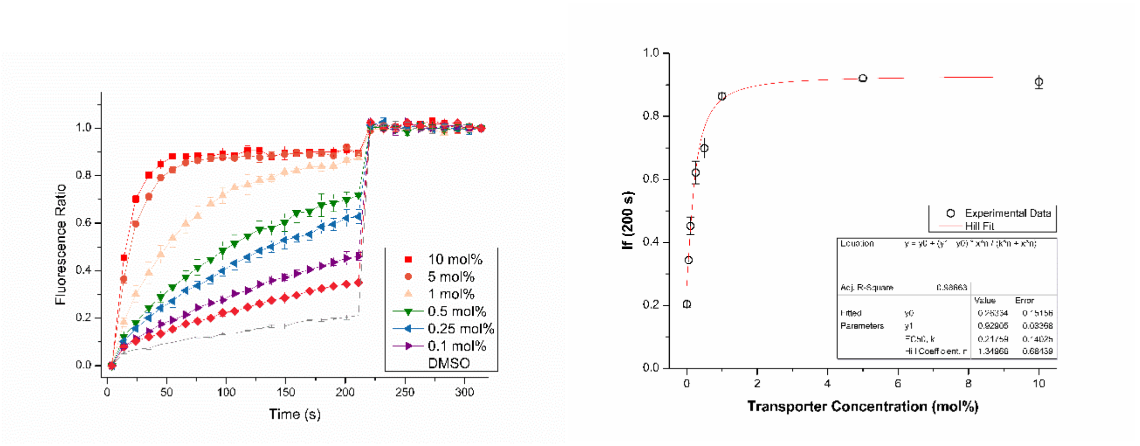

Figure 96. Hill analysis of H^+/Cl^- symport (or Cl^-/OH^- antiport) facilitated by di-n-pentyl mono t-pentyl tren in the NMDG-Cl assay with ‘fatty acid free conditions’ in BSA treated vesicles.

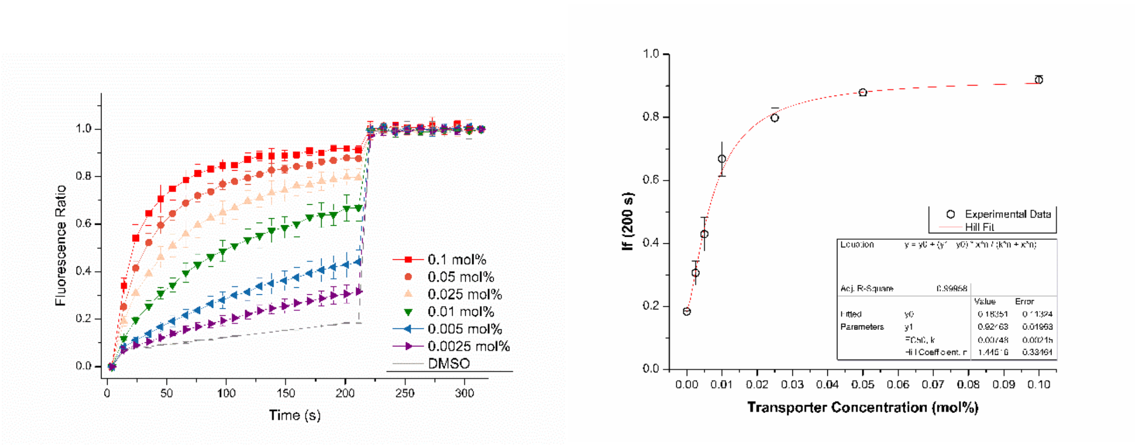

Figure 97. Hill analysis of H^+/Cl^- symport (or Cl^-/OH^- antiport) facilitated by a combination of gramicidin and di-n-pentyl mono-t-pentyl tren in the NMDG-Cl assay.

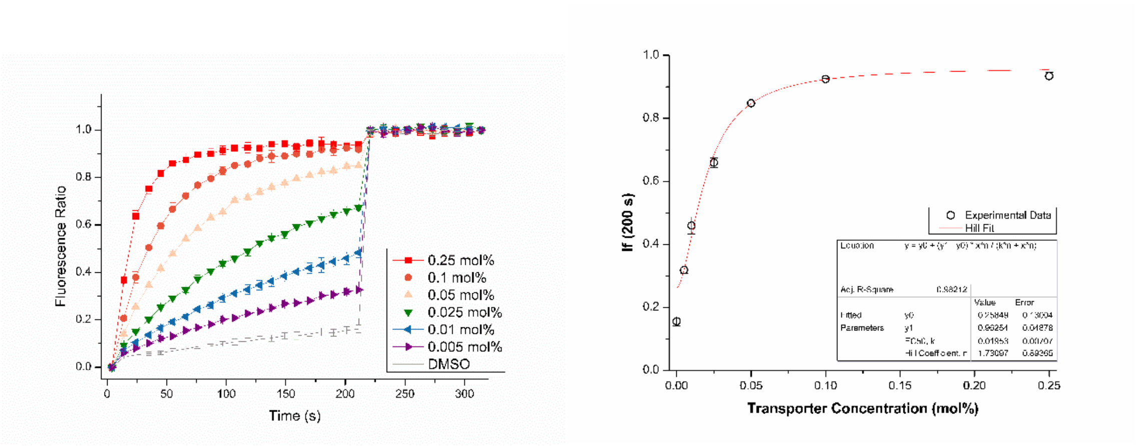

Figure 98. Hill analysis of H^+/Cl^- symport (or Cl^-/OH^- antiport) facilitated by a combination of oleic acid and di-n-pentyl mono-t-pentyl tren in the NMDG-Cl assay.

Mono-n-pentyl di-t-pentyl tren

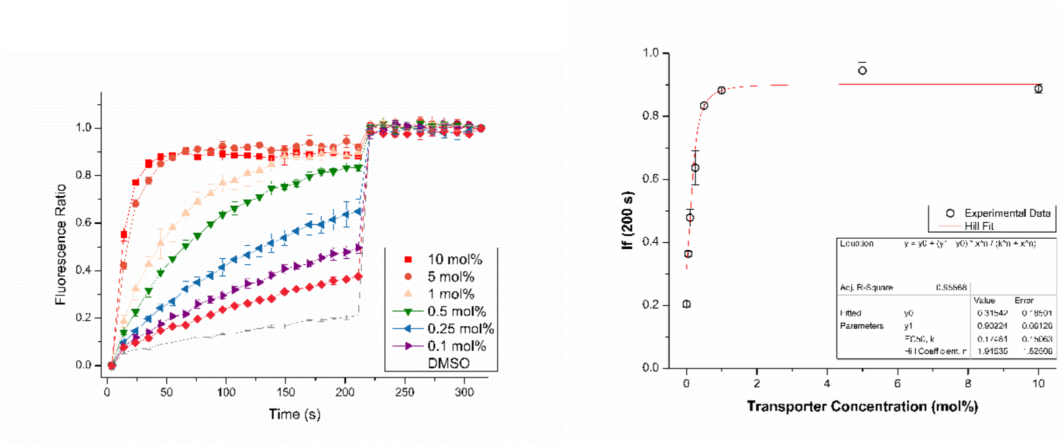

Figure 99. Hill analysis of H$^+$/Cl$^-$ symport (or Cl$^-$/OH$^-$ antiport) facilitated by mono-n-pentyl di-t-pentyl tren in the NMDG-Cl assay with 'fatty acid free conditions' in BSA treated vesicles.

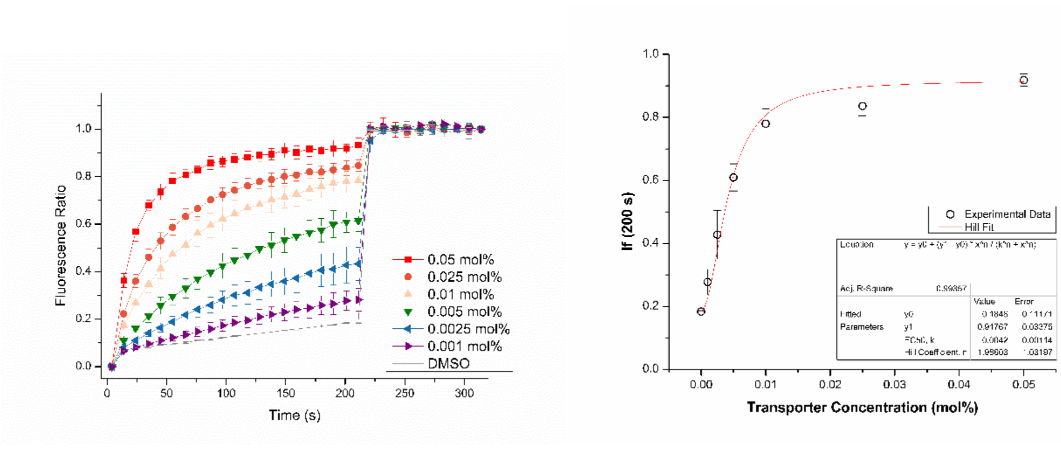

Figure 100. Hill analysis of H$^+$/Cl$^-$ symport (or Cl$^-$/OH$^-$ antiport) facilitated by a combination of gramicidin and mono-n-pentyl di-t-pentyl tren in the NMDG-Cl assay.

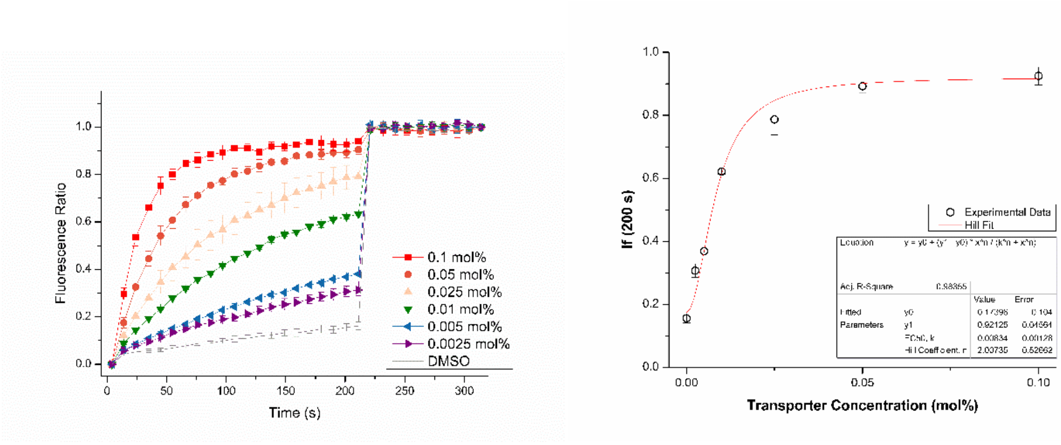

Figure 101. Hill analysis of H$^+$/Cl$^-$ symport (or Cl$^-$/OH$^-$ antiport) facilitated by a combination of oleic acid and mono-n-pentyl di-t-pentyl tren in the NMDG-Cl assay.

Macrocycle tren

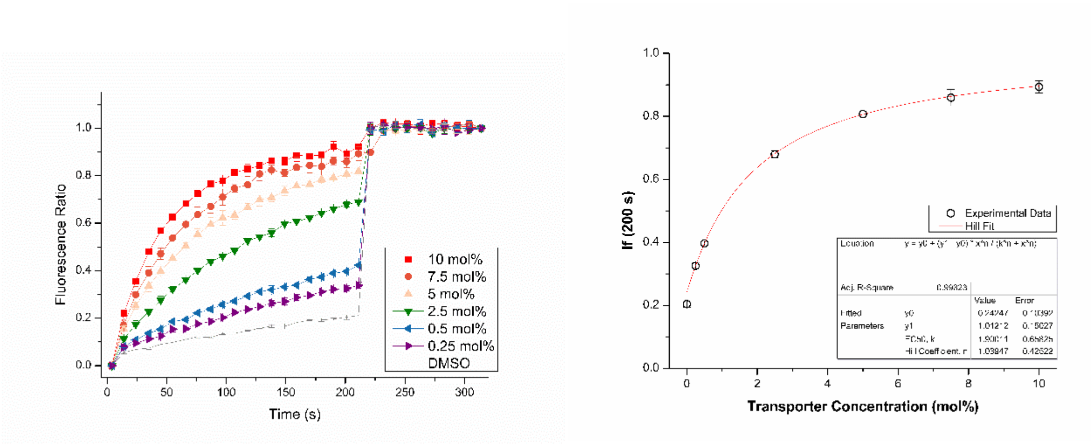

Figure 102. Hill analysis of H^+/Cl^- symport (or Cl^-/OH^- antiport) facilitated by macrocycle tren in the NMDG-Cl assay with ‘fatty acid free conditions’ in BSA treated vesicles.

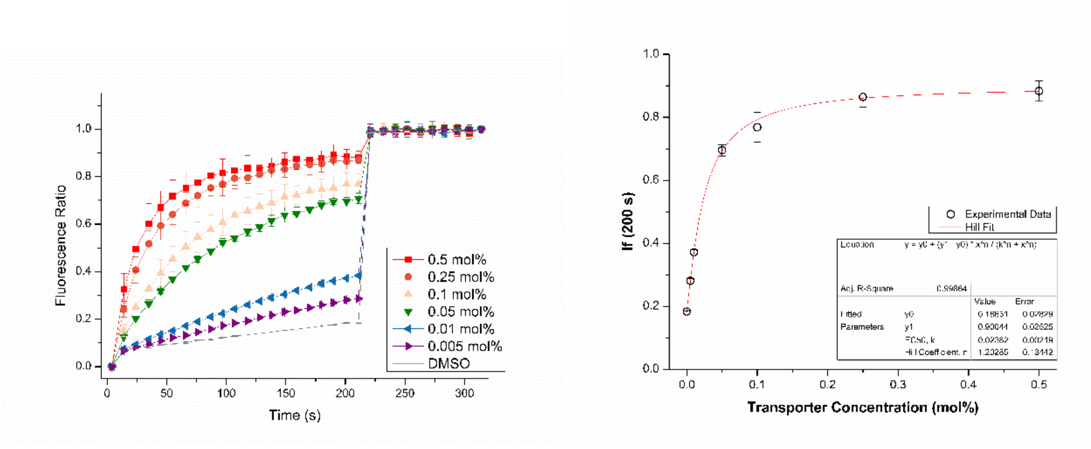

Figure 103. Hill analysis of H^+/Cl^- symport (or Cl^-/OH^- antiport) facilitated by a combination of gramicidin and macrocycle tren in the NMDG-Cl assay.

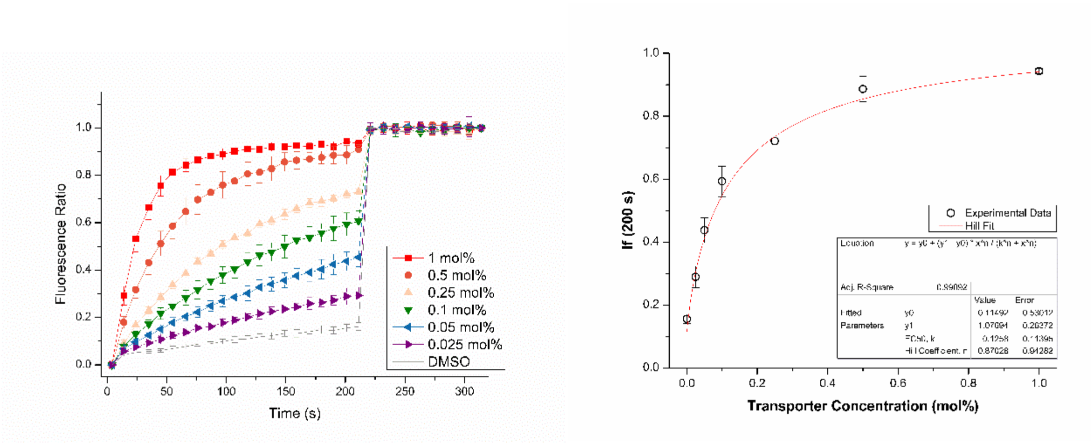

Figure 104. Hill analysis of H^+/Cl^- symport (or Cl^-/OH^- antiport) facilitated by a combination of oleic acid and macrocycle tren in the NMDG-Cl assay.
